# CRISPR/Cas9-Induced Loss-of-Function Mutation in the Barley *Mitogen-Activated Protein Kinase 6* Gene Causes Abnormal Embryo Development Leading to Severely Reduced Grain Germination and Seedling Shootless Phenotype

**DOI:** 10.3389/fpls.2021.670302

**Published:** 2021-07-30

**Authors:** Pavel Křenek, Elizaveta Chubar, Pavol Vadovič, Ludmila Ohnoutková, Tomáš Vlčko, Véronique Bergougnoux, Petr Cápal, Miroslav Ovečka, Jozef Šamaj

**Affiliations:** ^1^Centre of the Region Haná for Biotechnological and Agricultural Research, Department of Cell Biology, Faculty of Science, Palacký University Olomouc, Olomouc, Czechia; ^2^Laboratory of Growth Regulators, Faculty of Science, Institute of Experimental Botany of the Czech Academy of Sciences, Palacký University Olomouc, Olomouc, Czechia; ^3^Centre of Region Haná for Biotechnological and Agricultural Research, Czech Advanced Technology and Research Institute, Palacký University Olomouc, Olomouc, Czechia; ^4^Centre of the Region Haná for Biotechnological and Agricultural Research, Institute of Experimental Botany of the Czech Academy of Sciences, Olomouc, Czechia

**Keywords:** barley, *Hordeum vulgare* L., mitogen-activated protein kinase 6, MPK6, CRISPR/Cas9, lethality, abnormal embryo, shootless phenotype

## Abstract

The diverse roles of mitogen-activated protein kinases (MAPKs, MPKs) in plant development could be efficiently revealed by reverse genetic studies. In *Arabidopsis, mpk6* knockout mutants complete the life cycle; however, ~40% of their embryos show defects in the development leading to abnormal phenotypes of seeds and seedlings' roots. Contrary to the *Arabidopsis MPK6*, the rice *MPK6* (*OsMPK6*) is an essential gene as transfer DNA (T-DNA) insertion and CRISPR/Cas9 induced loss-of-function mutations in the *OsMPK6* cause early embryo arrest. In this study, we successfully developed a viable transgenic barley line with the CRISPR/Cas9-induced heterozygous single base pair cytosine-guanine (CG) deletion [wild type (WT)/−1C] in the third exon of the *HvMPK6* gene, a barley ortholog of the *Arabidopsis* and rice *MPK6*. There were no obvious macroscopic phenotype differences between the WT/−1C plants and WT plants. All the grains collected from the WT/−1C plants were of similar size and appearance. However, seedling emergence percentage (SEP) from these grains was substantially decreased in the soil in the T2 and T3 generation. The mutation analysis of the 248 emerged T2 and T3 generation plants showed that none of them was a biallelic mutant in the *HvMPK6* gene, suggesting lethality of the −1C/−1C homozygous knockout mutation. In the soil, the majority of the −1C/−1C grains did not germinate and the minority of them developed into abnormal seedlings with a shootless phenotype and a reduced root system. Some of the −1C/−1C seedlings also developed one or more small chlorotic leaf blade-like structure/structures. The −1C/−1C grains contained the late-stage developed abnormal embryos with the morphologically obvious scutellum and root part of the embryonic axis but with the missing or substantially reduced shoot part of the embryonic axis. The observed embryonic abnormalities correlated well with the shootless phenotype of the seedlings and suggested that the later-stage defect is predetermined already during the embryo development. In conclusion, our results indicate that barley MPK6 is essential for the embryologically predetermined shoot formation, but not for the most aspects of the embryo and early seedling development.

## Introduction

Cultivated barley (*Hordeum vulgare* L.) is a member of the *Poaceae* family, and together with other cereal crops, it belongs to the most grown plant species in the world. Barley is widely used for animal feed, as human food, and in the brewing industry (Mrízová et al., 2014; Harwood, 2019), and it is also recognized as a genomic model of the *Triticeae* tribe (Saisho and Takeda, 2011). Grain size and number per plant are the key agronomical traits contributing to grain yield in cereals. Therefore, circumstances and molecular players influencing these traits are of high research priority. Mitogen-activated protein kinases (MAPKs) are important phospho-specific enzymes, which control the seed size and number in plants (Bush and Krysan, 2007; Liu et al., 2015a; Guo et al., 2018). Generally, plant MAPKs are involved in many cellular, physiological, and developmental processes (Komis et al., 2018), and in many diverse responses to abiotic and biotic stress stimuli (Abass and Morris, 2013; Kawasaki et al., 2017).

MAPKs are organized in the conserved signaling modules, which transmit cellular signals within the cell as a response to external or endogenous developmental stimuli. This signal transfer occurs *via* phosphorylation through three-tiered components comprising MAPK kinase kinase (MAPKKK), MAPK kinase (MAPKK), and MAPK (Šamajová et al., 2013; Komis et al., 2018). The *in silico* analysis that was performed in the *Triticeae* sequence database identified 152 members of the MAPK family (Goyal et al., 2018). In rice, a similar analysis revealed 75 members of the MAPKKK family (Rao et al., 2010) and 15 members of the MAPK family (Hamel et al., 2006). In a previous study on barley, we have identified 16 members of the MAPK family (Křenek et al., 2015). In rice, it was found that OsMPK6 is important for grain size, cell proliferation, and homeostasis because *dwarf and small grain 1* (*dsg1*) mutant, a partial *Osmpk6* loss-of-function mutant, showed small grains, dwarfism, and erect leaves (Liu et al., 2015a; Xu et al., 2018). Moreover, it was found that rice *grain size and number 1* (*gsn1*) mutant defective in MAPK phosphatase (OsMKP1) has larger grains and sparser panicle than WT (Guo et al., 2018). When OsMKP1 is in the active form, it dephosphorylates OsMPK6, resulting in denser panicle and smaller grains (Guo et al., 2018). A more recent study showed that the MAPK cascade OsMKKK10-OsMKK4-OsMPK6 is involved in determining the spikelet number per panicle in rice by regulating cytokinin homeostasis (Guo et al., 2020). When a gene for the rice (ROP) GTPase (OsRac1), which interacts with OsMPK6, was overexpressed in rice, it resulted in a higher grain weight and width and a larger spikelet hull (Zhang et al., 2019). The authors assumed that grain phenotype is directly connected to OsMPK6 and to its role in the regulation of cell division and grain size.

It is well-known that ~16–44% of the *Arabidopsis mpk6* mutant seedlings temporarily show “no primary root phenotype” while the rest of the *mpk6* mutant seedlings displays inhibition of the formation and growth of their roots (Müller et al., 2010). Some of these early postembryonic defects are caused by the abnormalities in root cellular and tissue patterning resulting from ectopic cell divisions and the shifted cell division planes (Müller et al., 2010). In addition, AtMPK6 was found to act downstream of YODA, which regulates postembryonic root development, likely through auxin-controlled mechanisms (Smékalová et al., 2014). Apart from root development, YODA-MKK4/5-MPK3/6 cascade is recognized as a major regulator of *Arabidopsis* embryo development (Lukowitz et al., 2004). This is supported by the fact that ~40% of the embryos of the *Arabidopsis mpk6* mutant exhibit serious defects in the development, which are maternally determined (Bush and Krysan, 2007; Zhang et al., 2017). Also, the fertility of the *mpk6* mutants is reduced because of abnormal anther development (Bush and Krysan, 2007).

Taken together, the rice and *Arabidopsis* MPK6 play important roles in cell division, embryo development, and the control of seed size and number. However, to our knowledge, there has been no detailed study about the impact of MPK6 on the development and reproduction of a *Triticeae* cereal crop until now. In this work, we provide results suggesting new crucial roles of MPK6 in the embryonic and early seedling development of barley.

## Materials and Methods

### Plant Material and Cultivation

Immature embryos of spring barley (*H. vulgare* L.) variety Golden Promise (GP) and a double haploid line of GP were used for the preparation of stably transformed transgenic barley lines using the published protocols (Harwood, 2014; Marthe et al., 2015). Single selected vital transgenic GP line with a CRISPR/Cas9-edited *HvMPK6* gene and wild-type (WT; control) GP plants were used for the experiments. In the transgenic line, which was designated as line A, a monoallelic single base pair CG deletion is present in the third nucleotide position upstream of the protospacer adjacent motif (PAM) site in the third exon of the *HvMPK6* gene. Rarely, this line may harbor also other, mostly frame-shift monoallelic mutations, in a similar position as CG deletion. Also, CC9-K6E3 cassette is present in the genome of line A. Immature embryo GP donor plants were cultivated under controlled environmental conditions in phytotron (Weiss-Gallenkamp, Loughborough, UK) at 15/12°C with a 16-h photoperiod with light levels of 400–500 μmol.m^−2^.s^−1^ at the mature plant canopy level and 70% relative humidity. The double haploid donor plants were grown in three steps according to Marthe et al. (2015). In the tiller elongation, plants were transferred to phytotron (Weiss-Gallenkamp, Loughborough, UK) maintained at 18/16°C with a 16-h photoperiod (500 μmol.m^−2^.s^−1^ at the top of mature plants) and 60% relative humidity. Cool white fluorescent tubes (Philips Master tl-d 58W/840) supplemented with clear incandescent light bulbs (Crompton 40W Cooker Hood Lamp, Bradford, UK) provided lightning. GP donor plants were grown in 1-L pots containing the 1910383 Topf+LF30+TonXL professional substrate (Gramoflor, Vechta, Germany) supplemented with additional perlite (Perlit Ltd., Šenov u Nového Jičína, Czech Republic) and fertilized several times with CERERIT^®^ GSH (Lovochemie, Lovosice, Czech Republic) during vegetation. Double haploid donor plants were grown in 2-L pots containing white and black peat (Klassman substrate 2, Klasmann-Deilman, Geeste, Germany), mixed with garden compost and sand (2:2:1), and fertilized once with 15 g of Plantacote 6M Top N (Aglukon, Düsseldorf, Germany) during tiller elongation, and then regularly watered with a 1% Hakaphos solution (Compo Expert, Münster, Germany) during spike development. Transgenic and control plants were cultivated under the same growing conditions as GP embryo donor plants except for the temperature conditions, which were changed to 17°C/14°C, lightning intensity, which was decreased to ~250 μmol.m^−2^.s^−1^ in the seedling level, and pot size and type, which differed according to the purpose of cultivation. For selecting Cas9-edited plants, evaluating seedling emergence percentage (SEP), and analyzing the status of the grains in the soil, plants were grown in the six-pot plastic planters. In the six-pot planters, seeds were sown at 2-cm depth, and the status of the grains was analyzed in the non-fertilized soil. To propagate the grains, plants were grown in 1-L pots.

### Computational Characterization of the *HvMPK6* Gene

Previously, we identified *in silico* the longest full-length complementary DNA (cDNA) clone (AK376245) coding for complete HvMPK6 protein (394 aa) (Křenek et al., 2015). Originally, HvMPK6 was named HvMPK1 (Křenek et al., 2015). However, the HvMPK6 designation is used herein, to follow the recently accepted nomenclature for the *Triticeae* MAPK family (Goyal et al., 2018). In this nomenclature, *Triticeae* MAPKs are predominantly numbered based on their homology to *Arabidopsis* MAPKs. Accordingly, HvMPK6 is a homolog of a well-studied AtMPK6 (e.g., Bush and Krysan, 2007; Wang et al., 2007; Müller et al., 2010).

The nucleotide sequence of AK376245 was retrieved from the National Center for Biotechnology Information (NCBI) website (https://www.ncbi.nlm.nih.gov/nuccore/AK376245.1?report=fasta) and used as a query in BLASTN (Altschul et al., 1990) or GMAP searches of the recently released GP v1 genome assembly (Schreiber et al., 2020; https://ics.hutton.ac.uk/gmapper/blast_page.html, https://ics.hutton.ac.uk/gmapper/), the Morex IBSC_v2 genome assembly (Mascher et al., 2017; https://plants.ensembl.org/Multi/Tools/Blast?db=core#), and Barley Reference Transcript Dataset BaRTv1.0 (Rapazote-Flores et al., 2019; https://ics.hutton.ac.uk/barleyrtd/blast_page.html). The query coverage by the target sequence and percentage of the nucleotide identity was inspected manually from the BLASTN outputs. Genomic sequence of the transcribed region of the *HvMPK6* gene was retrieved from the GP v1 genome assembly, and the exon–intron structure of the gene was determined by the mapping of the AK376245 nucleotide sequence onto the retrieved genomic sequence using the Splign tool (Kapustin et al., 2008) available at NCBI (https://www.ncbi.nlm.nih.gov/sutils/splign/splign.cgi). Based on this analysis, the genomic sequence was edited in the ApE plasmid editor (https://jorgensen.biology.utah.edu/wayned/ape/) and used for the visualization of the transcribed region of the *HvMPK6* gene with the Exon–Intron Graphic Maker (http://wormweb.org/exonintron). The splicing variants of the HORVU7Hr1G023760 gene were compared by using a “transcript comparison” function of the EnsemblPlants gene interface and mapped together with BART1_0-u50115.001 and BART1_0-u50115.00 transcripts to the retrieved GP genomic region of the *HvMPK6* gene with Splign. Based on this analysis, the genomic sequences of the transcript variants were edited in the ApE editor and aligned with Clustal Omega (Sievers et al., 2011; https://www.ebi.ac.uk/Tools/msa/clustalo/) by using default parameters of the DNA alignment interface. Introns in the resulting multiple sequence alignment were shortened in the BioEdit version 7.2.6.1. (https://bioedit.software.informer.com/), and the shortened alignment was subsequently processed by Mega X (Kumar et al., 2018; https://www.megasoftware.net/dload_win_gui) to produce a final alignment in the Nexus (PAUP 4.0) format. Fasta files of the aminoacid sequences of HvMPK6 (BAK07440.1) and the *Arabidopsis* MPK6 (OAP09870.1) were downloaded from the NCBI website and aligned by using the protein–protein BLAST suite (https://blast.ncbi.nlm.nih.gov/Blast.cgi).

### Design and Molecular Cloning of the CRISPR/Cas9 Construct for *HvMPK6* Targeting

The guide RNA (gRNA) on-target sequence was triple selected within the third exon of the *HvMPK6* gene by using DESKGEN KNOCKIN (https://www.deskgen.com/guidebook/ki.html), CRISPRdirect (Naito et al., 2015) (https://crispr.dbcls.jp/), and CRISPOR (Concordet and Haeussler, 2018) (http://crispor.tefor.net/) tools considering high specificity and efficiency of the DNA cleavage. In the selected on-target sequence, the first nucleotide of the PAM and four nucleotides immediately upstream of the PAM site generate the *Hpy*188I restriction site allowing for the PCR-restriction enzyme (PCR-RE) mutation genotyping. The CRISPR/Cas9 construct for *HvMPK6* targeting was essentially prepared as described before (Budhagatapalli et al., 2016). Briefly, *HvMPK6* spacer oligonucleotides sK6E3_F and sK6E3_R (Supplementary Table 1) were annealed, and the obtained oligonucleotide heterodimer was ligated into the *Bsa*I-digested vector pSH91. The resulting gRNA-Cas9 expression cassette of pSH91 was sequenced by Sanger sequencing (SEQme, Dobříš, Czech Republic), released from the pSH91 backbone by *Sfi*I endonuclease, and subcloned into the *Sfi*I-digested p6i-d35S-TE9 vector (DNA-Cloning-Service, Leipzig, Germany) to generate the construct CC9-K6E3. The final construct was verified by Sanger sequencing (SEQme) and electroporated into *Agrobacterium tumefaciens* strain AGL1. Liquid cultures of *A. tumefaciens* clones containing the CC9-K6E3 construct were mixed in a 1:1 ratio with glycerol (50% solution, v/v) and stored at −80°C.

### Development of Transgenic Barley Lines

Immature embryos of the barley cultivar GP were transformed essentially as described by Harwood (2014) with the exception that hygromycin (Roche, Mannheim, Germany) concentration was decreased to 30 mg l^−1^. To further increase the number of regenerated plants, hygromycin was omitted in the regeneration medium and in one of the three rounds of the transformations in the callus induction medium in the first stage of selection. Immature embryos of the dihaploid GP were transformed according to Marthe et al. (2015) without modification.

### Ploidy Estimation of Transgenic Plants

Samples for the ploidy measurement were prepared according to Doležel et al. (2007). In brief, ~20 mg of fresh leaf tissue was chopped in 0.5-ml Otto I buffer with a razor blade and filtered through 50-μm nylon mesh into a sample tube. Samples were then diluted with 1-ml Otto II buffer, stained with 50 μg/ml propidium iodide, and treated with 50 μg/ml RNase A for 5 min on ice before the analysis. Samples were analyzed on the Sysmex CyFlow Space instrument (Sysmex, Münster, Germany) by using the FloMax software. More than 2,000 particles were analyzed for each sample.

### Genomic DNA Extraction

Genomic DNA was extracted from the basal leaf segments of the youngest fully developed leaves of barley plants (BBCH 10-53), roots, leaf blade-like structures, and grains' leftovers (without husks) of the seedlings removed from the soil, and grains (without husks) removed from the soil and embryos and/or endosperms with most of the intact bran (remaining after the embryo extirpation) of the mature imbibed grains using the cetyltrimethylammonium bromide (CTAB). Husks were removed manually from the grains and grain leftovers before the DNA extarction procedure. DNA extraction was performed by using either 2-ml Eppendorf tubes or 1.2-ml racked collection microtubes (Qiagen, Hilden, Germany) depending on the number and type of samples processed.

### Genotyping of Barley Plants for the Presence of CC9-K6E3 Transfer DNA by TaqMan^TM^ Multiplex qPCR

The presence of the CC9-K6E3 cassette in the genomic DNA of regenerated plants and their emerged progeny was determined by using the TaqMan Multiplex Real-Time PCR (TaqMan Multiplex qPCR) as described before (Bartlett et al., 2008) with some modifications. Briefly, the analysis was carried out by using *CO2* (*CONSTANS-LIKE 2*, AF490469) gene-specific primers and probe described by Bartlett et al. (2008) and *HPT* gene-specific primers and probe designed in this work (Supplementary Table 1). The primers/probe set for the *HPT* gene was designed using the Primer3 software (v. 0.4.0) (https://bioinfo.ut.ee/primer3-0.4.0/), and the specificity of the designed primers was verified by the PrimerBLAST software (Ye et al., 2012) against *Hordeum* “nr” database (https://www.ncbi.nlm.nih.gov/tools/primer-blast/). The assay contained 5 μl of ABGene Absolute QPCR Rox Mix (Cat No. AB1139, Thermo Fisher Scientific, Waltham, MA, USA), 2.5 μl of the mixture of four primers and two probes (each primer or probe at final 200 nM concentration), and 2.5 μl of diluted genomic DNA. PCRs were carried out in StepOnePlus™ Real-Time PCR cycler (Thermo Fisher Scientific, Waltham, MA, USA). The instrument was set up for the detection of FAM-TAMRA, VIC-TAMRA, and ROX internal passive reference dye. The PCR cycling conditions were initiated at 95°C for 15 min for the hot start polymerase activation, followed by 40 cycles at 95°C for 15 s and at 60°C for 60 s.

### On-Target Mutation Analysis

About 316-bp genomic DNA fragment covering the on-target region of the *HvMPK6* gene was amplified from the extracted genomic DNA of regenerated plants and their emerged progeny, seedlings removed from the soil, and embryos and/or endosperms with most of the intact bran of the imbibed mature grains using PCR with primers K6E3_F1/K6E3_R1 (Supplementary Table 1). The obtained PCR products were sequenced by Sanger sequencing (SEQme) using the K6E3_F1 primer in the sequencing reaction [PCR sequencing (PCR-Seq)] and/or digested with the *Hpy*188I restriction enzyme (PCR-RE). Sequencing chromatograms were evaluated manually and aligned with the nucleotide sequence of the reference DNA fragment of the *HvMPK6* gene in the ApE plasmid editor. Superimposed sequencing chromatograms associated with the heterozygous mutants in the *HvMPK6* gene were decoded into allelic sequences by using the DSDecodeM tool (Liu et al., 2015b; http://skl.scau.edu.cn/dsdecode/). We could not reliably decode only three of such chromatograms. The respective three PCR amplicons and also 16 other independent PCR amplicons [two amplicons associated with multiple genotype chimeras and 14 amplicons, which were mostly predicted to contain more extensive mutations (larger deletions or complex mutations), and also the most common CG deletion (−1C allele)] were cloned into the pCR^TM^2.1 vector (Thermo Fisher Scientific). Sanger sequencing (SEQme) of several pCR^TM^2.1 clones for each amplicon confirmed the accuracy of the decoding procedure in all cases and revealed the sequences of the *HvMPK6* alleles associated with the three unpredictable chromatograms and two multiple genotype chimeras. Also, we manually decoded a sequence of the mutated *Hvmpk6* allele of one chimera comprised more of WT cells than of mutated cells and sequences of the WT *HvMPK6* and −1C *Hvmpk6* alleles of the two multiple genotype chimeras. To analyze *in silico* the effect of the induced mutations of A1 and A2 plants on HvMPK6 protein synthesis, the mutations were individually introduced into the AK376245.1.mrna1_gpv1_chr7H_26521825_26529501_0 *HvMPK6* messenger RNA (mRNA) nucleotide sequence in the ApE editor. The reference and mutated nucleotide sequences were translated in the ApE editor, and the resulting amino acid sequences were aligned by using COBALT (https://www.ncbi.nlm.nih.gov/tools/cobalt/cobalt.cgi).

### Microscopic Observation and Imaging

The mature T3-generation grains of heterozygous mutant (WT/−1C) transgenic plants and the mature grains of WT non-transgenic control plants were surface sterilized in 1% (v/v) sodium hypochlorite for 2 min upon shaking. After five times of washing in sterile Milli-Q water, grains were imbibed in sterile Milli-Q water in darkness at 21°C for 18 or 24 hours (h) before embryo extirpation. Following imbibition, grains were kept moistened in Petri dishes until the end of the embryo extirpation. Embryos were extirpated by using forceps and scalpel under the table binocular stereomicroscope (A. Knuss, Optronic, Hamburg, Germany) and placed by scutellum site down on 1% (w/v) micro agar (Duchefa Biochemie B.V., Haarlem, The Netherlands) in a covered Petri dish. Embryos from 47 to 50 grains of a single genotype were extirpated at once in a maximum period of 1 h and 45 min and, after this time, immediately observed and imaged by using the stereo zoom microscope AxioZoom.V16 (Zeiss, Oberkochen, Germany). All abnormal and selected transgenic embryos of normal appearance and WT embryos were imaged scutellum side down and also some of these embryos were imaged scutellum side up. Also, selected grains and developing seedlings were observed and imaged by using AxioZoom.V16 (Zeiss). Imaging was performed with the Plan NeoFluor Z 1.0× objective and the Axiocam 105 color camera (Zeiss, Oberkochen, Germany) in a Z-stack mode with optical sections of 36-μm to 156-μm thick and exposure time from 24.92 ms to 55 ms. The number of optical sections and zoom factor were adjusted differently to the extirpated embryos, grains, or developing seedlings according to particular sample volumetric dimensions. Samples were illuminated with an LED ring-light source for a homogenous incident illumination during imaging. The images acquired in serial optical sections were processed with the extended depth of focus function in the Zen blue 2.3 pro software (Zeiss, Oberkochen, Germany) and exported as.tiff formats for figure assembling in PowerPoint 2019 (Microsoft, Redmond, WA, USA).

### Immunoblotting Analysis and Evaluation of the Specificity of the C-terminal Epitope of AtMPK6

The specificity of the commercially available Anti-AtMPK6 antibody (#A7104, Sigma-Aldrich, St. Louis, MO, USA) toward HvMPK6 was evaluated by querying the amino acid epitope sequence “REALAFNPEYQQ” against the barley database “*Hordeum vulgare* IBSC_v2 (Proteins)” by using the BLASTP interface of EnsemblPlants (http://plants.ensembl.org/Multi/Tools/Blast). The parameters were set to “Normal” search sensitivity, “100” maximum numbers of alignments displayed, “100” maximum numbers of scores displayed, “BLOSUM90” scoring matrix, “1,000” *E*-value threshold, non-filtering of low complexity regions, and otherwise default parameters. The gene hits were further annotated by using Barley Reference Transcript Dataset BaRTv1.0.

For the immunoblot analysis, the T3-generation mature grains of heterozygous mutant (WT/−1C) transgenic plants and the mature grains of WT non-transgenic control plants were sterilized, imbibed for 24 h, cultivated after imbibition and subjected to the embryo extirpation as described above. Following embryo extirpation, selected endosperms with most of the intact bran and husk remaining after the embryo extirpation (further shortened to “endosperm/ endosperms with bran and husk) were either immediately frozen (mock) or immediately squeezed and incubated at a room temperature for 15 min before freezing (wounding). *Hvmpk6* mutant (−1C/−1C) endosperms with bran and husk were selected for the immunoblot analysis based on the abnormal morphology of the respective extirpated embryos and subsequent PCR-based genotyping of these embryos for on-target mutations. Endosperms with bran and husk were individually homogenized in liquid nitrogen to a fine powder, and subjected to protein extraction as described by Takáč et al. (2017). Each protein sample was extracted from one endosperm with bran and husk. Proteins were separated by sodium dodecyl sulfate–polyacrylamide gel electrophoresis (SDS-PAGE) (MINI-Protean II cell system, Bio-Rad, Hercules, CA, USA) on 12% gels. Identical protein concentrations were loaded for each sample. Separated proteins were transferred onto polyvinylidene difluoride (PVDF) membrane (GE Healthcare, Chicago, IL, USA) in a wet tank unit (Bio-Rad, Hercules, CA, USA) at 24 V overnight by using transfer buffer [25 mM Tris, 192 mM glycin, 10% (v/v) methanol].

For the immuno-detection of phosphorylated MAPK proteins, the membrane was blocked in 4% (w/v) bovine serum albumin (BSA) in Tween-20 supplemented Tris-buffered-saline [TBS: 100 mM Tris-HCl, pH 7.4, 150 mM NaCl, 0.1% (v/v) Tween-20] at 4°C overnight. Subsequently, the membrane was incubated with a polyclonal antibody against mammalian phosphorylated ERK1/2 (pERK; #9101, Cell Signaling, Danvers, MA, USA) at 4°C overnight. The antibody was diluted 1:1,000 in TBS-T containing 1% (w/v) BSA. Following the five washing steps in TBS-T, the membrane was incubated with a horseradish peroxidase-conjugated goat anti-rabbit IgG secondary antibody (#A24531, Santa Cruz Biotechnology, Dallas, TX, USA) at room temperature for 1.5 h. The antibody was diluted 1:5,000 in TBS-T containing 1% (w/v) BSA. After the three washing steps in TBS-T, proteins were detected by incubating the membrane in Clarity Western ECL substrate (Bio-Rad, Hercules, CA, USA). Luminescence was detected by using the Chemidoc MP documentation system (Bio-Rad, Hercules, CA, USA). After the protein detection using a pERK antibody, the membrane was restriped according to Takáč et al. (2021). The restriped membrane was blocked in a mixture of 4% (w/v) low-fat dry milk and 4% (w/v) BSA supplied into TBS-T at 4°C overnight. Subsequently, the membrane was incubated with an anti-AtMPK6 antibody (Sigma-Aldrich) at 4°C overnight. The antibody was diluted 1:5,000 in TBS-T containing 1% (w/v) BSA. The next steps were identical to the protocol used for a pERK antibody. Immunoblot analysis was repeated six times with different biological samples.

### Off-target Mutation Analysis

Potential off-targets of the selected guide sequence CTCAAATCAAGCTTTATCGG in the GP (GCA_902500625.1; Schreiber et al., 2020) and Morex (GCA_901482405.1; Mascher et al., 2017) genome assembly were analyzed *in silico* by using the CRISPOR website. Off-target detection involved the sequences flanked by one of the three motifs: NGG, NAG, NGA, and allowed at most four mismatches. Complete GP and Morex genomes, exons, and introns only were analyzed for the potential unintended targets. To determine exonic and intronic off-targets in the GP genome, nucleotide sequences of all GP off-targets were downloaded from the CRISPOR website and queried against the GP genome assembly by using the EnsemblPlants BLASTN interface (http://plants.ensembl.org/Multi/Tools/Blast) set to “Short sequences” search sensitivity, *E*-value threshold 1e-2, Match/mismatch scores 1, −2, and otherwise default parameters. The only gene hits with 100% identity and 100% query coverage were considered as containing an off-target site. The exonic and intronic off-targets in the Morex genome were identified by using the CRISPOR website. Among them, only those identical to the predicted GP off-targets were considered as potential off-targets. To determine particular locations of the exonic and intronic off-targets, their nucleotide sequences or reverse complement nucleotide sequences were queried against “Sequence” (GP) or “Transcript comparison” (Morex) interface of the respective EnsemblPlants gene models using a find bar of the Mozilla Firefox browser (Mozilla, San Francisco, CA, USA). GP *MAPK* genes were identified by querying nucleotide sequences of the 20 recently described Morex *MAPK* genes (Cui et al., 2019) against GP GCA_902500625.1 genome assembly using GMAP utility (https://ics.hutton.ac.uk/gmapper/gmap_page.html). We found that the Morex *MAPK* gene models HORVU4Hr1G049430.1 and HORVU6Hr1G021480.1 are redundant as well as HORVU6Hr1G068270.1 and HORVU0Hr1G016660.4. Therefore, only 18 *MAPK* genes were identified in the GP genome assembly. The occurrence of the off-targets in the genomic regions surrounding the identified *MAPK* genes was analyzed in Excel 2019 (Microsoft). Initially, GP off-targets and *MAPK* genes were sorted into groups according to their chromosomal locations. Subsequently, within each chromosomal group, the distance of the *MAPK* genes/gene from the off-targets was calculated by subtracting the genomic coordinates of the *MAPK* genes/gene from the genomic coordinates of the off-targets.

A 344-bp genomic DNA fragment covering the CABVVH010000001.1 215.57 Mbp off-target region and a 460-bp genomic DNA fragment covering the CABVVH010000002.1 489.51 Mbp off-target region were amplified from the genomic DNA of the emerged T3-generation A1 plants by PCR with the primer pair Off215_F2/Off215_R2 and Off489_F2/Off489_R2, respectively (Supplementary Table 1). PCR products were sequenced by Sanger sequencing (SEQme) using Off215_F1 primer or Off489_R1 primer in the sequencing reactions (Supplementary Table 1). DNA sequencing chromatograms (.ab1 files) were aligned with the respective reference nucleotide sequences and evaluated manually in the SeqMan Pro utility of the Lasergene analysis package (DNASTAR Inc., Madison, WI, USA).

## Results

### Computational Identification of the *HvMPK6* Gene and Associated Transcripts

**Figure 1:**
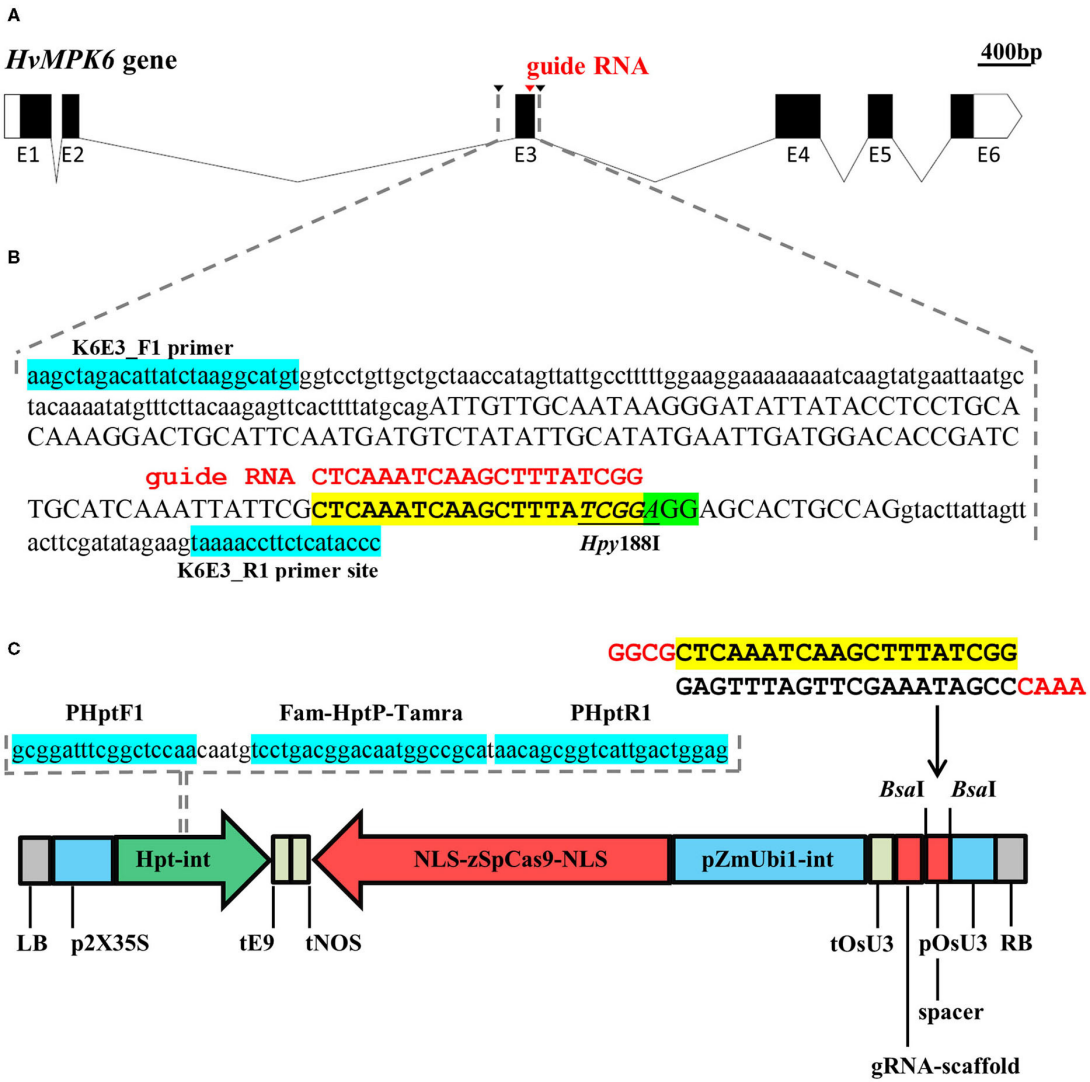
Schematic overview of the targeting of the *HvMPK6* gene with a CRISPR/Cas9 technology and on-target mutation detection. **(A)** Structure of the transcribed region of the Golden Promise (GP) *HvMPK6* gene (AK376245.1.mrna1_gpv1_chr7H_26521825_26529501_0, https://ics.hutton.ac.uk/gmapper/). Black rectangles indicate coding exons or coding parts of the exons and open rectangles indicate 5′ and 3′ untranslated regions (UTRs). Exons are indicated with “E” and exon number below the rectangles. Introns are shown as solid lines. The on-target site of the designed guide RNA (gRNA) is indicated by a red arrowhead in the coding region of the third exon (E3). Black arrowheads indicate positions of the K6E3_F1 and K6E3_R1 primers used for the PCR-based detection of on-target mutations. **(B)** Schematic representation of the 316-bp genomic DNA fragment covering the on-target region. Dashed gray lines indicate the position of the fragment in **(A)** drawing. The nucleotide sequence of the respective GP *HvMPK6* gene fragment (plus strand) is shown with the exon three sequence in an upper case and the surrounding intron sequences in a lowercase. The position of the gRNA on-target site and the protospacer adjacent motif (PAM) site is shown with a yellow and green box, respectively. A 20-bp gRNA sequence is typed in red and shown above the on-target site. The diagnostic restriction site of the *Hpy*188I endonuclease is underlined. Turquoise boxes indicate flanking positions of the K6E3_F1 and K6E3_R1 primers. **(C)** Schematic representation of the CC9-K6E3 construct used for the stable *Agrobacterium tumefaciens*-mediated transformation of barley. The construct is based on pSH91 and p6i-d35S-TE9 vectors. The sequence of the *HvMPK6* spacer oligonucleotide heterodimer is shown above the construct drawing with a 20-bp gRNA indicated in a yellow box and 5′ overhangs typed in red. The spacer was cloned between the *Bsa*I sites of the construct (indicated with arrows) to allow for the expression of gRNA (including gRNA-scaffold) under the control of the *Oryza sativa* snRNA U3 promoter (pOsU3) and terminator (tOsU3). The expression of the *Zea mays* codon optimized version of the *Streptococcus pyogenes Cas9* endonuclease gene (NLS-zSpCas9-NLS) is driven by the maize polyubiquitin gene promoter 1 including intron (pZmUbi1-int) and a nopaline synthase terminator (tNOS). Double cauliflower mosaic virus promoter (p2X35S) and rbcS-E9 gene terminator (tE9) control the expression of the hygromycin phosphotransferase gene including modified IV2 intron (HPT-int). NLS, nuclear localization signal; LB, left border; RB, right border. The nucleotide sequence of the *HPT* gene DNA fragment, which was used as a template for multiplex TaqMan quantitative PCR- (qPCR-) based transfer DNA (T-DNA) genotyping, is shown above the construct. Dashed lines indicate the location of the fragment in the construct. Positions of the primers and TaqMan probe are shown with turquoise boxes.

We identified a 7,677-bp genomic region fully covering the transcribed part of the *HvMPK6* gene in the barley cv. GP reference genome assembly (AK376245.1.mrna1_gpv1_chr7H_26521825_26529501_0, Supplementary Figure 1). Based on the computational analysis of this region, the GP *HvMPK6* gene was predicted to contain six exons and five introns (Supplementary Figure 1 and Figure 1A). The predicted exons are fully sequenced and provide 100% coverage of the sequence of the AK376245 reference transcript with the nucleotide identity ranging from 95 to 100%. The only unsequenced region is present in the proximal end of the second intron of the *HvMPK6* gene (Supplementary Figure 1). We also identified the *HvMPK6* gene in the Ensembl Plants IBSC_v2 Morex genome assembly (IBSC_v2:chr7H:37299401:37308100:1, HORVU7Hr1G023760). Overall, HORVU7Hr1G023760 has the same exon/intron structure as we predicted for the transcribed portion of the GP *HvMPK6* gene. However, the first 196 bp of the first exon of the *HvMPK6* gene, covering 117 bp of the 5′ untranslated region (5′ UTR) and the first 79 bp of the coding sequence, is missing in the sequence of HORVU7Hr1G023760, suggesting incompleteness of this gene model. Furthermore, we identified and compared all transcripts associated with the *HvMPK6* gene, which are available *in silico* (Supplementary Figure 2). HORVU7Hr1G023760 codes four different splicing variants HORVU7Hr1G023760.1-60.4, among which HORVU7Hr1G023760.2 is the longest one and except for the missing first 196 bp and the last 7 bp 100% covers the AK376245 reference transcript with the 100% nucleotide identity. Two *HvMPK6* transcripts, BART1_0-u50115.001 and BART1_0-u50115.002, were identified in the BaRTv1.0 reference barley transcript data set (Supplementary Figure 2). The first one corresponds to the AK376245 transcript, except for the missing first 196 bp. The second one is missing the first 217 bp of AK376245 and, as a result of alternative selection of the 3′ splice site within the second exon, it is also missing the first 19 bp of the second exon of AK376245.

### Generation and Initial Selection of the Barley Plants Mutated in the *HvMPK6* Gene

Using three independent CRISPR/Cas9 target-designing tools, we selected a single-on-target region in a distal part of the third exon of the *HvMPK6* gene (Figures 1A,B). The position of the designed on-target allows for the disruption of the coding region of all known transcript variants associated with the *HvMPK6* gene, except for HORVU7Hr1G023760.4, which, however, codes only for a short peptide (33 aa) (Supplementary Figure 2). Based on the selected on-target, we prepared a CRISPR/Cas9 construct, which was designated as CC9-K6E3 (Figure 1C).

**Figure 2:**
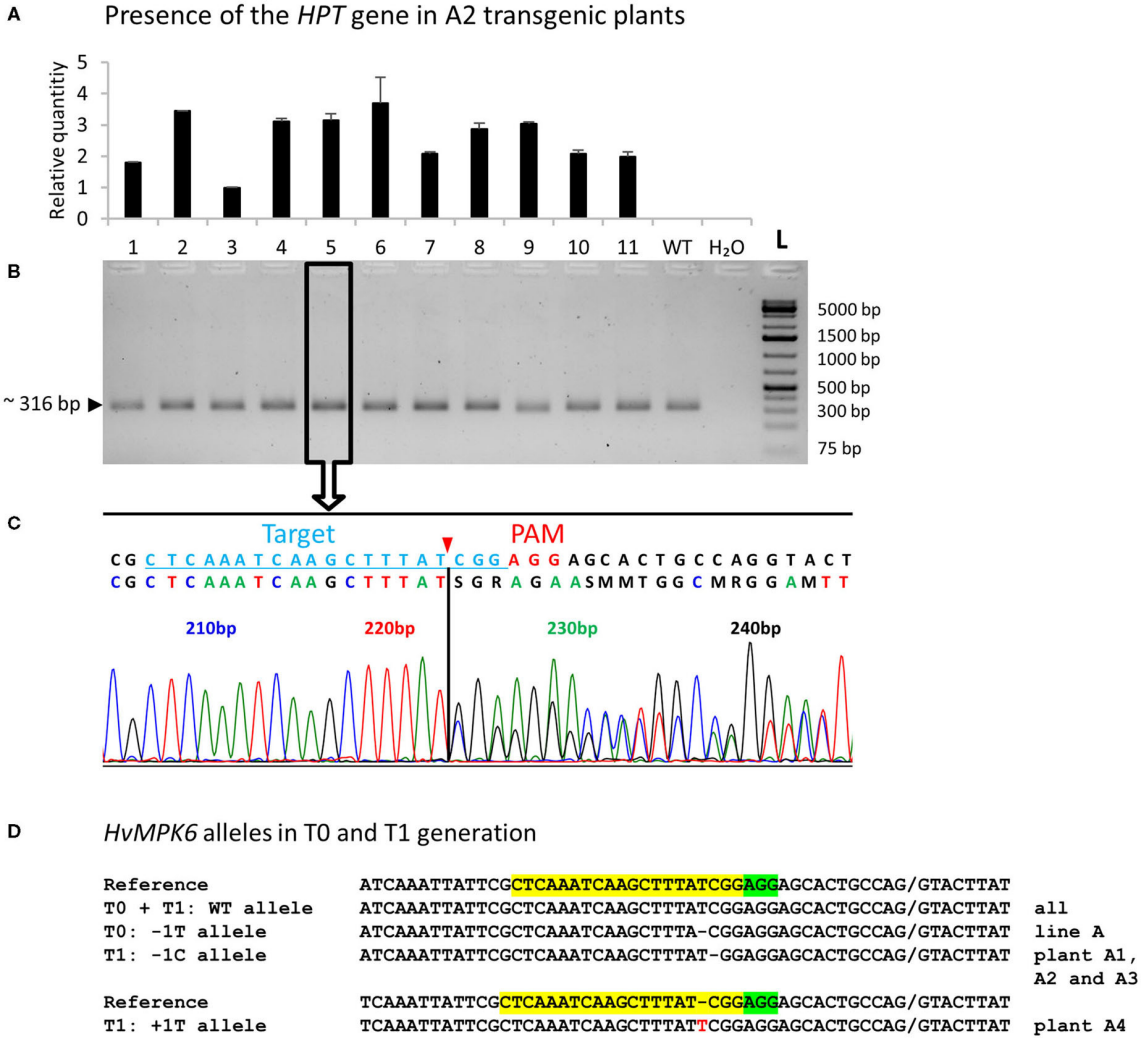
Genotyping of the transgenic barley plants by multiplex TaqMan qPCR and on-target region sequencing—representative results and decoding of the *HvMPK6* alleles of T0 and T1 plants. **(A)** Genotyping of T2-generation plants for the presence of the CC9-K6E3 construct. Genomic DNA was extracted from the leaves of 11 transgenic plants (numbered 1–11) and 1 wild-type (WT) plant and analyzed for the presence of the *HPT* gene by multiplex TaqMan qPCR with primers/probe sets for the *HPT* gene and barley reference *CO2* gene (Supplementary Table 1). Using the 2^−ΔΔCt^ method (Livak and Schmittgen, 2001), the amount of the *HPT* gene was normalized to the amount of the *CO2* gene and is shown for each sample as relative to the sample from plant 3. All the analyzed transgenic plants contained a *HPT* gene whereas WT plant and a negative water-template control (H_2_0) did not. **(B)** The same plants were genotyped for the gRNA:Cas9-induced on-target mutations by PCR sequencing (PCR-Seq). The DNA fragment covering the on-target region was amplified from the genomic DNA of the analyzed plants by using PCR with primers K6E3_F1/K6E3_R1 (Supplementary Table 1). About 316-bp PCR amplicon was associated with all analyzed plants including WT plant but not with a negative water-template control (H_2_O) L- O'GeneRuler 1 kb Plus DNA Ladder (Thermo Fisher Scientific, Waltham, MA, USA). The expected size of the non-mutated PCR product is shown on the left, and the sizes of the selected ladder fragments are shown on the right. All about 316-bp PCR amplicons were sequenced. Open rectangle with an arrow indicates about 316-bp PCR amplicon associated with the plant 5 for which sequencing results are shown below. **(C)** Two overlapping sequencing chromatograms of similar signal intensity indicate heterozygous (monoallelic) or biallelic mutation in the *HvMPK6* gene. Above the chromatograms, a deduced color-coded nucleotide sequence and reference WT nucleotide sequence including the position of the on-target site (underlined light blue typing) and the PAM site (red typing) are shown. The nucleotide numbering is shown as the distance in base pairs (bp) from the first nucleotide of the K6E3_F1 primer site. The deduced sequence corresponds to the WT sequence up to the third nucleotide position upstream of the PAM site (indicated by a vertical solid line) at which peaks start to overlap. Cas9 endonuclease from *Streptococcus pyogenes* cleaves target DNA sequences most frequently after the third nucleotide upstream of a PAM site (Jinek et al., 2012; Ran et al., 2015) (red arrowhead). The vertical solid line and red arrowhead point to the same position suggesting that the on-target was cleaved at the expected site and that the mutation/mutations occurred next to the cleavage site in the downstream direction relative to the PAM site. **(D)** *HvMPK6* alleles of the mutated T0 and T1 transgenic plants. Superimposed sequencing chromatograms were decoded into allelic sequences by using the DSDecodeM tool (Liu et al., 2015b). The selected region of the WT *HvMPK6* allele (plus strand) is shown as a reference on the top of each multiple sequence alignment with the gRNA on-target and PAM site highlighted in yellow and green, respectively. Slash indicates an exon/intron boundary and dash indicates single-nucleotide deletion. An inserted nucleotide is typed in red. The designation of an allele and respective transgenic generation is shown on the left and the designation of the line/plant is shown on the right of each sequence. Minus indicates deletion, plus indicates insertion, and bp indicates base pair.

To generate transgenic barley plants with the edited *HvMPK6* gene, we used two different protocols based on *A. tumefaciens*-mediated transformation of immature barley embryos (Harwood, 2014; Marthe et al., 2015). Following the method of Harwood (2014) with modifications in hygromycin concentration (see Section Development of Transgenic Barley Lines), we transformed 402 immature embryos with the CC9-K6E3 construct and obtained 60 independent plant lines represented by 86 plants in the T0 generation (Table 1). These 86 plants were genotyped for the presence of the CC9-K6E3 cassette by the TaqMan Multiplex qPCR with primers and probe sets designed for the detection of the *HPT* and *CO2* genes (Figure 1C and Supplementary Table 1). CC9-K6E3 transfer DNA (T-DNA) was detected in the genomes of seven plants, representing five independent transgenic lines (Figure 2A and Table 1). Using the method of Marthe et al. (2015), we transformed 330 immature embryos and obtained 10 independent plant lines represented by 10 plants in T0 generation (Table 1). CC9-K6E3 T-DNA was detected in the genomes of eight out of 10 independent lines (Figure 2A and Table 1). In summary, 13 independent transgenic barley lines carrying the CC9-K6E3 construct in the genome were obtained in T0 generation (Table 1). To identify gRNA:Cas9-induced mutations, a 316-bp DNA fragment covering the on-target region of the *HvMPK6* gene was amplified from the genomic DNA of transgenic plants by using PCR with the primers K6E3_F1/K6E3_R1 (Figures 1B, 2B and Supplementary Table 1). Sanger sequencing of the obtained about 316-bp amplicons led to the detection of the induced mutation in the on-target genomic region of one of the 13 analyzed transgenic lines (Figure 2C and Table 1). Two superimposed sequencing chromatograms of the similar signal intensity, which were associated with the mutated line, indicated a heterozygous (WT/single mutation) or biallelic (two distinct variants) nature of the induced mutation (Figure 2C). Decoding of the superimposed chromatograms using the DSDecodeM tool revealed that the respective transgenic line, designated as line A, was a putative heterozygote of a WT *HvMPK6* allele and a mutant *Hvmpk6* allele (Figure 2D). The mutant allele harbored single base pair thymine-adenine (TA) deletion in the fourth nucleotide position upstream of the PAM site (further designated as the −1T allele).

**Table 1:** Overview of the transformation experiments, T0 generation recovery, and genotyping results.

**Method**	**Round of transformation**	**Transformed embryos**	**Plant lines recovered**	**Plants recovered**	**Independent T-DNA lines**	**Plants carrying T-DNA**	***HvMPK6*-mutated lines**
Harwood (2014)	1	107	21	26	1	1	1
	2	95	30	39	4	6	0
	3	200	9	21	0	0	0
**Total**	**3**	**402**	**60**	**86**	**5**	**7**	**1** ^a^
Marthe et al. (2015)	1	180	3	3	2	2	0
	2	150	7	7	6	6	0
**Total**	**2**	**330**	**10**	**10**	**8**	**8**	**0**
**Total both methods**	**5**	**732**	**70**	**70**	**13**	**15**	**1**

a *Mutated transgenic line was further designated as line A*.

Next, we genotyped the T1 progeny plants of line A for the presence of CC9-K6E3 T-DNA and induced mutations in the on-target region of the *HvMPK6* gene as described above. Among 37 analyzed plants, 36 contained T-DNA; however, only six plants were carrying mutation in the on-target region of the *HvMPK6* gene (**Table 3**). Four mutated plants were each associated with two overlapping sequencing chromatograms of the similar signal intensity (Figure 2C), and two mutated plants were each associated with two overlapping chromatograms of different signal intensity suggesting chimerism (Supplementary Figure 3A). Decoding of the chromatograms by DSDecodeM indicated that the first four plants were heterozygous mutants and three of these plants, designated as A1, A2, and A3, carried a single base pair CG deletion in the third nucleotide position upstream of the PAM site in the mutated allele (−1C) (Figure 2D). The remaining putative heterozygous plant designated as A4 carried a single base pair insertion TA between the third and fourth nucleotide position upstream of the PAM site in the mutated allele (+1T) (Figure 2D). Partial decoding of the respective superimposed chromatograms suggested that the last two plants were chimeras comprised more of WT cells than of mutated cells (Supplementary Figure 3A). The number of the mutated T1 plants (six out of 37 analyzed) was very low. Also, all four putative T1 heterozygotes carried the mutated allele (either −1C or +1T) different from the mutated allele of their T0 parent (−1T). Therefore, the T0 parental plant of line A was most probably a chimera comprised more of WT cells than of mutated cells. Originally, T0 parental plant was identified by a sequence analysis of the associated about 316-bp PCR amplicon as a heterozygote (see above) possibly because its non-chimeric heterozygous leaf segment was collected for the mutation analysis.

### Seedling Emergence Percentage Is Substantially Decreased in T2 and T3 Progeny of Heterozygous Mutants in the *HvMPK6* Gene (WT/−1C)

**Figure 3:**
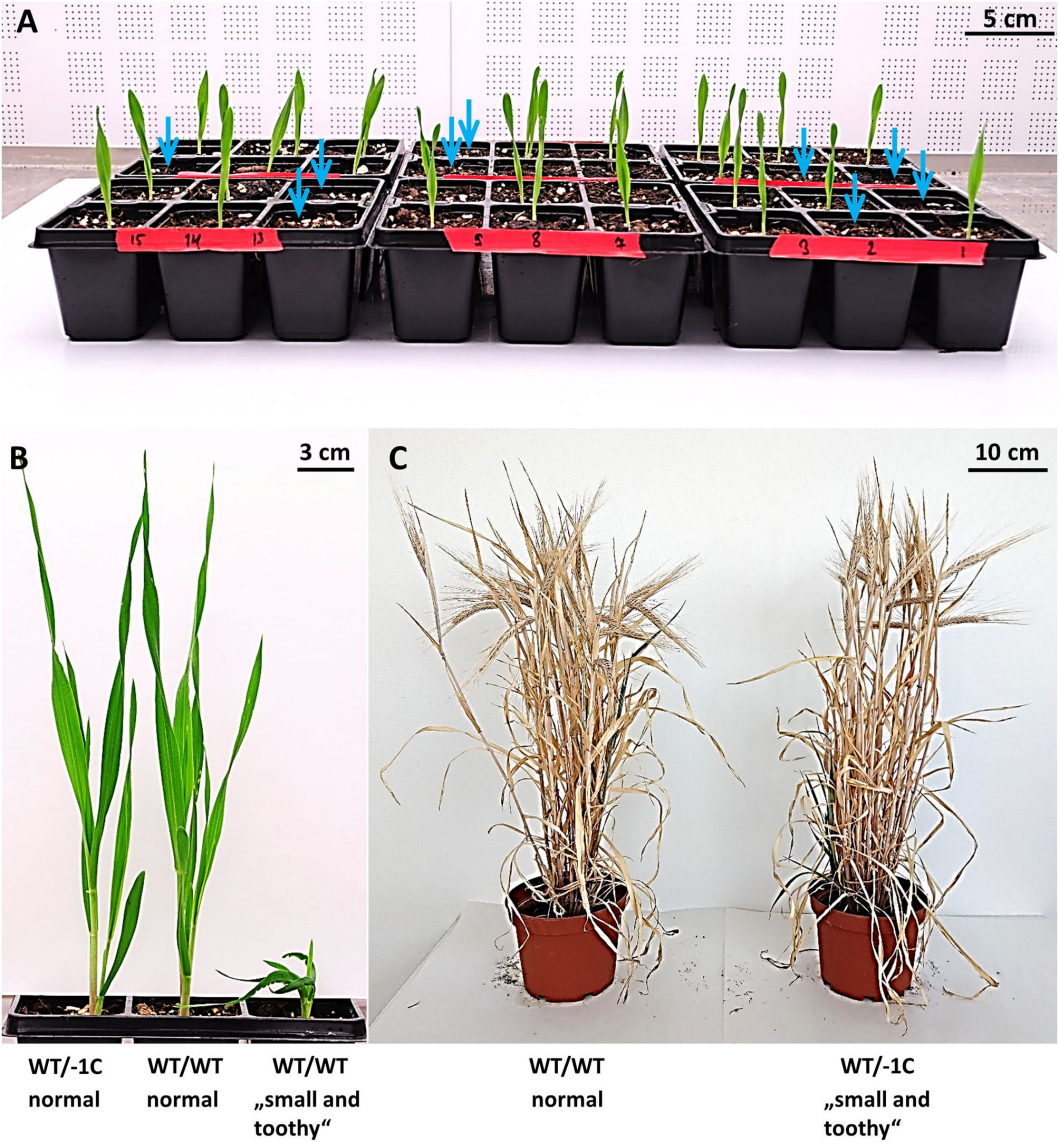
Seedling emergence and phenotypes of emerged plants in the progeny of heterozygous mutants in the *HvMPK6* gene (WT/−1C). **(A)** Seedling emergence of the T3 progeny of A1 heterozygous mutant (WT/−1C). A single grain was sown in each pot of the planter. Seedlings were 7 days old when photographed. Light blue arrows indicate pots with no plant emergence. **(B)** A 4 weeks old normal heterozygous mutant (WT/−1C), normal WT (WT/WT), and rare “small and toothy” (WT/WT) transgenic plant. T3 progeny plants of A2 heterozygous mutant (WT/−1C) are shown. There were no obvious macroscopical phenotype differences between normal heterozygous mutant and normal WT transgenic plants. The rare “small and toothy” phenotype was associated with both heterozygous mutant and WT transgenic plants. **(C)** When transferred to a larger pot, “small and toothy” plants fully recovered during ontogenesis. The images of T2-generation transgenic mature plants are shown. The plant on the left is a normal WT (WT/WT) transgenic plant and the plant on the right is a heterozygous mutant (WT/−1C), which displayed the “small and toothy” phenotype at the seedling stage.

Concerning the chimeric nature of the T0 parental plant A, we handled putatively heterozygous T1 plants A1 (WT/−1C), A2 (WT/−1C), A3 (WT/−1C), and A4 (WT/+1T) as individual sublines of line A and characterized their T2 progeny separately. Since immature barley embryo culture may produce polyploid regenerants, we initially confirmed that T2 progeny of line A was diploid as its donor GP cultivar (Supplementary Figure 4). We observed substantially decreased seedling emergence percentage (SEP) in the T2 progeny of A1, A2, and A3; in particular, it was 67.78% (61 emerged seedlings/90 grains sown), 67.78% (61/90), and 61.43% (43/70) in A1, A2, and A3, respectively (Table 2). Contrary to this observation, SEP of A4 and WT GP control was 100% (70/70) and 96.67% (87/90), respectively (Table 2). Mutation analysis of the emerged T2-generation plants confirmed that parental T1-generation plants A1, A2, and A3 were all heterozygotes of the WT *HvMPK6* allele and mutant −1C *Hvmpk6* allele (WT/−1C) whereas A4 was a chimera composed predominantly of WT cells (see below). A low portion of mutated cells of A4 plant is most probably responsible for 100% SEP of its T2 progeny. In the T3 generation, SEP was also substantially decreased in the progeny of heterozygous T2-generation plants (WT/−1C), suggesting that decreased SEP is a heritable property. Namely, SEP of the A1 plants was 74.80% (178/238) and SEP of the A2 plants was 75.79% (72/95) whereas SEP of WT GP control was 100% (90/90) (Table 2). In both T2 and T3 generations, transgenic heterozygous mutant and WT seedlings emerged uniformly (Figure 3A). There were no obvious phenotype differences between normal heterozygous mutant (WT/−1C) and normal WT transgenic plants (Figure 3B). Rarely, plants with impaired development were observed in the progeny of WT/−1C plants in both T2 and T3 generations (Figure 3B). In the seedling stage, these plants were small with sickle and toothy leaves. However, when transferred to a larger pot, they fully recovered (Figure 3C). The frequency of the “small and toothy” plants was similar in the T2 and T3 generation. In the T2 generation, it was 4.36% (13 plants observed/ 298 grains of A1, A2, and A3 sown in total) and in the T3 generation, it was 3.90% (13/333 grains of A1 and A2). We never observed such “small and toothy” plants among control WT seedlings (180 grains sown in total).

**Table 2:** Seedling emergence of the T1, T2, and T3 progeny of the line A.

**Transgenic generation**	**Parental genotype identity**	**Grains sown**	**Emerged plants**	**No emergence**	**SEP %**	**SREN**	**χ^**2**^ value (3:1)**	***p*-value**
T1	A^a^ (WT/-1T)	52	49	3	94.23	n.d.	n.d.	n.d.
T2	A1 (WT/-1C)	90	61	29	67.78	2.10:1	2.50	>0.10 n.s.
	A2 (WT/-1C)	90	61	29	67.78	2.10:1	2.50	>0.10 n.s.
	A3 (WT/-1C)	70	43	27	61.43	1.59:1	6.88	<0.01 s.
	A4^a^ (WT/+1T)	70	70	0	100	n.d.	n.d.	n.d.
	WT	90	87	3	96.67	n.d.	n.d.	n.d.
**Total T2**	**A1+A2+A3**	**250**	**165**	**85**	**66**	**1.94:1**	**10.80**	**<0.01 s**.
T3	A1 (WT/-1C)	238	178	60	74.80	2.97:1	0.0056	>0.94 n.s.
	A2 (WT/-1C)	95	72	23	75.79	3.13:1	0.0316	>0.85 n.s.
	WT	90	90	0	100	n.d.	n.d.	n.d.
**Total T3**	**A1+A2**	**333**	**250**	**83**	**75.08**	**3.01:1**	**0.0010**	**>0.97 n.s**.

a *These plants were chimeras composed more of WT cells than of mutated cells*.

*SEP, seedling emergence percentage, SREN, segregation ratio of the emerged plants to non-emerged. n.d., not determined*.

Decreased SEP in the progeny of T1 and T2 heterozygous plants (WT/−1C) suggested lethality of the −1C/−1C homozygous knockout mutation in the *HvMPK6* gene. Therefore, we verified the segregation ratio of the emerged plants to non-emerged ones (SREN) in the progeny of T1 and T2 heterozygous mutants (WT/−1C) by using the χ^2^ test (Table 2). SREN was 2.10:1 (61 emerged plants/29 non-emerged) in the T2 progeny of A1 and fitted the expected Mendelian 3:1 ratio (χ^2^, *p* > 0.10 n.s.). Identically, SREN was 2.10:1 (61/29) in the T2 progeny of A2 and fitted 3:1 ratio (χ^2^, *p* > 0.10 n.s.). In the T2 progeny of A3, SREN was 1.59:1 (43/27) and it did not fit the 3:1 ratio (χ^2^, *p* < 0.01 n.s.). In total, SREN of all three T2 progenies was 1.94:1 (165/85), and it did not fit the 3:1 ratio (χ^2^, *p* < 0.01 n.s.). In the T3 progeny of A1 and A2, SREN was 2.97:1 (178/60) and 3.13:1 (72/23), respectively, and, in both cases, fitted the expected 3:1 ratio (χ^2^, *p* > 0.94 n.s.) and (χ^2^, *p* > 0.85 n.s.), respectively (Table 2). In total, SREN of both two T3 progenies was 3.01:1 (250/83) and fitted the expected 3:1 ratio (χ^2^, *p* > 0.97 n.s.) (Table 2).

### None of the Plants Emerged in T2 and T3 Generation Is a Biallelic Mutant in the *HvMPK6* Gene

**Figure 4:**
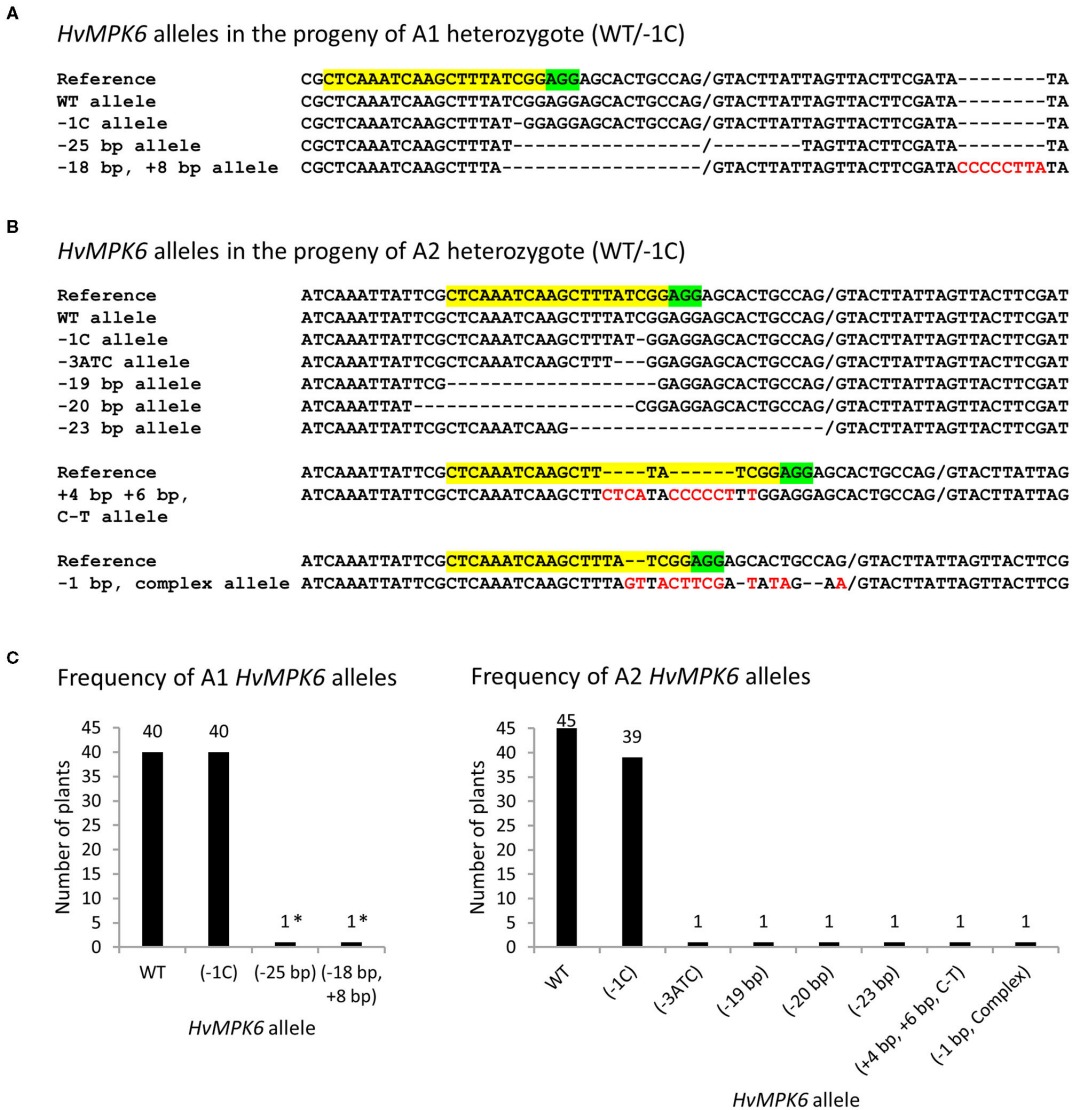
*HvMPK6* alleles of the mutated emerged T2 progeny plants of WT/−1C heterozygotes. Genomic DNA for the mutation analysis was extracted from the leaves of the transgenic plants. Superimposed sequencing chromatograms associated with heterozygous or chimeric plants were decoded into allelic sequences by using the DSDecodeM tool, cloning of about 316-bp PCR amplicons, and occasionally manual decoding. **(A)** Nucleotide sequences of the *HvMPK6* alleles identified in the progeny of A1 heterozygous mutant (WT/−1C). **(B)** Nucleotide sequences of the *HvMPK6* alleles indentified in the progeny of A2 heterozygous mutant (WT/−1C). The selected nucleotide sequence region of the WT *HvMPK6* allele is shown as a reference on the top of each multiple sequence alignment with the gRNA on-target site and the PAM site highlighted in yellow and green, respectively. Slash indicates exon/intron boundary and dash indicates single nucleotide deletion. Inserted or changed nucleotides are typed in red. Designation of an allele is shown on the left of each sequence. Minus indicates deletion, plus indicates insertion, and bp indicates base pair. Note that the −25 bp, −18 bp + 8 bp, and −23 bp mutations eliminated the splicing site between the exon 3 and intron 4. **(C)** Frequency of the *HvMPK6* alleles in the mutated progeny plants of A1 (WT/−1C) and A2 (WT/−1C) heterozygotes. Absolute numbers of individual alleles are plotted and shown above each bar chart. In total, 40 and 45 mutated A1 and A2 plants, respectively, were identified and all of them contained WT allele. Among mutated alleles, the −1C *Hvmpk6* allele was prevalent and others were rare. Nearly all mutated plants were heterozygotes of WT and −1C allele. Two A1 plants were chimeras and harbored −25bp or −18bp, +8bp allele (indicated with asterisks) in addition to the WT and −1C allele.

To get insight into the mechanism explaining a decreased SEP of the progeny of heterozygous mutant plants, we analyzed the plants that emerged in T2 generation for the gRNA:Cas9-induced on-target mutations in the *HvMPK6* gene. Initially, 59, 62, and 43 emerged plants (in total 164 plants) of the T2 progeny of A1 (WT/−1C), A2 (WT/−1C), and A4 (WT/+1T), respectively, were genotyped by sequencing of the associated about 316-bp PCR amplicons (PCR-Seq). Out of 164 plants analyzed, 102 plants were mutated (Table 3). The 96 mutated plants were heterozygotes of the WT *HvMPK6* allele and the mutated *Hvmpk6* allele (Figure 2C), and the remaining six mutated plants were chimeras (Supplementary Figure 3). Mutation frequency in individual T2 progenies of A1 and A2 was similar, with values of 67.80% (40 mutated plants/59 plants analyzed) for the A1 progeny and 72.58% (45/62) for the A2 progeny, but was substantially lower in the T2 progeny of A4 with a value of 39.53% (17/43) (Table 3). The analyzed A1 and A2 offspring plants harbored three and seven different mutated *Hvmpk6* alleles, respectively (Figure 4). All three *Hvmpk6* alleles of A1 plants contained frame-shift mutations (−1C, −25 bp and −18 bp + 8 bp) (Figure 4A). Concerning A2 plants, six *Hvmpk6* alleles harbored frame-shift mutations (−1C, −19 bp, −20 bp, −23 bp, +4 bp +6 bp C-T, and −1 bp complex) and one *Hvmpk6* allele harbored an in-frame mutation (−3ATC) (Figure 4B). The −25 bp, −18 bp + 8 bp, and −23 bp mutations eliminated the splicing site between the exon 3 and intron 4 (Figures 4A,B). When the identified mutations were individually introduced into the nucleotide sequence of the longest *HvMPK6* splicing variant (AK376245.1.mrna1_gpv1_chr7H_26521825_26529501_0) *in silico*, all the respective frameshifts were predicted to result in abnormal severely truncated HvMPK6 proteins (Supplementary Figure 5). Out of 40 mutated A1 plants, 38 were heterozygotes of the WT and −1C allele and 2 plants were multiple genotype chimeras (Supplementary Figure 3B), which, in addition to the WT allele and −1C allele, carried −25 bp and −18 bp +8 bp allele, respectively (Figures 4A,C). Similarly, 43 out of 45 mutated A2 plants were heterozygotes of WT and −1C allele (37 plants) or another *Hvmpk6* allele (six plants), and the remaining two plants were multiple genotype chimeras (Supplementary Figure 3B), which harbored WT allele and −1C allele in addition to the unidentified *Hvmpk6* allele (Figures 4B,C). Apparently, all 38 A1 heterozygous plants and a large majority of A2 heterozygous plants (37 out of 43) carried the same mutated allele (−1C) as their heterozygous T1 parent (WT/−1C), suggesting transmission of the −1C allele from parental generation. Contrary to these observations, 15 putatively heterozygous plants of the 17 mutated A4 plants carried 12 different *Hvmpk6* alleles and none of these alleles was identical to the +1T allele of their heterozygous T1 parent (WT/+1T) (Supplementary Figure 6 and Table 3). The remaining two A4 plants were chimeras composed more of WT cells than of mutated cells (Supplementary Figure 3A). For one of these chimeric plants, we decoded a sequence of the *Hvmpk6* allele manually (Supplementary Figure 6). Taken together, these results suggested that parental T1-generation plants A1 and A2 were heterozygotes (WT/−1C) associated with no or limited chimerism whereas parental T1-generation plant A4 was a chimera composed more of WT cells than the cells of mutated cell lines.

**Table 3:** Detection of the on-target mutations and T-DNA in emerged plants in T1, T2, and T3 generation.

**Transgenic generation**	**Parental genotype**	**Plants analyzed**	**Plants carrying T-DNA**	**Plants mutated in *HvMPK6***	**WT**	**Mutation frequency %**	**Biallelic *Hvmpk6* mutants**
**T1**	A^a^ (WT/-1T)	37	36	6 (3/3^a^^d^)	31	16.22	0
**T2**	A1 (WT/-1C)	59	59	40 (38/2^b^)	19	67.80	0
	A2 (WT/-1C)	62	62	45 (43/2^b^)	17	72.58	0
	A3 (WT/-1C)	24	24	17^c^	7	70.83	0
	A4^a^ (WT/+1T)	43	43	17 (15/2^a^)	26	39.53	0
**Total sequencing**	**A1+A2+A4**	**164**	**164**	**102**	**62**	**62.20**	**0**
**Total (WT/-1C)**	**A1+A2+A3**	**145**	**145**	**102**	**43**	**70.35**	**0**
**Total T2**	**All**	**188**	**188**	**119**	**69**	**63.30**	**0**
**T3**	A1 (WT/-1C)	40	40	27^c^	13	67.50	0
	A2 (WT/-1C)	20	20	13^c^	7	65.00	0
**Total T3**	**WT/-1C**	**60**	**60**	**40** ^**c**^	**20**	**66.67**	**0**
**Total (WT/-1C) T2+T3**	**A1+A2+A3**	**205**	**205**	**142**	**63**	**69.27**	**0**
**Total T2+T3**	**All**	**248**	**248**	**159**	**89**	**64.11**	**0**

*In the column “Plants mutated in HvMPK6,” there are numbers in brackets. The number before slash indicates the number of heterozygotes of the WT HvMPK6 allele and mutant Hvmpk6 allele, the number after slash indicates the number of chimeras*.

a *These plants were chimeras composed more of WT cells than of mutated cells*.

b *These plants were multiple genotype chimeras of the WT HvMPK6 allele and two different mutated Hvmpk6 alleles*.

c *These plants were heterozygotes of the WT HvMPK6 allele and mutant Hvmpk6 allele*.

d *These three chimeras include also a chimeric A4 plant, which, in T1 generation, was identified as a putative heterozygous mutant in the HvMPK6 gene (WT/+1T)*.

**Figure 5:**
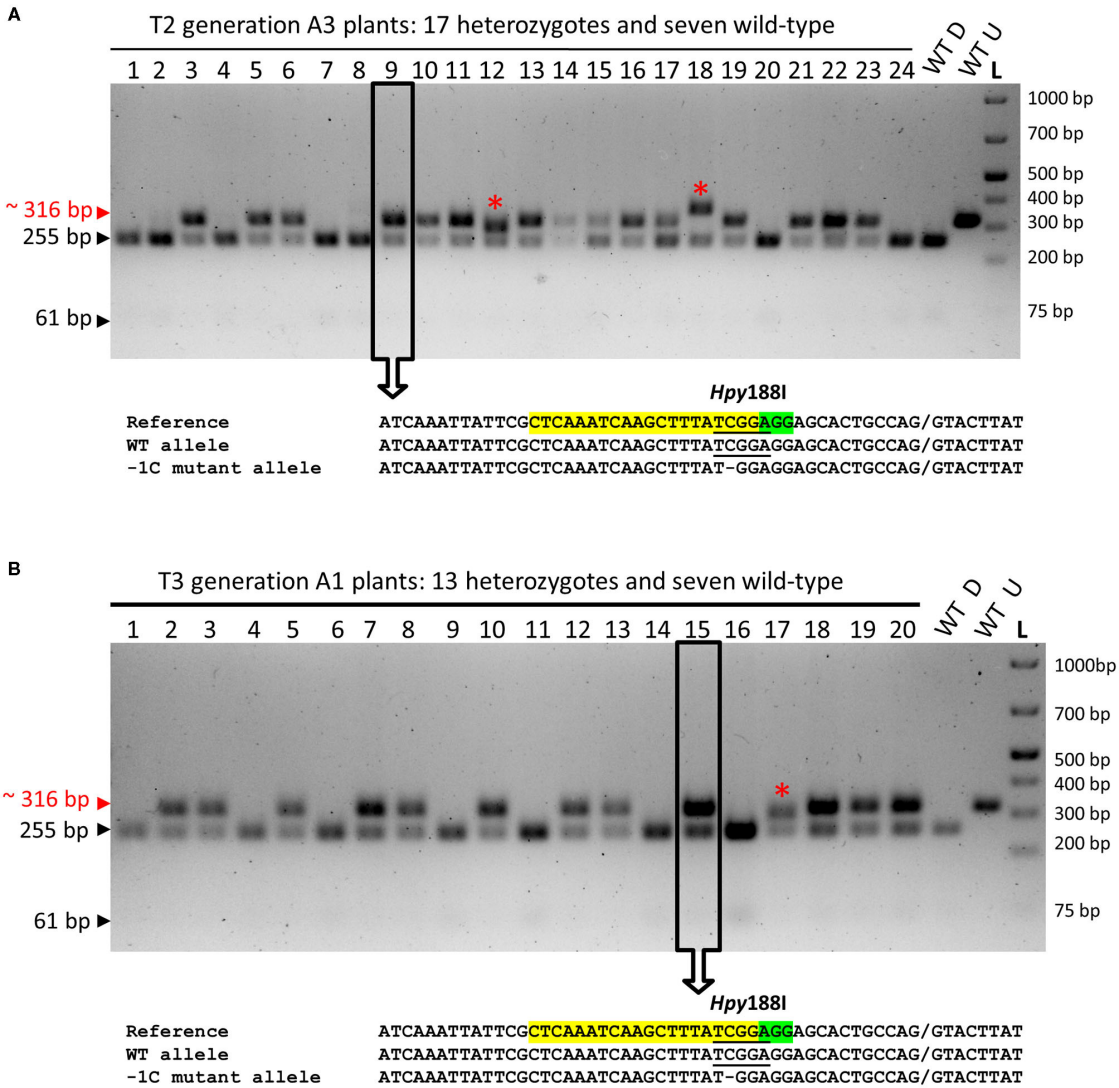
PCR-RE genotyping of the emerged T2- and T3-generation plants for the on-target mutations in the *HvMPK6* gene. **(A)** PCR-RE genotyping of 24 (1–24) T2 progeny plants of A3 heterozygous mutant (WT/−1C). **(B)** Representative results of PCR-RE genotyping of T3 progeny plants of A1 and A2 heterozygous mutants (WT/−1C). In particular, the results of PCR-RE genotyping of 20 (1–20) T3 progeny plants of A1 heterozygous mutant (WT/−1C) are shown. Genomic DNA for the mutation analysis was extracted from the leaves of the transgenic plants. About 316-bp PCR amplicons of the genomic region covering the on-target site were digested with *Hpy*188I. The red arrowhead and red typing of the size of the PCR product indicate undigested PCR amplicons containing mutation which disrupt the *Hpy*188I recognition site. Black arrowheads and black typing of the sizes of the PCR products indicate the digested WT PCR amplicons. Wt D—digested and Wt U—undigested reference 316-bp amplicons associated with WT plant. L-O'GeneRuler 1 kb Plus DNA Ladder (Thermo Fisher Scientific, Waltham, MA, USA). Sizes of the selected ladder fragments are shown on the right. None of the analyzed plants is a homozygous mutant. The asterisk indicates a rare heterozygous restriction pattern. Several PCR products associated with a common heterozygous restriction pattern (examples in open rectangles with an arrow) were sequenced, and the resulting superimposed chromatograms were decoded into allelic nucleotide sequences (bellow electrophoreograms) with the DSDecodeM tool (Liu et al., 2015b). The selected nucleotide sequence region of the WT *HvMPK6* allele is shown as a reference on the top of each multiple sequence alignment with the gRNA on-target site and the PAM site highlighted in yellow and green, respectively. The designation of an allele is shown on the right. Slash indicates an exon/intron boundary and dash indicates a single-nucleotide deletion. Where present, the *Hpy*188I recognition site is underlined. Mutated allele associated with the common heterozygous restriction pattern contains a single GC deletion at the third nucleotide position upstream of the PAM site (−1C allele).

Also, we genotyped 24 emerged plants of the T2 progeny of A3 plant (WT/−1C) by using restriction digestion of the associated about 316-bp PCR amplicons with *Hpy*188I (PCR-RE) (Figures 1B, 5A). *Hpy*188I is suitable for the detection of the −1C mutation, which occurs in the third nucleotide position upstream of the PAM site (Figure 1B). Among the 24 analyzed A3 plants, 17 plants were heterozygous mutants and seven were WT, providing a mutation frequency of 70.83% (17/24), which was similar to the frequency of the mutated A1 (67.80%) and A2 (72.58%) plants in the T2 generation (Figure 5A and Table 3). Sequencing and decoding of amplicons associated with five heterozygotes and a common restriction fragment pattern (further designated as a “WT/−1C” pattern), confirmed that these were heterozygotes of WT and −1C allele (Figure 5A). There were in total 15 heterozygotes associated with the “WT/−1C” pattern, and only two heterozygotes were associated with an unusual restriction fragment pattern (Figure 5A). Thus, 15 out of 17 heterozygotes were most probably comprised of WT and −1C allele and carried the same mutated allele (−1C) as their T1 parent (WT/−1C). In conclusion, identically to the T1-generation plants A1 and A2, parental T1-generation plant A3 was a heterozygote (WT/−1C) associated with no or limited chimerism.

In total, 119 mutated plants (A1 + A2 + A3 + A4) were identified by PCR-Seq and PCR-RE among 188 analyzed emerged plants in T2 generation (Figures 4, 5A and Table 3). None of these plants was a biallelic mutant in the *HvMPK6* gene and all contained WT *HvMPK6* allele. Also, nearly all mutated emerged progeny plants of A1, A2, and A3 heterozygotes (WT/−1C) carried the −1C allele (Figures 4, 5A). Taken together, these data strongly suggested that −1C/−1C homozygous knockout mutation in the *HvMPK6* gene is lethal.

To further verify the inheritance of the −1C allele and its phenotypic effects in T3 generation, we genotyped the emerged T3 progeny plants of the T2 heterozygous A1 (WT/−1C) and A2 (WT/−1C) parents using PCR-RE (Figure 5B and Table 3). Out of the 40 analyzed A1 plants, 27 were heterozygotes in the *HvMPK6* gene and 13 were WT, providing a mutation frequency of 67.5% (27/40). Similarly, 13 out of 20 analyzed A2 plants were heterozygotes and seven were WT, providing a mutation frequency of 65% (13/20). The overall mutation frequency of the analyzed T3-generation plants was 66.67% (40/60), and 37 of 40 heterozygotes were associated with a “WT/−1C” restriction fragment pattern, further confirmed by sequencing and decoding of several respective about 316-bp PCR amplicons (Figure 5B and Table 3). None of the 40 mutated plants was a biallelic mutant in the *HvMPK6* gene. In conclusion, the data collected on the emerged T3 progeny plants of T2 heterozygotes (WT/-1C) were similar to the data collected on the emerged T2 progeny plants of T1 heterozygotes (WT/-1C) and confirmed the inheritance of the −1C allele from T2 generation to T3 generation as well as the lethality of the −1C/−1C homozygous knockout mutation in the *HvMPK6* gene.

Among the 188 emerged plants in the T2 generation and the 60 emerged plants in the T3 generation, which were genotyped for the on-target mutations (Table 3), we also genotyped 19 “small and toothy” plants (Figure 3B). The overall frequency of the mutated “small and toothy” plants was 84.21% (16 mutated/19 analyzed) and it was higher than the overall mutation frequency of the normal emerged progeny plants of WT/−1C heterozygotes, which was 67.74% (126 mutated/186 analyzed). In total, 12 out of the 16 mutated “small and toothy” plants were heterozygotes of the WT *HvMPK6* allele and a knockout *Hvmpk6* allele and 4 remaining plants were T2-generation chimeras of a WT allele and 2 different mutated alleles (Supplementary Figure 3B).

The presence of the CC9-K6E3 T-DNA was confirmed in the genomes of all 188 genotyped emerged T2-generation plants as well as in the genomes of all 60 genotyped emerged T3-generation plants (Table 3).

### The Minority of the *Hvmpk6* Mutant Grains Develop Into Abnormal Seedlings With Shootless Phenotype and the Majority of Them Do Not Germinate

**Figure 6:**
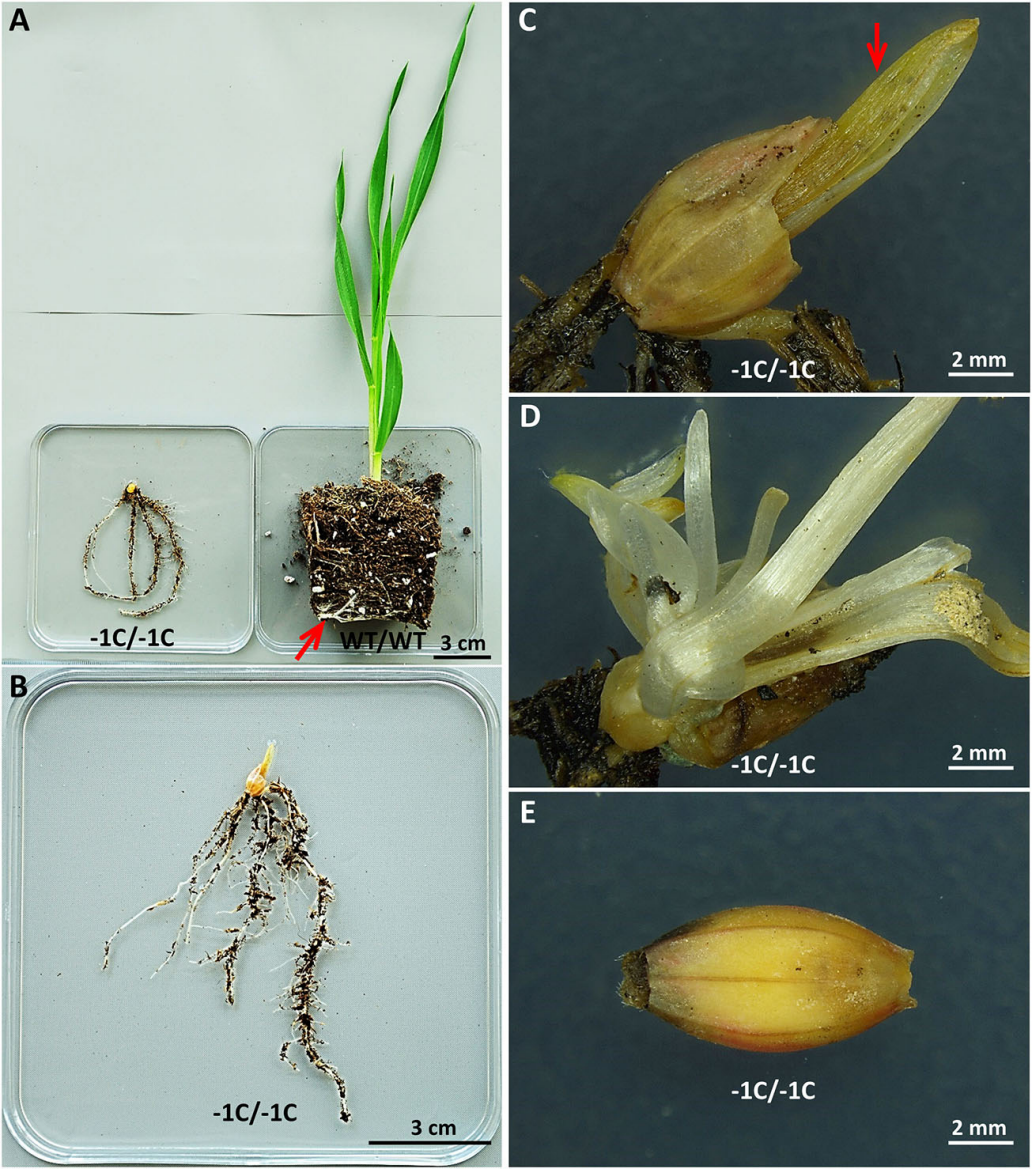
Aberrant phenotypes of the *Hvmpk6* knockout mutant (−1C/−1C) seedlings and grains. **(A)** A mutant (−1C/−1C) seedling and a control WT seedling (WT/WT). The mutant is shootless and has a short root system. Note that the root system of WT is longer than that of the mutant since some of the roots of WT were circling inside the pot (indicated with a red arrow). **(B)** A detailed view of the whole −1C/−1C seedling. **(C)** A detailed view of the grain husk leftover and a small chlorotic leaf blade-like structure (red arrow) of the same seedling. **(D)** A detailed view of multiple small leaf blade-like structures of another −1C/−1C seedling. **(E)** The majority of the −1C/−1C grains did not germinate in the soil. Mutant and control WT seedlings and mutant grains were removed from the soil 3 weeks after sowing and immediately imaged.

We further analyzed the developmental status of the grains in the pots of plastic planters with no plant emergence (Figure 3A). In total, there were 83 pots with no plant emergence in the T3 progeny of the T2 generation A1 (WT/−1C) and A2 (WT/−1C) plants (Table 2), and these were examined 3 weeks after sowing. We found 28 abnormal seedlings and 55 grains in the soil of 83 pots (Figure 6, Supplementary Figures 7, 8, and Table 4). Soil grown homozygous *Hvmpk6* knockout mutant (−1C/−1C) seedlings displayed shootless phenotype but relatively often had a well developed root system, although it was substantially reduced in size as compared to the root system of the control plants (Figures 6A,B). Some of the −1C/−1C seedlings had a more severely reduced root system with decreased size and/or number of roots. Also, some of the −1C/−1C seedlings developed one (Figures 6B,C) or more (Figure 6D) small chlorotic leaf blade-like structures, which did not reach the soil surface and were in some cases longitudinally bent, transversally bent, sickle-shaped, branched, or otherwise deformed. Occasionally, we identified abnormal heterozygous mutant (WT/−1C and in one case also WT/*Hvmpk6* with the mutant allele different from −1C) or WT (WT/WT) seedlings, which did not emerge from the soil (Table 4). Contrary to the −1C/−1C seedlings, five out of the seven identified abnormal heterozygous mutant/WT seedlings developed an aberrant but apparent shoot, which failed to emerge (Supplementary Figure 7). These observations suggested that the absence of shoot in the *Hvmpk6* mutant (−1C/−1C) seedlings was caused by the early defect in the shoot development and/or growth. One of the abnormal WT/−1C seedlings showed some temporary signs of shoot emergence (Supplementary Figure 8). Except for two grains, none of the 55 grains which were found in the soil germinated (Figure 6E).

**Table 4:** Characterization of the plant material removed from the soil of pots with no plant emergence.

**Transgenic generation**	**Parental genotype**	**Material removed**	**Total**	**Genotyped**	**Biallelic *Hvmpk6* mutant**	**Heterozygous mutant in *HvMPK6***	**WT**
T3	A1, A2 (WT/-1C)	seedlings	28	27	20	6	1
		grains	55	23	22	1	0
**Total**			**83**	**50**	**42**	**7**	**1**

**Figure 7:**
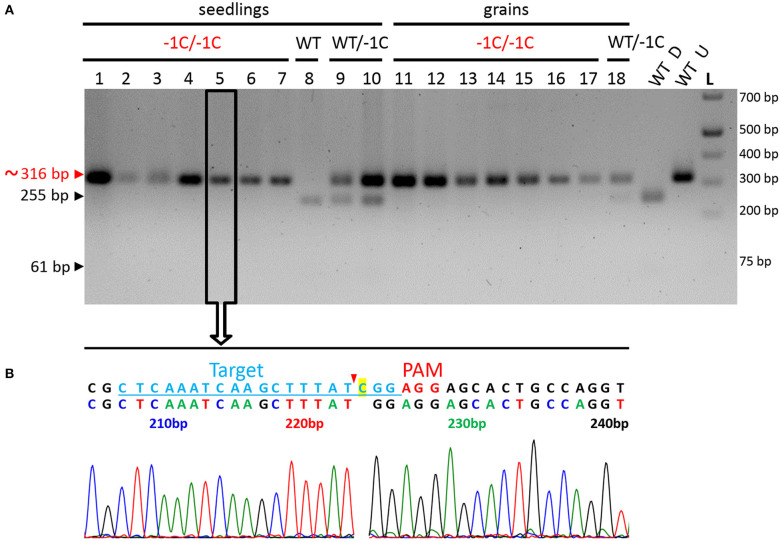
Genotyping of the seedlings and grains removed from the soil for the on-target mutations in the *HvMPK6* gene-representative results. **(A)** PCR-RE genotyping. Seedlings and grains were removed from the soil 3 weeks after sowing. Genomic DNA for the analysis was extracted from the small leaf blade-like structure/structures, seeds' leftovers without husk and roots of seedlings and from the grains without husk. About 316-bp PCR amplicons of the genomic region covering the on-target site, which were associated with 10 seedlings (1–10), and eight grains (11–18) were digested with *Hpy*188I. The red arrowhead and red typing of the size of the PCR product indicate the undigested PCR amplicons contaning mutation which disrupts the *Hpy*188I recognition site. The black arrowheads and black typing of the sizes of the PCR products indicate the digested WT PCR amplicons. Wt D—digested and Wt U—undigested reference 316-bp amplicons associated with a WT plant. L-O'GeneRuler 1 kb Plus DNA Ladder (Thermo Fisher Scientific, Waltham, MA, USA). Sizes of the selected ladder fragments are shown on the right. Seven seedlings and seven grains were homozygous knockout mutants in the *HvMPK6* gene (−1C/−1C). One seedling was WT (WT/WT), two were heterozygotes (WT/−1C), and one grain was a heterozygote (WT/−1C). All 18 PCR amplicons were also sequenced (an example for a −1C/−1C mutant in an open rectangle with an arrow). **(B)** Single sequencing chromatogram associated with the −1C/−1C seedling. Above the chromatogram, deduced color-coded nucleotide sequence and reference WT nucleotide sequence including the position of the on-target site (underlined lightly blue typing) and the PAM site (red typing) are shown. The nucleotide numbering is shown as the distance in bp from the first nucleotide of the K6E3_F1 primer site. Cas9 endonuclease from *S. pyogenes* cleaves target DNA sequences most frequently after the third nucleotide upstream of a PAM site (Jinek et al., 2012; Ran et al., 2015) (red arrowhead). Single base pair CG deletion (“C” highlighted in yellow in a reference sequence) occurs next to the cleavage site at the third nucleotide position upstream of the PAM site. Deduced nucleotide sequence corresponds to the WT nucleotide sequence except for the CG deletion.

Among those described 28 seedlings and 55 grains, 27 seedlings and 23 grains were successfully genotyped by using PCR-RE (Figure 7A and Table 4). The results of this analysis showed that 20 of the analyzed seedlings were biallelic *Hvmpk6* mutants, six seedlings were heterozygous mutants in the *HvMPK6* gene, and one seedling was WT. Similarly, 22 grains were biallelic *Hvmpk6* mutants and one grain was a heterozygous mutant in the *HvMPK6* gene. Next, we sequenced about 316-bp PCR amplicons associated with 13 seedlings (10 biallelic mutants, 2 heterozygotes and 1 WT) and 4 biallelic mutant grains. An analysis of the sequencing data confirmed and specified the results of PCR-RE. All 10 analyzed biallelic mutant seedlings were homozygotes of the −1C *Hvmpk6* allele (−1C/−1C genotype) (Figure 7B and Table 4), 2 heterozygous mutant seedlings were heterozygotes of WT *HvMPK6* allele and −1C *Hvmpk6* allele, and 1 seedling was WT. Also, all four analyzed biallelic mutant grains were homozygotes of the −1C *Hvmpk6* allele (−1C/−1C). The remaining 10 biallelic mutant seedlings and 18 grains, which were not analyzed by PCR-Seq, were associated with the −1C/−1C restriction fragment pattern, suggesting that all or most of them were also −1C/−1C homozygotes (Figure 7A and Table 4). Similarly, out of the four remaining heterozygous seedlings, which were not analyzed by PCR-Seq, (Table 4), three were associated with the −1C/WT restriction pattern (Figure 7A) and only one was associated with the unusual restriction fragment pattern.

### *Hvmpk6* Mutant Grains Contain Abnormal Embryos, Which Predetermine Seedling Shootless Phenotype and Severely Reduced Grain Germination

**Figure 8:**
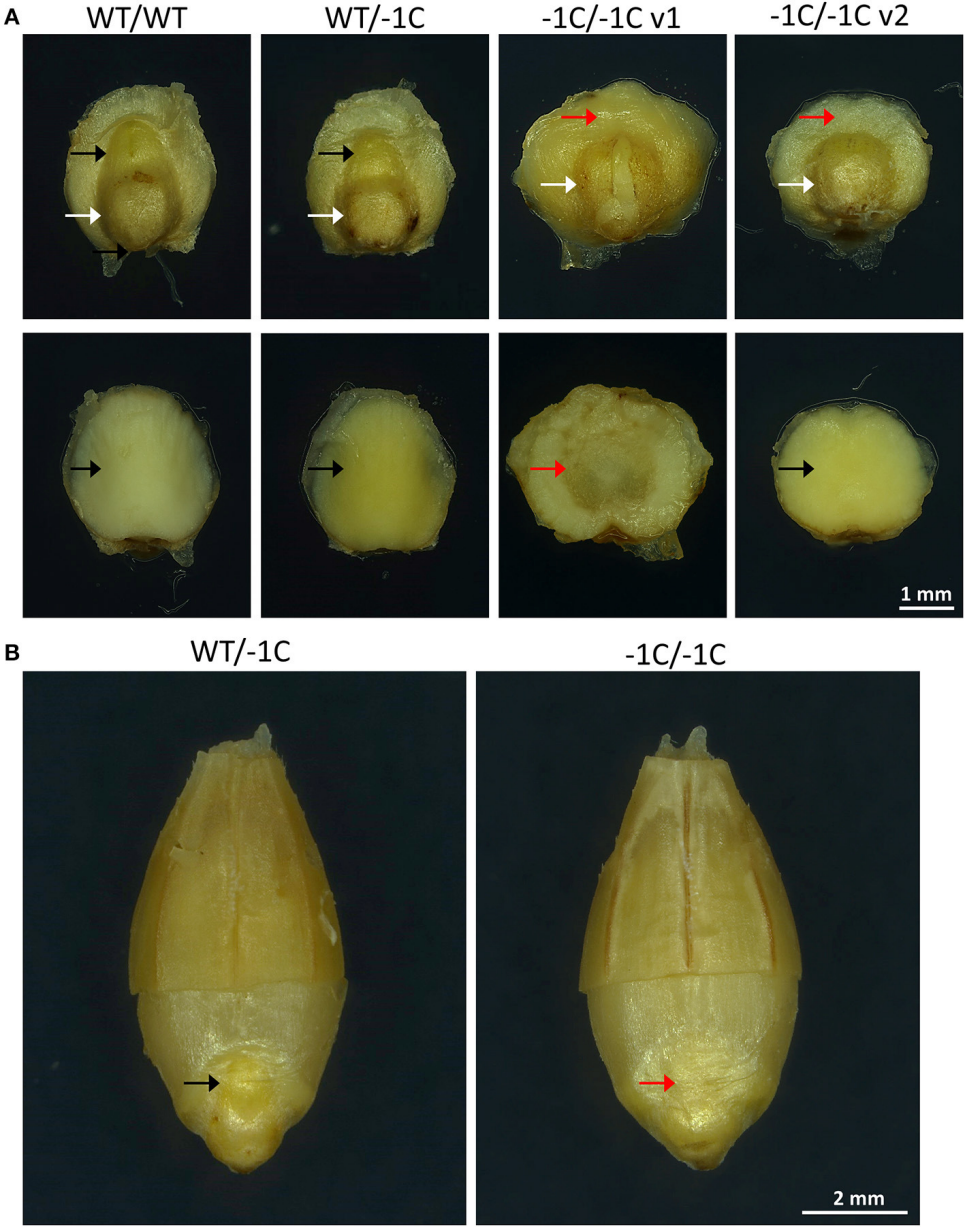
Representative embryos and grains of heterozygous mutants in the *HvMPK6* gene (WT/−1C). T3-generation mature grains of heterozygous transgenic plants and mature grains of WT non-transgenic control plants were imbibed in Milli-Q water at 21°C in darkness for 18 h before embryo extirpation or husk removal and imaging. **(A)** A non-transgenic WT (WT/WT) embryo, a heterozygous mutant (WT/−1C) embryo, and representatives of two types of homozygous knockout mutant embryos (−1C/−1C v1 and −1C/−1C v2). The upper line of images—an adaxial site of the extirpated embryos. The white arrow indicates the root part of the embryonic axis and the black arrow indicates the shoot (upper) part of the axis. The red arrow indicates the missing shoot part of the axis. The lower line of images—an abaxial site of the extirpated embryos. The black arrow indicates the normal shape and the red arrow indicates the hollow shape of the abaxial embryo site. **(B)** A heterozygous mutant (WT/−1C) grain and a homozygous knockout mutant (−1C/−1C) grain. From each grain approximately half of the husk was removed to allow for the embryo morphology evaluation. The black arrow indicates the shoot part of the embryonic axis, and the red arrow indicates the missing shoot part of the axis.

To check if the phenotype of the −1C/−1C seedlings and the absence of the germination of the −1C/−1C grains were predetermined by the abnormally developed embryos, we analyzed the T3-generation grains of the T2-generation heterozygous A1 (WT/−1C) and A2 (WT/−1C) plants. In total, 240 embryos were extirpated from the mature grains of the WT/−1C plants, and in parallel 270 embryos were extirpated from the mature grains of the WT control non-transgenic plants (Table 5). The microscopic analysis revealed that 59 (24.58%) of the 240 embryos of the WT/−1C plants were of abnormal appearance whereas the remaining 181 embryos were of normal appearance (Figure 8A and Table 5). Only two of the 270 embryos of the WT control displayed a similar appearance as 59 abnormal embryos (Table 5). Normal and abnormal embryos of WT/−1C plants segregated in the ratio 3.07:1 (181:59), which fitted the expected 3:1 Mendelian segregation ratio (χ^2^, *p* > 0.88) (Table 5). Scutellum and root part of the embryonic axis were present in the *Hvmpk6* mutant (−1C/−1C) embryos; however, they were typically lacking a shoot (upper) part of the embryonic axis (Figure 8A). Only in some of the −1C/−1C embryos, we observed hints of the reduced/abnormal shoot part of the embryonic axis (Supplementary Figure 9). There were two morphologically different versions of the −1C/−1C embryos. The first one was usually larger, often of a concave disk shape, and hollow at an abaxial side of the scutellum (Figure 8A, −1C/−1C v1). The second one was more of a flat disk shape, usually smaller and of a normal appearance at an abaxial side of the scutellum (Figure 8A, −1C/−1C v2). The first version (v1) was more abundant than the second one (v2). *Hvmpk6* mutant (−1C/−1C) grains containing morphologically abnormal embryos were of similar size and appearance as the WT and WT/−1C grains of the WT/−1C plants (Figure 8B).

**Table 5:** Characterization of the embryos extirpated from the mature grains of heterozygous mutant (WT/−1C) plants.

**Transgenic generation**	**Parental genotype**	**Grains analyzed**	**Normal embryo**	**Abnormal embryo**	**Ratio of normal to abnormal**	**χ^**2**^ value (3:1)**	***p*-value**
T3	A1, A2 (WT/-1C)	240	181	59	3.07:1	0.022	>0.88 n.s.
	WT	270	268	2	n.d.	n.d.	n.d.

**Figure 9:**
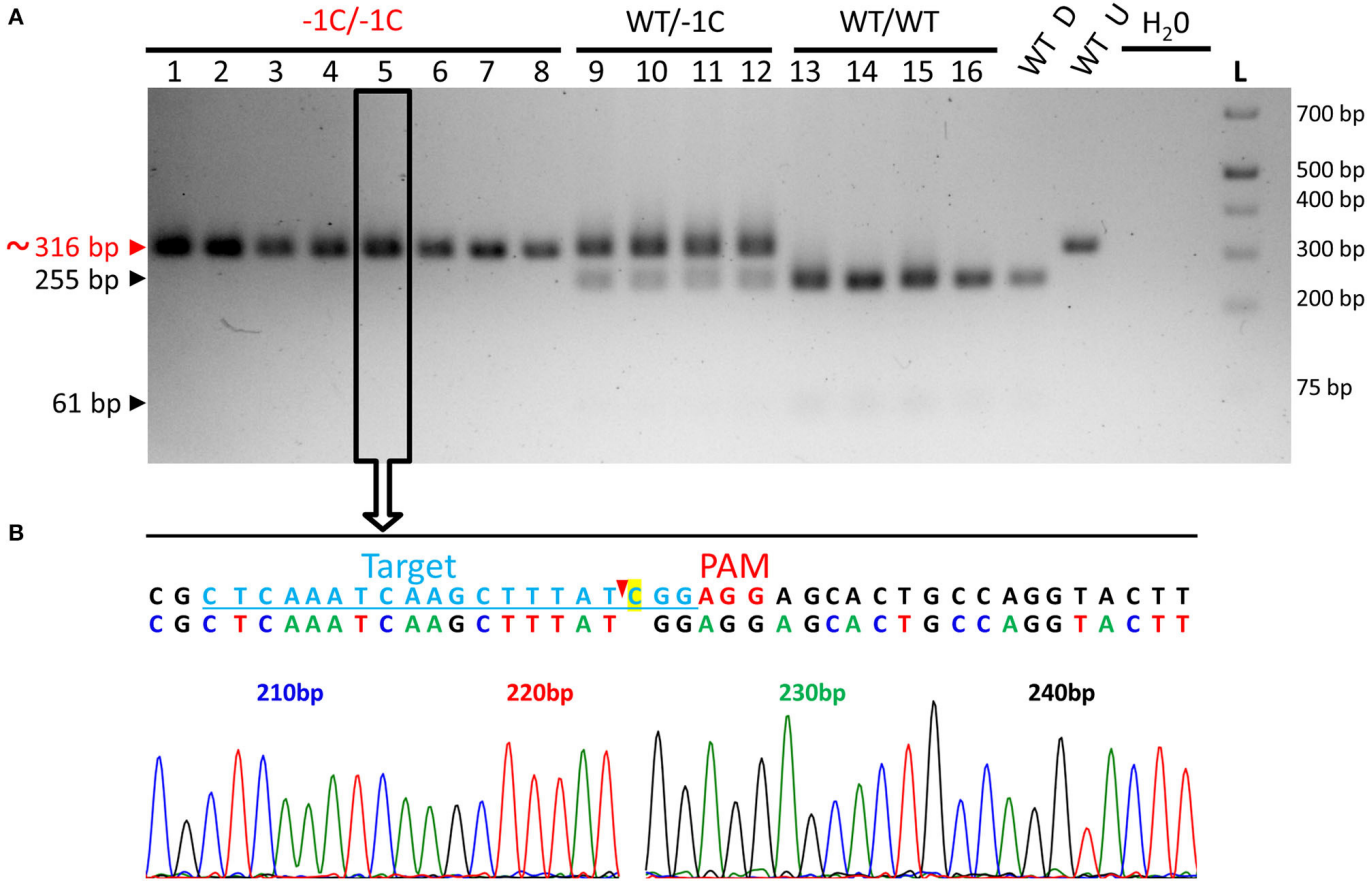
Genotyping of the embryos extirpated from the grains of WT/−1C plants for the on-target mutations in the *HvMPK6* gene - representative results. **(A)** PCR-RE genotyping. Mature T3-generation grains of heterozygous transgenic plants and mature grains of WT non-transgenic control plants were imbibed in Milli-Q water at 21°C in darkness for 18 or 24 h before embryo extirpation and subsequent analysis. Following embryo extirpation and imaging, genomic DNA was extracted from the morphologically abnormal and morphologically normal embryos and/or the respective endosperms with most of the intact bran of transgenic grains and also from the same material of control WT grains. About 316-bp PCR amplicons of the genomic region covering the on-target site, which were associated with eight abnormal and eight normal transgenic embryos were digested with *Hpy*188I. The red arrowhead and red typing of the size of the PCR product indicate the undigested PCR amplicons contaning mutation which disrupts the *Hpy*188I recognition site. The black arrowheads and black typing of the sizes of the PCR products indicate the digested WT PCR amplicons. Wt D—digested and Wt U—undigested reference 316-bp amplicons associated with an endosperm with most of the bran of a WT grain. L-O'GeneRuler 1 kb Plus DNA Ladder (Thermo Fisher Scientific, Waltham, MA, USA). Sizes of the selected ladder fragments are shown on the right. Eight abnormal embryos were homozygous knockout mutants in the *HvMPK6* gene (−1C/−1C) four normal embryos were heterozygotes (WT/−1C), and four normal embryos were WT (WT/WT). Selected PCR amplicons were also sequenced (an example for a −1C/−1C mutant in an open rectangle with an arrow). **(B)** Single sequencing chromatogram associated with the −1C/−1C embryo. Above the chromatogram, deduced color-coded nucleotide sequence and reference WT nucleotide sequence including the position of the on-target site (underlined lightly blue typing) and the PAM site (red typing) are shown. The nucleotide numbering is shown as the distance in bp from the first nucleotide of the K6E3_F1 primer site. Cas9 endonuclease from *S. pyogenes* cleaves target DNA sequences most frequently after the third nucleotide upstream of a PAM site (Jinek et al., 2012; Ran et al., 2015) (red arrowhead). Single base pair CG deletion (“C” highlighted in yellow in a reference sequence) occurs next to the cleavage site in the third nucleotide position upstream of the PAM site. Deduced nucleotide sequence corresponds to the WT nucleotide sequence except for the CG deletion.

Previously, it was reported that PCR-based genotyping of the endosperm with bran and of the embryo from the same caryopsis gives the same results (von Post et al., 2003). Therefore, to verify the genotype of the embryos extirpated from the grains of WT/−1C plants, we genotyped 42 embryos of abnormal appearance and 26 embryos of normal appearance and/or respective endosperms with most of the intact bran using PCR-RE (Figure 9A). The results of this analysis showed that 40 abnormal embryos were biallelic *Hvmpk6* mutants, and only 2 abnormal embryos were heterozygous mutants whereas out of 26 normal embryos, 13 were heterozygous mutants, 13 were WT, and none was a biallelic *Hvmpk6* mutant (Figure 9A). Next, we sequenced about 316-bp PCR amplicons associated with the 15 biallelic mutant embryos and six heterozygous mutant embryos. An analysis of the sequencing data confirmed that all 15 biallelic mutant embryos were homozygotes of the −1C *Hvmpk6* allele (−1C/−1C genotype) (Figure 9B) and that all six heterozygous mutant embryos were heterozygotes of the WT *HvMPK6* allele and −1C *Hvmpk6* allele.

The absence of the shoot part of the embryonic axis or its substantial reduction correlates well with the shootless phenotype of the −1C/−1C seedlings (Figures 6A,D) and indicates that the later-stage defect is determined already during the development of the −1C/−1C embryos. The presence of the root part of the embryonic axis is in line with the presence of the root system in the −1C/−1C seedlings (Figures 6A,B). Also, it is obvious that the majority of the −1C/−1C embryos are arrested in the development (cannot grow or grow insufficiently) because the majority of the −1C/−1C grains do not germinate in the soil (Figure 6E and Table 4). In conclusion, *Hvmpk6* mutant (−1C/−1C) grains contain abnormal embryos, which predetermine the shootless appearance of the *Hvmpk6* mutant (−1C/−1C) seedlings and in most of the cases prevent the grain germination.

**Figure 10:**
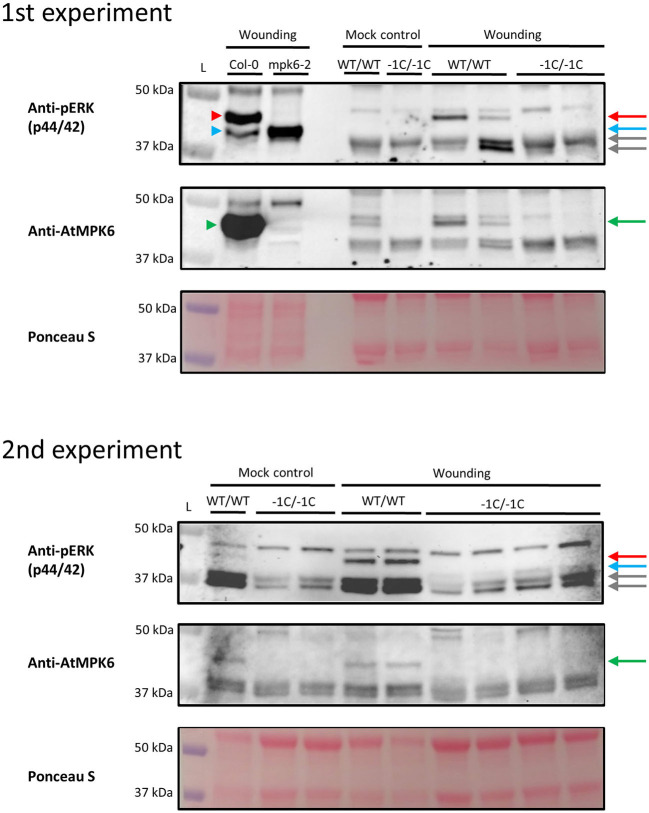
Immunoblot detection of phosphorylated pHvMPK6 and HvMPK6 in the *Hvmpk6* mutant (−1C/−1C) and WT (WT/WT) non-transgenic endosperms with bran and husk. Two representative independent experiments (first experiment and second experiment) are shown. The whole immunoblotted membranes were probed first with an anti-pERK 1/2 antibody and subsequently restriped with an anti-AtMPK6 antibody. For loading control, the whole blotted membranes were stained with Ponceau S. Selected area of the membranes is shown. Mature T3-generation grains of heterozygous mutant (WT/−1C) transgenic plants and mature grains of WT (WT/WT) non-transgenic control plants were imbibed in Milli-Q water at 21°C in darkness for 24 h before embryo extirpation and subsequent analysis. Following embryo extirpation, endosperms with bran and husk were immediately frozen [basal phosphorylation of mitogen-activated protein kinases (MAPKs), mock control] or immediately squeezed and incubated at room temperature for 15 min before freezing (phosphorylated MAPKs, wounding). −1C/−1C endosperms with bran and husk were identified based on the abnormal phenotype of the respective extirpated embryos and subsequent PCR-based genotyping of these embryos. Samples from *in vitro* grown wounded seedlings of *Arabidopsis* Col-0 ecotype (Col-0) and *mpk6-2* mutant (*mpk6-2*) were used as references. The red arrowhead indicates the phosphorylated *Arabidopsis* pMPK6 (anti-pERK 1/2) that is present in Col-0 but missing in *mpk6-2*, and the green arrowhead indicates *Arabidopsis* MPK6 (anti-AtMPK6), which is present in Col-0 but missing in *mpk6-2*. Light blue arrowhead indicates another phosphorylated *Arabidopsis* pMPK (anti-pERK 1/2), most probably pMPK3, as it is more abundant in *mpk6-2* than in Col-0 possibly to partially compensate for the absence of MPK6 (Genenncher et al., 2016). The red arrow indicates phosphorylated barley pHvMPK6 (anti-pERK 1/2), which is present in WT/WT but missing in the −1C/−1C endosperms with bran and husk and the green arrow indicates barley HvMPK6 (anti-AtMPK6), which is present in WT/WT but missing in the −1C/−1C endosperms with bran and husk. The light blue arrow indicates another phosphorylated barley pMPK (anti-pERK 1/2), possibly barley pMPK3 (pHvMPK3), as it is detected only in wound treated −1C/−1C endosperms with bran and husk but missing in the mock-treated mutant −1C/−1C endosperms with bran and husk. Similar to *Arabidopsis* pMPK3, a higher phosphorylation level of pHvMPK3 may partially compensate for the absence of HvMPK6. The gray arrows indicate two unknown proteins (anti-pERK1/2), which are highly phosphorylated and/or abundant in WT/WT endosperms with bran and husk irrespectively of the treatment conditions (mock and wounding) but reduced or missing in −1C/−1C endosperms with bran and husk. L-Precision Plus Protein™ Dual Color Standards (#1610374, BioRad, Hercules, CA, USA). Sizes of the selected ladder fragments are shown on the left of the membranes. Full scans of the whole immunoblotting membranes are shown in Supplementary Figures 11, 12.

To verify the knockout effect of the −1C/−1C mutation in the protein level, we analyzed the −1C/−1C endosperms with bran and husk and the same material of the control WT grains using immunoblot with commercial anti-pERK (p44/42) and anti-AtMPK6 antibodies (Figure 10). Commercial anti-pERK (p44/42) antibodies detect phosphorylated plant pTEpY MAPKs including *Arabidopsis* pMPK6, pMPK4, and pMPK3 (Frei dit Frey et al., 2014; Smékalová et al., 2014; Genenncher et al., 2016; Samakovli et al., 2020). To activate MAPKs, endosperms with bran and husk were squeezed (wounded) and incubated at room temperature for 15 min before analysis. The anti-AtMPK6 antibody was commercially raised against the 12-amino acid long C-terminal epitope (amino acids 384–395) of *Arabidopsis* MPK6 (Supplementary Figure 10). We found that first 11 amino acids of this epitope share 63.6% identity and 90.9% similarity with the corresponding C-terminal stretch of HvMPK6 amino acids (Supplementary Figure 10), suggesting that the anti-AtMPK6 antibody may be also suitable for the specific detection of HvMPK6. In support of this observation, HvMPK6 was first hit (value of *E* was 8.8) in the output of the BLASTP search of the barley IBSC_v2 protein database with the 12-amino acid epitope sequence of AtMPK6 (Supplementary Table 2 and Data Sheet 1). The second hit was HvMPK4 (value of *E* was 17 and 59) and among the rest of the 100 hits in total, no other MAPK was present (Supplementary Table 2 and Data Sheet 1). Immunoblot analysis with anti-pERK antibody showed the specific detection of the phosphorylated barley pHvMPK6 (Figure 10 and Supplementary Figures 11, 12). pHvMPK6 was detected in the protein extracts of the squeezed WT endosperms with bran and husk, sometimes at a low level in the non-squeezed WT endosperms with bran and husk (Figure 10: second experiment), but not in the protein extracts of the squeezed −1C/−1C endosperms with bran and husk. A band corresponding to the pHvMPK6 was approximately at the same position on the membranes as the band corresponding to the phosphorylated *Arabidopsis* pAtMPK6. This observation is in accordance with the similar predicted molecular weight of AtMPK6 and HvMPK6 of about 45.06 and 44.27 kDa, respectively (Supplementary Figure 10). We also probably detected pHvMPK3 protein (the predicted molecular weight is 42.82 kDa) exclusively in the extracts of the squeezed (wounded) −1C/−1C endosperms with bran and husk (Figure 10). The weak band corresponding to this protein was present on the membranes approximately at the same position as the reference pAtMPK3 (42.72 kDa). In three of the experimental replicates, we could see this band clearly (Figure 10: second experiment), and in the remaining three replicates it was less obvious (Figure 10: first experiment). Furthermore, in four out of six experiments, we detected two unknown highly phosphorylated and/or abundant proteins of similar molecular weight of about 37 kDa in the protein extracts of WT endosperms with bran and husk irrespective of the treatment conditions (Figure 10: second experiment). In the two remaining experiments, the larger of these two proteins was less obviously detected in WT endosperms with bran and husk (Figure 10: first experiment). The two 37 kDa proteins were less phosphorylated and/or abundant in the protein extracts of the −1C/−1C endosperms with bran and husk (Figure 10). The reduction of the 37 kDa proteins was not due to the off-target activity of the gRNA/Cas9 complex in the *MAPK* genes (see Section Off-Target Mutation Analysis) and most probably it was a secondary effect of the *HvMPK6* gene knockout. Immunoblot analysis with an anti-AtMPK6 antibody showed the specific detection of HvMPK6 in the protein extracts of WT endosperms with bran and husk, but not in the protein extracts of −1C/−1C endosperms with bran and husk (Figure 10). The band corresponding to HvMPK6 was approximately at the same position on the membranes as the band corresponding to the *Arabidopsis* AtMPK6, likewise, it was at approximately the same position as the band corresponding to the phosphorylated pHvMPK6. One of the HvMPK4 isoforms has a predicted molecular weight of 44.03 kDa, and concerning the possible cross-reactivity with anti-AtMPK6 antibody (Supplementary Table 2 and Data Sheet 1), its presence may interfere with the detection of HvMPK6 (45.06 kDa). However, HvMPK4 cannot be absent in the −1C/−1C endosperms with bran and husk because of the unintended off-target activity (see Section Off-Target Mutation Analysis). Accordingly, it should have been detected not only in WT, but also in the −1C/−1C endosperms with bran and husk. Since we did not detect any about 44 kDa protein in the −1C/−1C endosperms with bran and husk (Figure 10), we assume that 44.03 kDa HvMPK4 was absent or present in undetectable amounts in the protein extracts of the endosperms with bran and husk. Alternatively, anti-AtMPK6 antibody is not immunoreactive toward HvMPK4. In conclusion, we specifically detected pHvMPK6 and HvMPK6 in WT protein extracts but not in the *Hvmpk6* mutant (−1C/−1C) protein extracts, hereby confirming the loss-of-function effect of the −1C/−1C genotype in the pHvMPK6 and HvMPK6 protein level.

### Off-Target Mutation Analysis

Based on the CRISPOR *in silico* analysis, the selected guide CTCAAATCAAGCTTTATCGG has high cleavage specificity in the GP v1 genome assembly with Massachusetts Institute of Technology (MIT) (Hsu et al., 2013) and cutting frequency determination (CFD) (Doench et al., 2016) specificity score of 70 and 62, respectively. All the respective off-targets predicted in the GP genome had at least three mismatches and the highest CFD off-target score was 0.42714497 out of one possible (Supplementary Table 3). The CFD score was shown to be the most accurate among the four different off-target scores (Concordet and Haeussler, 2018). To evaluate the off-target cleavage efficiency, we analyzed the two most probable off-target sites CABVVH010000001.1 215.57 Mbp (CFD = 0.42714497) and CABVVH010000002.1 489.51 Mbp (CFD = 0.415384615) in the genomes of emerged T3-generation A1 plants by PCR-Seq (Supplementary Tables 1, 3 and Supplementary Figure 13). None of these sites was edited in the genomes of all 12 analyzed plants (Data Sheets 2, 3). Off-targets in exons are more likely to have a functional impact. The predicted GP off-targets were present within exons of six genes (Supplementary Table 3). Nevertheless, the highest CFD score of exonic off-target was 0.078755469, more than five times lower than the CFD scores of the off-targets assayed by PCR-Seq (Supplementary Table 3). Moreover, except for one off-target sequence, all the exonic off-targets had four mismatches with at least one mismatch in the 8-bp seed region proximal to the PAM. Such off-targets are virtually non-editable in plants (Modrzejewski et al., 2020). One exonic off-target had three mismatches; however, it also had non-canonical PAM and was present in the two Morex but not GP genes. Intronic GP off-targets were present within 10 genes (Supplementary Table 3). Based on the number and position of the mismatches and/or CFD score, all the intronic off-targets were highly unlikely to be edited. None of the predicted exonic and intronic off-targets was present within a *MAPK* gene (Supplementary Table 3). Moreover, our analysis of GP intergenic off-targets (see Section Off-Target Mutation Analysis) showed that none of them was present in the genomic regions closely surrounding the *MAPK* genes. The shortest distance between the off-target site and the *MAPK* gene was more than 151,000 bp.

## Discussion

Mitogen-activated protein kinases, the last tier of MAPK modules, are the key regulators of plant development (Smékalová et al., 2014; Bigeard and Hirt, 2018; Komis et al., 2018). The role of particular *MAPK* genes in plant development could be efficiently revealed by reverse genetics studies, including T-DNA insertional mutagenesis and genome editing by CRISPR/Cas9 technique (Bush and Krysan, 2007; Müller et al., 2010; Minkenberg et al., 2017; Wang et al., 2017; Chen et al., 2018; Samakovli et al., 2020). Here, we aimed at the preparation of the transgenic barley plants with the *HvMPK6* gene knocked-out by CRISPR/Cas9 and the evaluation of their early developmental phenotypes.

To develop an *Hvmpk6* loss-of-function mutant, we designed and prepared a gRNA/Cas9 construct CC9-K6E3, which is based on p6i-d35S-TE9 + pSH91 plasmids and targets the third exon of the *HvMPK6* gene. The on-target site of the designed gRNA is present in the coding region of all known transcription variants associated with the *HvMPK6* gene, except for one, which, however, encodes only a short peptide (33 aa). Using *Agrobacterium*-mediated transformation of the barley variety GP with the CC9-K6E3 construct, we obtained 13 independent transgenic barley lines in T0 generation. However, only one T0 line, designated as line A, was identified to carry the mutation in the on-target region of the *HvMPK6* gene. This line was a chimera composed more of WT cells than of mutated cells and among its 37 analyzed T1 progeny, only six plants were mutated in the on-target region. Three of the six mutated plants were chimeras and the three remaining plants, designated as A1, A2, and A3, were viable heterozygous mutants, which carried heritable single base pair CG deletion in the third nucleotide position upstream of the PAM in the mutated *Hvmpk6* allele (−1C allele). In this study, we have shown that −1C/−1C homozygous knockout mutation in the *HvMPK6* gene is lethal causing embryo arrest or shootless phenotype of the abnormal seedlings. The lethality of the *HvMPK6* knockout may be reasoning the low number of the obtained mutated lines in T0 generation. Loss-of-function mutations in the *OsMPK1* gene, a rice ortholog of the *HvMPK6* gene, were shown to be embryo lethal (Yi et al., 2016; Minkenberg et al., 2017) and similar difficulties in obtaining mutated lines were reported for the targeting of *OsMPK1* with a PTGb3 CRISPR/Cas9 double-target gRNA construct (Minkenberg et al., 2017). From the three independent transformations of 600 calli of cultivar Kitaake (*Oryza sativa* ssp. *japonica*) with PTGb3, only four hygromycin-resistant calli were recovered and only two independent viable mutated lines were obtained in T0 generation. These lines, designated as PTGb3-1 and PTGb3-2, were biallelic mutants in a single-on-target region in the first exon of the *OsMPK1* gene. Concerning genotype constitution, PTGb3-1 and PTGb3-2 plants were similar to the heterozygous mutant A1, A2, and A3 plants (all WT/−1C), which we obtained in T1 generation, as each carried an open reading frame preserving −6 bp deletion in one allele (equivalent to the WT allele) and either −5 bp knockout deletion or +1 bp knockout insertion in the other allele (Minkenberg et al., 2017).

When editing a genome using the T-DNA-based approach, it is desirable to segregate away T-DNA insertion/insertions in the desired mutant lines. However, we could not select T-DNA-free mutant plants in the progeny of line A. In T1 generation, all three heterozygous mutant plants A1, A2, and A3 as well as further analyzed chimeric plant A4 contained the CC9-K6E3 construct. The construct was not segregating in the T2 progeny of these plants. We genotyped 188 T2 progeny plants of A1, A2, A3, and A4 in total and found that all contained T-DNA in their genomes. Also, in T3 generation, all 60 analyzed progeny plants of A1 and A2 heterozygous mutants harbored T-DNA in their genome. Most probably, two or more CC9-K6E3 T-DNA insertions were present in the genome of parental plant of A line in T0 generation, and these insertions were segregating in its progeny.

Because the selection of the T-DNA-free mutant line was not successful in this work, we followed the inheritance of the monoallelic mutation in the *HvMPK6* gene (WT/−1C) together with the CRIPSR/Cas9 cassette from T1 generation to T3 generation. In rice, the co-inheritance of monoallelic mutations in the on-target site of the *OsPDS* gene with the respective CRISPR/Cas9 cassette resulted in the appearance of chimeras, unpredicted segregation ratios with more on-target mutants than the theoretically expected and identification of new on-target mutations, different from the original parental mutations (Ishizaki, 2016). Also, in hexaploid wheat, the trans-generation activity of the gRNA/Cas9 complex resulted in the occurrence of new mutations at the on-target sites of the *TaGW2* and *TaLpx-1* genes of the T1- and T2-generation plants (Wang et al., 2018). Likewise, in barley, the gRNA/Cas9 complex targeting the *HvPM19-1* gene was probably active in both T0 and T1 generations, as all T1-generation on-target-edited plants retained their corresponding T-DNA construct, while it was segregating in the non-mutated T1 plants (Lawrenson et al., 2015). Targeting of the *HvPDS* gene with CRISPR/Cas9 led to a high level of the on-target somatic editing in the T0 generation barley lines (Howells et al., 2018). Multiple genotypes were obtained within a single leaf sample within a single plant in five of the six generated lines. Moreover, different series of edits were identified when plants were resampled later.

In this work, T0-generation line A was a chimera and three of its six mutated T1 progeny plants were also chimeras. However, the three remaining plants were all heterozygous mutants (WT/−1C) and in the progeny of the two of these plants, designated A1 and A2, we did not observe substantial effects of the co-inheritance of the CC9-K6E3 cassette with the monoallelic −1C mutation. In the T2 generation, using PCR-Seq genotyping, we identified 85 Cas9-edited plants in the emerged progeny of A1 and A2, out of which only four plants were chimeras. The remaining 81 plants were monoallelic mutants and nearly all of them (75) were heterozygotes of the WT and −1C allele as their respective parents suggesting transmission of the −1C allele from the parental T1 generation. Only six monoallelic mutants were heterozygotes of the WT and mutated *Hvmpk6* allele different from the −1C allele. Also, PCR-Seq analysis of the T3-generation biallelic *Hvmpk6* mutant seedlings and grains showed that all 10 analyzed seedlings and 19 grains (four non-germinated grains from the soil and 15 grains directly analyzed) were homozygotes of the −1C allele (−1C/−1C), suggesting that mutant *Hvmpk6* alleles different from the parental −1C allele were not present or rare in the T3-generation progeny of the WT/−1C parents. In the T3 progeny of A1 and A2 (both WT/−1C), SREN was 3.75:1 (150 emerged plants/40 non-emerged) and fitted the expected Mendelian 3:1 ratio of heterozygous and WT to mutant plants (χ^2^, *p* > 0.20 n.s.). Moreover, 240 T3-generation grains collected from A1 and A2 heterozygotes (both WT/−1C) contained 181 normal embryos and 59 abnormal embryos, providing the 3.07:1 ratio, which fitted the expected 3:1 ratio (χ^2^, *p* > 0.88 n.s). Only in T2 progeny of WT/−1C plants, SREN was 1.94:1 (165/85) and it did not fit the 3:1 ratio (χ^2^, *p* < 0.01 n.s.). Contrary to the abovementioned reports, we have targeted the gene, a knockout of which is lethal and this may partially explain the absence of the substantial effects of the co-inheritance of the CC9-K6E3 cassette with the monoallelic −1C mutation. Also, Ishizaki (2016) reported that four out of 11 independent rice lines edited in the *OsPDS* gene segregated in a Mendelian fashion in on-target induced mutations in the T1 generation despite the presence of the inherited CRISPR/Cas9 T-DNA. Similarly, the ongoing activity of the gRNA/Cas9 complex designed for the targeting of the *TaPDS* gene did not result in any additional on-target edits in five edited and seven non-edited wheat transgenic lines in the T1 generation (Howells et al., 2018). Taken together, these observations and our results suggest that in plants, on-target mutations induced by an inherited gRNA:Cas9 complex may occur at non-significant levels or may not occur at all.

CRISPR/Cas9 may also edit off-target sites. Moreover, if the respective T-DNA cassette is not segregated away in mutant lines, the activity of the inherited gRNA/Cas9 complex may eventually lead to the accumulation of the off-target mutations in the subsequent generations. In this work, we performed experiments with T2- and T3-generation plants and grains. To check if the activity and inheritance of the CC9-K6E3 T-DNA resulted in unintended genome edits, we analyzed the two most probable off-target sites in the genomes of 12 plants from T3 generation using PCR-Seq. None of these sites was edited in the genomes of all 12 analyzed plants, suggesting that in addition to the absence of the substantial extra on-target activities, the inherited gRNA/Cas9 complex did not also edit off-target sites. Furthermore, based on the number and position of the mismatches and/or probability score, exonic and intronic off-target sites were highly unlikely to be edited. Collectively, these data strongly suggested that the off-target DNA cleavage did not interfere with the conclusions of this work.

In rice, homozygous knockout mutations in the *OsMPK6*, an ortholog of *HvMPK6*, were reported to be embryo lethal (Yi et al., 2016; Minkenberg et al., 2017). Approximately one-quarter of the mature grains of PTGb3-1 and PTGb3-2 rice lines, CRISPR/Cas9-induced biallelic heterozygous mutants in the first exon of the *OsMPK1* gene (in this work *OsMPK6* is designated as *OsMPK1*), did not germinate as well as approximately one-quarter of these grains contained an abnormally small embryo (Minkenberg et al., 2017). Similarly, approximately one-quarter of the grains of heterozygous 3A-60391 (*OsMPK6/Osmpk6-1*) and 2A-10337 (*OsMPK6/Osmpk6-2*) rice lines, which harbor T-DNA insertion in the fifth intron and sixth exon of *OsMPK6*, respectively, did not germinate (Yi et al., 2016). The authors showed that both *Osmpk6-1* and *Osmpk6-2* mutant mature grains contained abnormally small and flat embryos, which were arrested early in the development, in the globular stage, missing embryonic organs. Also, in our study, approximately one-quarter of the grains collected from the heterozygous mutant (WT/−1C) plants contained abnormal embryos, and we showed that nearly all of these were homozygous knockout mutants (−1C/−1C). However, −1C/−1C embryos were of similar size to the embryos of WT/−1C and WT mature grains. Moreover, they were late-stage developed embryos containing scutellum and the root part of the embryonic axis. The morphologically visible defects were manifested by the absence of the shoot part of the embryonic axis or its substantial reduction and in the majority of embryos also by the hollow shape of the abaxial side of the scutellum. The majority of the *Hvmpk6* mutant (−1C/−1C) grains did not germinate in the soil. Nevertheless, a minority of them developed into abnormal seedlings with a shootless phenotype, suggesting that some of the abnormal embryos were capable of limited postembryonic development. In conclusion, *HvMPK6* appears to have a less critical and also, to a certain extent, different role in the barley embryo development than *OsMPK6* in the rice embryo development.

HvMPK6 seems to be essential for the shoot formation as *Hvmpk6* mutant (−1C/−1C) is shootless. In addition to the shootless phenotype, −1C/−1C seedlings also have a short root system. However, since the root system of the −1C/−1C mutants is relatively often otherwise well-developed, it is possible that the disruption of the *HvMPK6* gene itself is not limiting the growth of the roots, but rather it is restricted by the absence of the assimilate transport from the non-existing shoot. There is a report suggesting that MPK6 is less critical for the shoot development in rice (Minkenberg et al., 2017). As a rare event, Minkenberg et al. (2017) recovered from a single hygromycin-resistant callus of the line PTGb3-4 two plantlets. The PTGb3-4 line carried CRISPR/Cas9 double-target gRNA-induced chromosomal fragment deletion in the first exon of *OsMPK1* (in this work *OsMPK6* is designated as *OsMPK1*). The plantlets were severely dwarfed and produced only one sterile panicle (Minkenberg et al., 2017). Obviously, *Osmpk6* knockout mutant described by Minkenberg et al. (2017) developed shoots.

In *Arabidopsis*, only the *mpk3*/*mpk6* double mutant is embryo lethal (Wang et al., 2007). Initially, no obvious developmental phenotypes were observed in single loss-of-function *Arabidopsis mpk6* mutants (Wang et al., 2007). Later, it was shown that loss-off *MPK6* function has pleiotropic effect with a limited penetration on *Arabidopsis* development and that most of the defects are determined already during the embryo development (Bush and Krysan, 2007; Müller et al., 2010; López-Bucio et al., 2014; Zhang et al., 2017). Originally, Bush and Krysan (2007) reported that the minority of the *mpk6* mutant seeds were defective with abnormal embryos that had burst out of their seed coats. Furthermore, López-Bucio et al. (2014) described three phenotype categories of the mature seeds of *mpk6* mutant: minor categories of the burst-out embryo and raisin-like seeds and the major category of bigger seeds. They found that these categories were predetermined already during the embryo development with burst-out, raisin-like, and bigger seeds correlating with the embryos arrested at the heart stage, embryos arrested in the development but developing hypocotyl and root, and embryos completing embryonic organogenesis, respectively. Moreover, the burst-out seeds developed only adventitious roots while raisin-like seeds, and bigger seeds developed shorter and longer roots, respectively, than the seeds of WT at six days after germination. Taken together these observations suggested that the embryonic defects predetermined not only seed morphology but also subsequent seedling development. Zhang et al. (2017) also tracked back raisin-like and burst-out seed morphology to the abnormalities in the embryonic development and showed that defective embryogenesis of the *mpk6* mutant is maternally determined. In addition, the minority of the *Arabidopsis mpk6* mutant seedlings (16–44%) displayed an early postembryonic defect in the primary root formation and growth including temporarily “no root” phenotype (Müller et al., 2010). In this work, we also observed that embryonic defect predetermined the abnormal seedling development of the barley *mpk6* mutant. However, in *Arabidopsis*, embryonic defects of *mpk6* mutants affected root but not shoot phenotypes of seedlings, whereas, in barley, the embryonic defect of *mpk6* mutant resulted in seedling shootless phenotype. The shootless phenotype was described for *Arabidopsis*, rice, and maize mutants defective in genes regulating the shoot apical meristem formation (Barton and Poethig, 1993; Aida et al., 1999; Satoh et al., 1999; Pilu et al., 2002; Scofield et al., 2014). Nevertheless, none of these genes was reported to encode MAPK. To our knowledge, we provide the first report linking plant *mpk* mutant with a shootless phenotype.

## Data Availability Statement

The raw data supporting the conclusions of this article will be made available by the authors, without undue reservation.

## Author Contributions

PK and PV wrote the manuscript with input from all the co-authors. PK designed the experiments, analyzed, evaluated, processed data, and performed or participated in all the experiments except for the Western blot analysis and barley transformation. EC performed a larger portion of the T-DNA and mutation genotyping, participated in the extirpation of embryos, and performed grain sowing. PV performed the Western blot analysis. LO and TV performed a stable barley transformation using the method of Harwood (2014) and contributed to the embryo extirpation experiments. VB coordinated a stable barley transformation using the method of Marthe et al. (2015) and provided an infrastructure for it. PC performed ploidy measurement. MO contributed to a microscopic analysis of extirpated embryos using the Zoom microscope. JŠ provided funding and infrastructure for this research, supervised the project, and edited the manuscript. All authors contributed to the article and approved the submitted version.

## Conflict of Interest

The authors declare that the research was conducted in the absence of any commercial or financial relationships that could be construed as a potential conflict of interest.

## Publisher's Note

All claims expressed in this article are solely those of the authors and do not necessarily represent those of their affiliated organizations, or those of the publisher, the editors and the reviewers. Any product that may be evaluated in this article, or claim that may be made by its manufacturer, is not guaranteed or endorsed by the publisher.

## Supplementary Material

Supplementary Table 1 Overview of oligonucleotides used in this study.

Supplementary Table 2 BLASTP: Specificity of the “REALAFNPEYQQ” epitope toward HvMPK6.

Supplementary Table 3 Overview of the off-targets predicted by CRISPOR.

Supplementary Figure 1 The transcribed portion of the genomic sequence of the *HvMPK6* gene.

Supplementary Figure 2 Comparison of the transcripts associated with the *HvMPK6* gene.

Supplementary Figure 3 Superimposed sequencing chromatograms associated with plants chimeric in the induced mutations.

Supplementary Figure 4 Ploidy estimation of the transgenic line A.

Supplementary Figure 5 Alignment of the HvMPK6 amino acid sequence with the amino acid sequences of putative proteins resulting from CRISPR/Cas9-edited *HvMPK6*.

Supplementary Figure 6 *HvMPK6* alleles of the T2 progeny plants of a chimeric A4 plant.

Supplementary Figure 7 Phenotypic comparison of the abnormal transgenic WT and *Hvmpk6* mutant (−1C/−1C) seedlings.

Supplementary Figure 8 A rare example of the abnormal seedling heterozygous in the *HvMPK6* gene (WT/−1C), which, early after sowing, showed some signs of shoot emergence.

Supplementary Figure 9 Examples of the *Hvmpk6* mutant (−1C/−1C) embryos with hints of the shoot part of the embryonic axis.

Supplementary Figure 10 Alignment of the barley HvMPK6 and *Arabidopsis* AtMPK6 amino acid sequences.

Supple mentary Figure 11 Western blot analysis of the *Hvmpk6* mutant (−1C/−1C) and WT (WT/WT) endosperms with bran and husk – 1st experiment.

Supplementary Figure 12 Western blot analysis of the *Hvmpk6* mutant (−1C/−1C) and WT (WT/WT) endosperms with bran and husk – 2nd experiment.

Supplementary Figure 13 PCR sequencing (PCR-Seq) genotyping of the T3-generation plants for the presence of the mutations in the predicted off-target sites.

Data Sheet 1 BLASTP: specificity of the “REALAFNPEYQQ” epitope toward HvMPK6.

Data Sheet 2 Alignment of the 344-bp nucleotide sequence covering the CABVVH010000001.1 215.57 Mbp off-target site with the respective.ab1 files of 12 analyzed T3-generation plants.

Data Sheet 3 Alignment of the 460-bp nucleotide sequence covering the CABVVH010000002.1 489.51 Mbp off-target site with the respective.ab1 files of 12 analyzed T3-generation plants.
