# Supplementary material for: CRISPR/Cas9-Induced Loss-of-Function Mutation in the Barley Mitogen-Activated Protein Kinase 6 Gene Causes Abnormal Embryo Development Leading to Severely Reduced Grain Germination and Seedling Shootless Phenotype
Source: Front Plant Sci. 2021 Jul 30;12:670302. doi: 10.3389/fpls.2021.670302 (PMC8361755; doi:10.3389/fpls.2021.670302)
Supplement: Supplementary Table 1 — Overview of oligonucleotides used in this study. [file Table_1.DOCX]

| **Method** | **Gene** | **Primer/Probe** | **5'-3' sequence** | **Amplicon size** |
| --- | --- | --- | --- | --- |
| Spacer cloning | *HvMPK6* | sK6E3_F  sK6E3_R | GGCGCTCAAATCAAGCTTTATCGG  AAACCCGATAAAGCTTGATTTGAG | NA-Oligo annealing |
|  |  |  |  |  |
| TaqMan Multiplex qPCR | *Hpt*^a^ | PHptF1  PHptR1  HptP | GCGGATTTCGGCTCCAA  CTCCAGTCAATGACCGCTGTT  FAM-tcctgacggacaatggccgca- TAMRA | 65 bp |
|  |  |  |  |  |
|  | *CO2*^b^ | HvCon2F1  HvCon2R1 | TGCTAACCGTGTGGCATCAC  GGTACATAGTGCTGCTGCATCTG | 106 bp |
|  |  | HvCon2P | VIC-CATGAGCGTGTGCGTGTCTGCG-TAMRA |  |
|  |  |  |  |  |
| PCRmutation screening  PCR  off-target screening | *HvMPK6*  NA  NA  NA  NA | K6E3_F1  K6E3_R1  Off215_F2  Off215_R2  Off489_F2  Off489_R2  Off215_F1  Off489_R1 | aagctagacattatctaaggcatgt  GGGTATGAGAAGGGTTTA  ATAAATGGATTGCGGGAGCTTCA  GTACTACCTGAAGGGCGAAAGGAC  CACGGGTCACCTTCCAGTTAG  TCACCTTCTACCGCTCTACCA  AAATGGATTGCGGGAGCTTCAA  TACCGCTCTACCAACGAGGA | 316 bp  344 bp  460 bp  NA-seq. primers |

**Supplementary Table S1.** Overview of oligonucleotides used in this study. NA – not applicable
