# Supplementary material for: CRISPR/Cas9-Induced Loss-of-Function Mutation in the Barley Mitogen-Activated Protein Kinase 6 Gene Causes Abnormal Embryo Development Leading to Severely Reduced Grain Germination and Seedling Shootless Phenotype
Source: Front Plant Sci. 2021 Jul 30;12:670302. doi: 10.3389/fpls.2021.670302 (PMC8361755; doi:10.3389/fpls.2021.670302)
Supplement: Supplementary Figure 1 — The transcribed portion of the genomic sequence of the HvMPK6 gene. [file Data_Sheet_1.pdf]

Supplementary Material

CACCACACCGCACCGCACCGCACCGCACCACCTAAACAAAACAGGAGGCGGAAAGCGACCAA
ATCTCGCGAGCGAATTCCCCTCCCCTCCCTCCCCACGCGCGATCCGGGCGGAG**ATG**GACGC
CGGCGGGGCGCAGCCGCCGGACGCGGAGATGGCGGAGGCCGGGGGGGCCGCCGCGGCGGCCG
CGGCCGCGGGGGCGCCGGGCGGCGCCATGGAGAACATCCAGGCCACGCTCAGCCACGGCGGG
AGGTTCATCCAGTACAACATCTTTGGCAACGTCTTCGAGGTCACCGCCAAGTACAAGCCCCCC
ATCCTCCCCATCGGCAAGGGCGCCTACGGCATCGTCTGgtacgccccatccccatccccatccacatccccttctttccc
ctccgtctccgtctccggctgactcaccgaccgactgacagCTCCGCGCTCAACTCCGAGACGGGGGAGCAGGTGGCCA
TCAAGAAGATCGCCAACGCCTTCGACAACAAGATCGACGCCAAGCGCACGCTGCGGGAGATC
AAGCTGCTCCGCCACATGGACCACGAGAATgtcagtcccccgcccccgatccgccccccctccctccatttcccccccccccc
cccccccccccccccccccccccccccccccccccccccccccccccccccccccccccccccccccccccccnnnnnnnnnnnnnnnnnnn
nnnnnnnnnnnnnnnnnnnnnnnnnnnnnnnnnnnnnnnnnnnnnnnnnnnnnnnnnnnnnnnnnnnnnnnnnnnnnnaagagccc
accaacctcttgggatgcgattcattgtgtgttcttcctcggcgcacctggagcagcaccacctgctcctaccgctagatcccgctgatgtcaaaagctgcctca
ggaccaatctatccgcctgtgtccctttttgctgcctagctcctccgaatccagtggaagtgaacgcacagttcctaggctgggggttactactgtttgaaagttctt
attaggatgttggacttggatctttccattccagtgtcctgtgataactaaactatgtaagagaaggaacctacatctgagttaggcatcttgggtttcttcatgtaaa
aaagctttggcattcttcctgtaatatgagatgaatcatgttttggcgtgtatttgagaggggaaaaatgcattttggaaagacagctgtgctaggagtagactaga
gatgcagagtatcttgacggcctgccttggcaccatagcgtatgctctgagatgtgctgagttgaagtttggatgatgctaagtaatctgctagcaataagagcat
gaagagaataggatccctttggctttctattctttgtttcatcaatcattgtcgtttgcagattgcttattctcgttgaagattttcagagtgaaactggtttttggctaatat
cctgcgtaagctagggtttcagattttaatgctccactggaatagacaaaacttctttgagagtttgaccgtcggaataattaagtctgttagtagtgaaatgttggat
taggcaaggtctgattaataacagaaatgcttgtcgacacgtgctttatacgctgtcagctgtaagtgtatcacctgctgttgtaagtgtatcacctgctgtagcaac
tacaaatgcagaagttttccgttacgagatcctgtactttgttttttttatgctgcgaacgaggatttctcacaagaagtgtcggagacggttgttggtggctggcac
aaaagctgaaatgggcagcgtggcggcaccagtgggtggctcaatagttggctcttatgaacggacagagcaacggccacattgcctaatcatttgttccctg
gaagcaactagacttttcagtgggcttaaggcctggtaacagaaccatagcacaggagagaacatcctgaccataaagaaaaacaacacacaggaccacta
acggcacacatttgcctttatagtagctatggcatgtgatacgcttccctgtcagttatttatgtacaaaacttccataactgtatgatgctgtcttattcattcttattctc
cacgtcaaccccccaaaaggctgagctacataataaagccatagaggtttggagatcctgtattcctgtagctatattagaagctttactttatcattatgtaaggaga
agtgctccctcttggtcccaaaggcacagaaaattacatttccaacttttggaaaaaaaggaacgattttgttaatgtacctgtgcaatatgcgtacaaaaatgcat
gccttgcacaagtttttggtagtacacatattgttttacaatgtacatcaaaatgtcccctctcttttggaaaattgcaaatgcattttcttcaccagggaacatgtgtag
ctaggaattgcctattgttcacctcctaaaccaagtaatattcggcttggcacaactacttggttgatgcacttgtttcatctatgctacctacctacttgtatcttgtttgt
tgtagtgtacacatacataccttaactcttagctgtactcggtatatccatgctctaagcagcaatgactggtcatatccatgctctaagcagcaagatccaagagc
tgttcagaacttcctatactgctcatgtgctatgtggggacatatgcagttggtatagtgctttcttgtgttcaagttagttacacgggttttgaagagggttcacctaa
agacagaggctaccaactaataaaaaacagaaagctccagaactgttttattctgactcccgtatcaactatttatgcattctgctgtttgaaattctgaacgcataa
atttgtggtatctgcactgctaagaaaggcagtcggaaatgtacaaggttacatcctaaaggttacacccttcactcttgaaacatgcaaaataaataaataaatta
ggttgtcatcactagcacctgcatctttgaatcatatttcatatggtgctgtcagaagtactactagctccaggttcttaataatgattattcatttacctcattttctggac
atcataattctgttatgtttttggcgaaaaataataaaatattgctatgattgtgttaataatatgatgccaatttacttctgtcgtgctgtttttgtactccctccgtcccaa
cgtaagtgttgtactaaatccgcgacacttattttgggatggagggagtagaatactatatattcagaaacacgtttaacctaaaaaagaatccaacagttcatcaa
aatgccactgggtgcatatcagctctaacaaatgtagggtttgtagtatccaaaaacaagtttcaaaaggaaatgccaacttgatagaattttagaagtttccatgc
aacttagttaagctgtaaccgtagccaaaacagttaagtgaagtatatgtgttcatcagctttccacgatttgttgattatttcaatgcattcaagatgaaatataaagt
tgtctcaaggcatcgaaagaacgtacagattgtcagctgtaacaatagcagacataagttatatgaactgtatgtgttcttcagctttccatcacctgttgactatttc
aattcattcaagatgaaagataaagctgactcaagccaccgaaagaaagtacagatatgtcaaattgtattgtcttggtgtatgagttctttgtgcaaggtgtaaaa
gctagacattatctaaggcatgtggtcctgttgctgctaaccatagttattgcctttttggaaggaaaaaaaatcaagtatgaattaatgctacaaaatatgtttcttac
aagagttcacttttatgcagATTGTTGCAATAAGGGATATTATACCTCCTGCACAAAGGACTGCATTCAATG
ATGTCTATATTGCATATGAATTGATGGACACCGATCTGCATCAAATTATTCG**CTCAAATCAAG
CTTTATCGG**AGGAGCACTGCCAGgtacttattagttacttcgatatagaagtaaaaccttctcataccccctttgtaggataagtacgcttat
ctaattatattattgtttggctatagaaattcttgtctctagtatctcatttcatctgattccccgagtacattataccgtgttcgtcagttatctgtttcattattatttctgcct
cagctcatacatacttctgtcagatattagtatcctgggagcagggagctaatgagtaataactgaagcctctcgatctttcttctttttggtcggcctgaatcatctt
gctactttgctagtctaggactacgtgctctaaataaatttgcgtggtgtatagtataaacattgaacttagtagtggaattatgggggtatatttgatgactaccggtt
gatattttgcttcactgtaatacaatggaaattggattagtactccgacataggtgaactgtaatatactaacattgaataagtaccttactgtattacacctcttagag
aggatacaacatattaggaccttgcaatgcacccctctgaacattctgtttaggaaagggagcagttgaacaagttcatcacatatagcacatgcctgaacttaat
gctagcaaatccttcttctattctttgtcaaacacacccatgcatcatcattgctaaggctgcccgtaatgggagtatcataggtagtatcatgcgtgtcaactaagc
aatttcgatgaggtggcatagaattaaatgaagaaaaagagagttgagtatcatattaaatgatgtgctactatgtgtcttgcatgtcaataaatgaactaccatatg
atactatgcattacggatgtggtatcatacactagtatcatatgcatgatactagtatatgtcaata

aatgaactaccatatgatactaacatatgatactatgcattacggatgtggtatcatacactagtatcatatgcatgatttttttgttggatttcactccacccgtggttg
ggaactagtaattcgttgggtctataatgattttggatgggctcctagtgagcttattttcactatttttgtttgtagttttccagtaattctagatacatgtttgaaattttgg
gtctttttttgtttaaaccgtctatctccttcgcttgtagtcatttatcattcaattagtgaagtataaattcattcgatctcccgatattaattattaatattaatcaaattagc
atctttcacagaccaacactagtctttcctgtgccctttgtcctcactcatgtgcacccgtggtcaacttcctggtcggtcacccatcctagaattactccaagccaa
gcacacttaactttggagttcttttcgaatgggctcctggaaaagaaggaattccttgttgatatgagtagtcttatcattcatattaagtcaggctctcacaatgattc
cctaaatagaagtactttcttgatatcagagtggtctcttttgttactagctaatattaaatcatgttttactttcccaaataccaccgtataatccttcgtggaaaagttcc
atggaaaaactgtttaccataatatgcctgtattgtcagggcctactcgtgtttctagcataaaaaagttgatgctagatcacacatgagaaattagtggatttctgg
aatgttctcacctatttctcttatgtcgatcttgttctttctagtttcttttcaatctgcaaacttctgtcttgttctgagaaattgtccttcatctgatatggatgttttgtgtaat
tgttcctttttttgtgcagTATTTCCTTTATCAGATCCTTCGTGGCTTGAAGTATATACATTCAGCAAATGTTC
TCCACCGAGACTTGAAGCCTAGCAATCTTCTTTTGAATGCAAACTGTGACCTAAAAATTTGTGA
TTTTGGGCTTGCTCGTACCACCTCAGAAACGGATTTTATGACTGAGTATGTTGTGACAAGATGG
TACAGGGCACCAGAGCTTTTGTTGAACTCCTCCGAGTACACTGCAGCAATTGATGTGTGGTCTG
TGGGCTGTATATTTATGGAACTAATGGATCGGAAACCTTTGTTTCCGGGAAGAGACCATGTCCA
TCAGCTACGTCTACTAATGGAGgttagacagtaccctatgcaacttctatatcgaattgaaatcatgttgtgcagtcctgttttagcataatagt
agttttagggctgtcatgttgaagtagctagtttgatcatagtttgttctcgaagtattaagcacttaataagtactccgtctgttcctaaatatagtctttttagagattcc
actacggactacattcagatgtatagacatactttagagtgtagattcacttattttgctccgtatgtagtccgtagtggaatcgctaaaaagacttatatttaggaac
agagggagtacttttctggtgcacattaattcattttcaattccctcatggtatttcctcgttgcagCTCATTGGAACACCAAATGAGGCTG
ATTTGGATTTTGTAAATGAAAACGCAAGAAGATATATCCGCCAACTTCCCCGTCATGCAAGGC
AATCATTATCTGAGAAGTTTCCACATGTTCACCCTTCAGCAATTGACTTGGTTGAAAAGATGCT
GACTTTCGATCCTAGACAGAGAATAACAGgcaagttttggcacgtgtttagtgatttaaccatccgcacccatttcctgatttattgttg
cttcaagacagacgatcttatcttccaaacagcaagttaaaaagagccatcctagtgtttcgttctatatatcattagctaacatcacttcatccattcaaatgaaaaa
aagagtaagtaactaaattgggaagatcacacagttaaatatatggagtatctgggctggatttacagccaagtgggtaggaatgtgtccatactctctgtaggta
tgcaaactgcattagcttgaagtgggaatgctaaagtaatcattgttgcctgttgggtggtgtttctatagttgataatccatagtttcaactgcaaaatatcggtctcc
aatattcatcctttatttttttggtcagttactgattgttcctttattttttttggtcagTTGAAGGCGCACTTGCGCATCCTTACTTGGCAT
CGCTGCATGACATAAGTGATGAGCCAGTCTGCACGATGCCCTTTAGCTTCGACTTTGAGCAGCA
TGCATTGTCGGAAGAACAAATGAAGGATCTAATCCACCAAGAGGGCATCGCGTTCAACCCTGA
TTACCAG**TAA**CTGATGTTCCTTTGTTCAGCTCCATTCACATGGAAAGTTTTCGGTCCTCCTGCCG
CCATAAAATGTCGCTAGCTGTAAATAATTGCCTCACCCGGAGAATCAAAAGGAGATGGCGTGT
TAAGGGTAGATGACAAGAGCTGTGGTGGTCAAATTTCCGCGTAGCCTATGGATTCTTGTGCTTG
TGTATGTTGTTTTATGTGGAATTTTTTTCCTTGTGCTTAAAGATGTTTCAGCATTTTTCGTAAGA
TGCGATAGTCCGTGAACGATGGCTGCCTAATTTCTGTGGCCGTCATGAGATTTTTTACATTGTG
GTCAATTATGGATTATGTAATGTTGTCCTGGTTAATGAACTCTGGCTAACTGT

Supplementary Figure S1. The transcribed portion of the genomic sequence of the *HvMPK6* gene. 7677 bp sequence of the *HvMPK6* gene was retrieved from the Golden Promise v1 genome assembly (Schreiber et al., 2020) using the nucleotide sequence of the AK376245 transcript (https://www.ncbi.nlm.nih.gov/nuccore/AK376245.1?report=fasta) as a query in GMAP search (https://ics.hutton.ac.uk/gmapper/). Exons are typed in the upper case and introns are typed in the lower case. Start and stop codon are typed in red. The location of the gRNA on-target site and the PAM site is shown with a yellow and green box, respectively. The unassembled region in the proximal end of the second intron of the gene is shown with a grey box.

```
AK376245.1          CACCACACCGCACCGCACCGCACCGCACCACCTAAACAAAACAGGAGGCGGAAAGCGACCAAATCTCGCG [  70]
BART1_0-u50115.001  ---------------------------------------------------------------------- [  70]
HORVU7Hr1G023760.2  ---------------------------------------------------------------------- [  70]
BART1_0-u50115.002  ---------------------------------------------------------------------- [  70]
HORVU7Hr1G023760.1  ---------------------------------------------------------------------- [  70]
HORVU7Hr1G023760.3  ---------------------------------------------------------------------- [  70]
HORVU7Hr1G023760.4  ---------------------------------------------------------------------- [  70]

AK376245.1          AGCGAATTCCCCTCCCCTCCCTCCCCACGCGCGCGATCCGGGCGGAGATGGACGCCGGCGGGGCGCAGCC [ 140]
BART1_0-u50115.001  ---------------------------------------------------------------------- [ 140]
HORVU7Hr1G023760.2  ---------------------------------------------------------------------- [ 140]
BART1_0-u50115.002  ---------------------------------------------------------------------- [ 140]
HORVU7Hr1G023760.1  ---------------------------------------------------------------------- [ 140]
HORVU7Hr1G023760.3  ---------------------------------------------------------------------- [ 140]
HORVU7Hr1G023760.4  ---------------------------------------------------------------------- [ 140]

AK376245.1          GCCGGACGCGGAGATGGCGGAGGCCGGGGGGGCCGCCGCGGCGGCCGCGGCCGCGGGGGCGCCGGGCGGC [ 210]
BART1_0-u50115.001  --------------------------------------------------------GGGCGCCGGGCGGC [ 210]
HORVU7Hr1G023760.2  --------------------------------------------------------GGGCGCCGGGCGGC [ 210]
BART1_0-u50115.002  ---------------------------------------------------------------------- [ 210]
HORVU7Hr1G023760.1  --------------------------------------------------------GGGCGCCGGGCGGC [ 210]
HORVU7Hr1G023760.3  ----------------------------------------------------------GCGCCGGGCGGC [ 210]
HORVU7Hr1G023760.4  ---------------------------------------------------------------------- [ 210]

AK376245.1          GCCATGGAGAACATCCAGGCCACGCTCAGCCACGGCGGGAGGTTCATCCAGTACAACATCTTTGGCAACG [ 280]
BART1_0-u50115.001  GCCATGGAGAACATCCAGGCCACGCTCAGCCACGGCGGGAGGTTCATCCAGTACAACATCTTTGGCAACG [ 280]
HORVU7Hr1G023760.2  GCCATGGAGAACATCCAGGCCACGCTCAGCCACGGCGGGAGGTTCATCCAGTACAACATCTTTGGCAACG [ 280]
BART1_0-u50115.002  -------AGAACATCCAGGCCACGCTCAGCCACGGCGGGAGGTTCATCCAGTACAACATCTTTGGCAACG [ 280]
HORVU7Hr1G023760.1  GCCATGGAGAACATCCAGGCCACGCTCAGCCACGGCGGGAGGTTCATCCAGTACAACATCTTTGGCAACG [ 280]
HORVU7Hr1G023760.3  GCCATGGAGAACATCCAGGCCACGCTCAGCCACGGCGGGAGGTTCATCCAGTACAACATCTTTGGCAACG [ 280]
HORVU7Hr1G023760.4  ---------------------------------------------------------------------- [ 280]

AK376245.1          TCTTCGAGGTCACCGCCAAGTACAAGCCCCCCATCCTCCCCATCGGCAAGGGCGCCTACGGCATCGTCTG [ 350]
BART1_0-u50115.001  TCTTCGAGGTCACCGCCAAGTACAAGCCCCCCATCCTCCCCATCGGCAAGGGCGCCTACGGCATCGTCTG [ 350]
HORVU7Hr1G023760.2  TCTTCGAGGTCACCGCCAAGTACAAGCCCCCCATCCTCCCCATCGGCAAGGGCGCCTACGGCATCGTCTG [ 350]
BART1_0-u50115.002  TCTTCGAGGTCACCGCCAAGTACAAGCCCCCCATCCTCCCCATCGGCAAGGGCGCCTACGGCATCGTCTG [ 350]
HORVU7Hr1G023760.1  TCTTCGAGGTCACCGCCAAGTACAAGCCCCCCATCCTCCCCATCGGCAAGGGCGCCTACGGCATCGTCTG [ 350]
HORVU7Hr1G023760.3  TCTTCGAGGTCACCGCCAAGTACAAGCCCCCCATCCTCCCCATCGGCAAGGGCGCCTACGGCATCGTCTG [ 350]
HORVU7Hr1G023760.4  ---------------------------------------------------------------------- [ 350]

AK376245.1          gtacgc....tgacagCTCCGCGCTCAACTCCGAGACGGGGGAGCAGGTGGCCATCAAGAAGATCGCCAA [ 488]
BART1_0-u50115.001  gtacgc....tgacagCTCCGCGCTCAACTCCGAGACGGGGGAGCAGGTGGCCATCAAGAAGATCGCCAA [ 488]
HORVU7Hr1G023760.2  gtacgc....tgacagCTCCGCGCTCAACTCCGAGACGGGGGAGCAGGTGGCCATCAAGAAGATCGCCAA [ 488]
BART1_0-u50115.002  gtacgc....tgacagctccgcgctcaactccgagACGGGGGAGCAGGTGGCCATCAAGAAGATCGCCAA [ 488]
HORVU7Hr1G023760.1  gtacgc....tgacagCTCCGCGCTCAACTCCGAGACGGGGGAGCAGGTGGCCATCAAGAAGATCGCCAA [ 488]
HORVU7Hr1G023760.3  gtacgc....tgacagCTCCGCGCTCAACTCCGAGACGGGGGAGCAGGTGGCCATCAAGAAGATCGCCAA [ 488]
HORVU7Hr1G023760.4  ---------------------------------------------------------------------- [ 488]

AK376245.1          CGCCTTCGACAACAAGATCGACGCCAAGCGCACGCTGCGGGAGATCAAGCTGCTCCGCCACATGGACCAC [ 558]
BART1_0-u50115.001  CGCCTTCGACAACAAGATCGACGCCAAGCGCACGCTGCGGGAGATCAAGCTGCTCCGCCACATGGACCAC [ 558]
HORVU7Hr1G023760.2  CGCCTTCGACAACAAGATCGACGCCAAGCGCACGCTGCGGGAGATCAAGCTGCTCCGCCACATGGACCAC [ 558]
BART1_0-u50115.002  CGCCTTCGACAACAAGATCGACGCCAAGCGCACGCTGCGGGAGATCAAGCTGCTCCGCCACATGGACCAC [ 558]
HORVU7Hr1G023760.1  CGCCTTCGACAACAAGATCGACGCCAAGCGCACGCTGCGGGAGATCAAGCTGCTCCGCCACATGGACCAC [ 558]
HORVU7Hr1G023760.3  CGCCTTCGACAACAAGATCGACGCCAAGCGCACGCTGCGGGAGATCAAGCTGCTCCGCCACATGGACCAC [ 558]
HORVU7Hr1G023760.4  ---------------------------------------------------------------------- [ 558]
```

```
                                                      ===================== Exon 3 ====================
AK376245.1            GAGAATgtcagt....atgcagATTGTTGCAATAAGGGATATTATACCTCCTGCACAAAGGACTGCATTC  [ 3908]
BART1_0-u50115.001    GAGAATgtcagt....atgcagATTGTTGCAATAAGGGATATTATACCTCCTGCACAAAGGACTGCATTC  [ 3908]
HORVU7Hr1G023760.2    GAGAATgtcagt....atgcagATTGTTGCAATAAGGGATATTATACCTCCTGCACAAAGGACTGCATTC  [ 3908]
BART1_0-u50115.002    GAGAATgtcagt....atgcagATTGTTGCAATAAGGGATATTATACCTCCTGCACAAAGGACTGCATTC  [ 3908]
HORVU7Hr1G023760.1    GAGAATgtcagt....atgcagATTGTTGCAATAAGGGATATTATACCTCCTGCACAAAGGACTGCATTC  [ 3908]
HORVU7Hr1G023760.3    GAGAATgtcagt....atgcagATTGTTGCAATAAGGGATATTATACCTCCTGCACAAAGGACTGCATTC  [ 3908]
HORVU7Hr1G023760.4    ----------------------------------------------------------------------  [ 3908]

                      =============================== Exon 3 ====  guide RNA: CTCAAATCAAGCTT
AK376245.1            AATGATGTCTATATTGCATATGAATTGATGGACACCGATCTGCATCAAATTATTCGCTCAAATCAAGCTT  [ 3978]
BART1_0-u50115.001    AATGATGTCTATATTGCATATGAATTGATGGACACCGATCTGCATCAAATTATTCGCTCAAATCAAGCTT  [ 3978]
HORVU7Hr1G023760.2    AATGATGTCTATATTGCATATGAATTGATGGACACCGATCTGCATCAAATTATTCGCTCAAATCAAGCTT  [ 3978]
BART1_0-u50115.002    AATGATGTCTATATTGCATATGAATTGATGGACACCGATCTGCATCAAATTATTCGCTCAAATCAAGCTT  [ 3978]
HORVU7Hr1G023760.1    AATGATGTCTATATTGCATATGAATTGATGGACACCGATCTGCATCAAATTATTCGCTCAAATCAAGCTT  [ 3978]
HORVU7Hr1G023760.3    AATGATGTCTATATTGCATATGAATTGATGGACACCGATCTGCATCAAATTATTCGCTCAAATCAAGCTT  [ 3978]
HORVU7Hr1G023760.4    ----------------------------------------------------------------------  [ 3978]

                      TATCGG   == Exon 3 ==
AK376245.1            TATCGGAGGAGCACTGCCAGgtactt....gtgcagTATTTCCTTTATCAGATCCTTCGTGGCTTGAAGT  [ 5855]
BART1_0-u50115.001    TATCGGAGGAGCACTGCCAGgtactt....gtgcagTATTTCCTTTATCAGATCCTTCGTGGCTTGAAGT  [ 5855]
HORVU7Hr1G023760.2    TATCGGAGGAGCACTGCCAGgtactt....gtgcagTATTTCCTTTATCAGATCCTTCGTGGCTTGAAGT  [ 5855]
BART1_0-u50115.002    TATCGGAGGAGCACTGCCAGgtactt....gtgcagTATTTCCTTTATCAGATCCTTCGTGGCTTGAAGT  [ 5855]
HORVU7Hr1G023760.1    TATCGGAGGAGCACTGCCAGgtactt....gtgcagTATTTCCTTTATCAGATCCTTCGTGGCTTGAAGT  [ 5855]
HORVU7Hr1G023760.3    TATCGGAGGAGCACTGCCAGgtactt....gtgcagTATTTCCTTTATCAGATCCTTCGTGGCTTGAAGT  [ 5855]
HORVU7Hr1G023760.4    ----------------------------------------------------------------------  [ 5855]

AK376245.1            ATATACATTCAGCAAATGTTCTCCACCGAGACTTGAAGCCTAGCAATCTTCTTTTGAATGCAAACTGTGA  [ 5925]
BART1_0-u50115.001    ATATACATTCAGCAAATGTTCTCCACCGAGACTTGAAGCCTAGCAATCTTCTTTTGAATGCAAACTGTGA  [ 5925]
HORVU7Hr1G023760.2    ATATACATTCAGCAAATGTTCTCCACCGAGACTTGAAGCCTAGCAATCTTCTTTTGAATGCAAACTGTGA  [ 5925]
BART1_0-u50115.002    ATATACATTCAGCAAATGTTCTCCACCGAGACTTGAAGCCTAGCAATCTTCTTTTGAATGCAAACTGTGA  [ 5925]
HORVU7Hr1G023760.1    ATATACATTCAGCAAATGTTCTCCACCGAGACTTGAAGCCTAGCAATCTTCTTTTGAATGCAAACTGTGA  [ 5925]
HORVU7Hr1G023760.3    ATATACATTCAGCAAATGTTCTCCACCGAGACTTGAAGCCTAGCAATCTTCTTTTGAATGCAAACTGTGA  [ 5925]
HORVU7Hr1G023760.4    ----------------------------------------------------------------------  [ 5925]

AK376245.1            CCTAAAAATTTGTGATTTTGGGCTTGCTCGTACCACCTCAGAAACGGATTTTATGACTGAGTATGTTGTG  [ 5995]
BART1_0-u50115.001    CCTAAAAATTTGTGATTTTGGGCTTGCTCGTACCACCTCAGAAACGGATTTTATGACTGAGTATGTTGTG  [ 5995]
HORVU7Hr1G023760.2    CCTAAAAATTTGTGATTTTGGGCTTGCTCGTACCACCTCAGAAACGGATTTTATGACTGAGTATGTTGTG  [ 5995]
BART1_0-u50115.002    CCTAAAAATTTGTGATTTTGGGCTTGCTCGTACCACCTCAGAAACGGATTTTATGACTGAGTATGTTGTG  [ 5995]
HORVU7Hr1G023760.1    CCTAAAAATTTGTGATTTTGGGCTTGCTCGTACCACCTCAGAAACGGATTTTATGACTGAGTATGTTGTG  [ 5995]
HORVU7Hr1G023760.3    CCTAAAAATTTGTGATTTTGGGCTTGCTCGTACCACCTCAGAAACGGATTTTATGACTGAGTATGTTGTG  [ 5995]
HORVU7Hr1G023760.4    ----------------------------------------------------------------------  [ 5995]

AK376245.1            ACAAGATGGTACAGGGCACCAGAGCTTTTGTTGAACTCCTCCGAGTACACTGCAGCAATTGATGTGTGGT  [ 6065]
BART1_0-u50115.001    ACAAGATGGTACAGGGCACCAGAGCTTTTGTTGAACTCCTCCGAGTACACTGCAGCAATTGATGTGTGGT  [ 6065]
HORVU7Hr1G023760.2    ACAAGATGGTACAGGGCACCAGAGCTTTTGTTGAACTCCTCCGAGTACACTGCAGCAATTGATGTGTGGT  [ 6065]
BART1_0-u50115.002    ACAAGATGGTACAGGGCACCAGAGCTTTTGTTGAACTCCTCCGAGTACACTGCAGCAATTGATGTGTGGT  [ 6065]
HORVU7Hr1G023760.1    ACAAGATGGTACAGGGCACCAGAGCTTTTGTTGAACTCCTCCGAGTACACTGCAGCAATTGATGTGTGGT  [ 6065]
HORVU7Hr1G023760.3    ACAAGATGGTACAGGGCACCAGAGCTTTTGTTGAACTCCTCCGAGTACACTGCAGCAATTGATGTGTGGT  [ 6065]
HORVU7Hr1G023760.4    ----------------------------------------------------------------------  [ 6065]

AK376245.1            CTGTGGGCTGTATATTTATGGAACTAATGGATCGGAAACCTTTGTTTCCGGGAAGAGACCATGTCCATCA  [ 6135]
BART1_0-u50115.001    CTGTGGGCTGTATATTTATGGAACTAATGGATCGGAAACCTTTGTTTCCGGGAAGAGACCATGTCCATCA  [ 6135]
HORVU7Hr1G023760.2    CTGTGGGCTGTATATTTATGGAACTAATGGATCGGAAACCTTTGTTTCCGGGAAGAGACCATGTCCATCA  [ 6135]
BART1_0-u50115.002    CTGTGGGCTGTATATTTATGGAACTAATGGATCGGAAACCTTTGTTTCCGGGAAGAGACCATGTCCATCA  [ 6135]
HORVU7Hr1G023760.1    CTGTGGGCTGTATATTTATGGAACTAATGGATCGGAAACCTTTGTTTCCGGGAAGAGACCATGTCCATCA  [ 6135]
HORVU7Hr1G023760.3    CTGTGGGCTGTATATTTATGGAACTAATGGATCGGAAACCTTTGTTTCCGGGAAGAGACCATGTCCATCA  [ 6135]
HORVU7Hr1G023760.4    ----------------------------------------------------------------------  [ 6135]

AK376245.1            GCTACGTCTACTAATGGAGgttaga....aagcacttaataagtactccgtctgttcctaaatatagtct  [ 6327]
BART1_0-u50115.001    GCTACGTCTACTAATGGAGgttaga....aagcacttaataagtactccgtctgttcctaaatatagtct  [ 6327]
HORVU7Hr1G023760.2    GCTACGTCTACTAATGGAGgttaga....aagcacttaataagtactccgtctgttcctaaatatagtct  [ 6327]
BART1_0-u50115.002    GCTACGTCTACTAATGGAGgttaga....aagcacttaataagtactccgtctgttcctaaatatagtct  [ 6327]
HORVU7Hr1G023760.1    GCTACGTCTACTAATGGAGgttaga....aagcacttaataagtactccgtctgttcctaaatatagtct  [ 6327]
HORVU7Hr1G023760.3    GCTACGTCTACTAATGGAGgttaga....aagcacTTAATAAGTACTCCGTCTGTTCCTAAATATAGTCT  [ 6327]
HORVU7Hr1G023760.4    ----------------------------------------------------------------------  [ 6327]
```

```
AK376245.1              ttttagagattccactacggactacattcagatgtatagacatactttagagtgtagattcacttatt.. [6395]
BART1_0-u50115.001      ttttagagattccactacggactacattcagatgtatagacatactttagagtgtagattcacttatt.. [6395]
HORVU7Hr1G023760.2      ttttagagattccactacggactacattcagatgtatagacatactttagagtgtagattcacttatt.. [6395]
BART1_0-u50115.002      ttttagagattccactacggactacattcagatgtatagacatactttagagtgtagattcacttatt.. [6395]
HORVU7Hr1G023760.1      ttttagagattccactacggactacattcagatgtatagacatactttagagtgtagATTCA-------- [6395]
HORVU7Hr1G023760.3      TTTTAGAGATTCCACTACGGACTACATTCAGATGTATAGACATACTTTAGAGTGTAGATTCA-------- [6395]
HORVU7Hr1G023760.4      ---------------------------------------------------------------------- [6395]

AK376245.1              ..ttgcagCTCATTGGAACACCAAATGAGGCTGATTTGGATTTTGTAAATGAAAACGCAAGAAGATATAT [6578]
BART1_0-u50115.001      ..ttgcagCTCATTGGAACACCAAATGAGGCTGATTTGGATTTTGTAAATGAAAACGCAAGAAGATATAT [6578]
HORVU7Hr1G023760.2      ..ttgcagCTCATTGGAACACCAAATGAGGCTGATTTGGATTTTGTAAATGAAAACGCAAGAAGATATAT [6578]
BART1_0-u50115.002      ..ttgcagCTCATTGGAACACCAAATGAGGCTGATTTGGATTTTGTAAATGAAAACGCAAGAAGATATAT [6578]
HORVU7Hr1G023760.1      ---------------------------------------------------------------------- [6578]
HORVU7Hr1G023760.3      ---------------------------------------------------------------------- [6578]
HORVU7Hr1G023760.4      ---------------------------------------------------------------------- [6578]

AK376245.1              CCGCCAACTTCCCCGTCATGCAAGGCAATCATTATCTGAGAAGTTTCCACATGTTCACCCTTCAGCAATT [6648]
BART1_0-u50115.001      CCGCCAACTTCCCCGTCATGCAAGGCAATCATTATCTGAGAAGTTTCCACATGTTCACCCTTCAGCAATT [6648]
HORVU7Hr1G023760.2      CCGCCAACTTCCCCGTCATGCAAGGCAATCATTATCTGAGAAGTTTCCACATGTTCACCCTTCAGCAATT [6648]
BART1_0-u50115.002      CCGCCAACTTCCCCGTCATGCAAGGCAATCATTATCTGAGAAGTTTCCACATGTTCACCCTTCAGCAATT [6648]
HORVU7Hr1G023760.1      ---------------------------------------------------------------------- [6648]
HORVU7Hr1G023760.3      ---------------------------------------------------------------------- [6648]
HORVU7Hr1G023760.4      ---------------------------------------------------------------------- [6648]

AK376245.1              GACTTGGTTGAAAAGATGCTGACTTTCGATCCTAGACAGAGAATAACAGgcaagt....tttggtcagtt [7113]
BART1_0-u50115.001      GACTTGGTTGAAAAGATGCTGACTTTCGATCCTAGACAGAGAATAACAGgcaagt....tttggtcagtt [7113]
HORVU7Hr1G023760.2      GACTTGGTTGAAAAGATGCTGACTTTCGATCCTAGACAGAGAATAACAGgcaagt....tttggtcagtt [7113]
BART1_0-u50115.002      GACTTGGTTGAAAAGATGCTGACTTTCGATCCTAGACAGAGAATAACAGgcaagt....tttggtcagtt [7113]
HORVU7Hr1G023760.1      ---------------------------------------------------------------------- [7113]
HORVU7Hr1G023760.3      ---------------------------------------------------------------------- [7113]
HORVU7Hr1G023760.4      -----------------------------------------------------------------CAGTT [7113]

AK376245.1              actgattgttcctttatttttttggtcagTTGAAGGCGCACTTGCGCATCCTTACTTGGCATCGCTGCA [7183]
BART1_0-u50115.001      actgattgttcctttatttttttggtcagTTGAAGGCGCACTTGCGCATCCTTACTTGGCATCGCTGCA [7183]
HORVU7Hr1G023760.2      actgattgttcctttatttttttggtcagTTGAAGGCGCACTTGCGCATCCTTACTTGGCATCGCTGCA [7183]
BART1_0-u50115.002      actgattgttcctttatttttttggtcagTTGAAGGCGCACTTGCGCATCCTTACTTGGCATCGCTGCA [7183]
HORVU7Hr1G023760.1      ---------------------------------------------------------------------- [7183]
HORVU7Hr1G023760.3      ---------------------------------------------------------------------- [7183]
HORVU7Hr1G023760.4      ACTGATTGTTCCTTTATTTTTTTGGTCAGTTGAAGGCGCACTTGCGCATCCTTACTTGGCATCGCTGCA [7183]

AK376245.1              TGACATAAGTGATGAGCCAGTCTGCACGATGCCCTTTAGCTTCGACTTTGAGCAGCATGCATTGTCGGAA [7253]
BART1_0-u50115.001      TGACATAAGTGATGAGCCAGTCTGCACGATGCCCTTTAGCTTCGACTTTGAGCAGCATGCATTGTCGGAA [7253]
HORVU7Hr1G023760.2      TGACATAAGTGATGAGCCAGTCTGCACGATGCCCTTTAGCTTCGACTTTGAGCAGCATGCATTGTCGGAA [7253]
BART1_0-u50115.002      TGACATAAGTGATGAGCCAGTCTGCACGATGCCCTTTAGCTTCGACTTTGAGCAGCATGCATTGTCGGAA [7253]
HORVU7Hr1G023760.1      ---------------------------------------------------------------------- [7253]
HORVU7Hr1G023760.3      ---------------------------------------------------------------------- [7253]
HORVU7Hr1G023760.4      TGACATAAGTGATGAGCCAGTCTGCACGATGCCCTTTAGCTTCGACTTTGAGCAGCATGCATTGTCGGAA [7253]

AK376245.1              GAACAAATGAAGGATCTAATCCACCAAGAGGGCATCGCGTTCAACCCTGATTACCAGTAACTGATGTTCC [7323]
BART1_0-u50115.001      GAACAAATGAAGGATCTAATCCACCAAGAGGGCATCGCGTTCAACCCTGATTACCAGTAACTGATGTTCC [7323]
HORVU7Hr1G023760.2      GAACAAATGAAGGATCTAATCCACCAAGAGGGCATCGCGTTCAACCCTGATTACCAGTAACTGATGTTCC [7323]
BART1_0-u50115.002      GAACAAATGAAGGATCTAATCCACCAAGAGGGCATCGCGTTCAACCCTGATTACCAGTAACTGATGTTCC [7323]
HORVU7Hr1G023760.1      ---------------------------------------------------------------------- [7323]
HORVU7Hr1G023760.3      ---------------------------------------------------------------------- [7323]
HORVU7Hr1G023760.4      GAACAAATGAAGGATCTAATCCACCAAGAGGGCATCGCGTTCAACCCTGATTACCAGTAActgatgttcc [7323]

AK376245.1              TTTGTTCAGCTCCATTCACATGGAAAGTTTTCGGTCCTCCTGCCGCCATAAAATGTCGCTAGCTGTAAAT [7393]
BART1_0-u50115.001      TTTGTTCAGCTCCATTCACATGGAAAGTTTTCGGTCCTCCTGCCGCCATAAAATGTCGCTAGCTGTAAAT [7393]
HORVU7Hr1G023760.2      TTTGTTCAGCTCCATTCACATGGAAAGTTTTCGGTCCTCCTGCCGCCATAAAATGTCGCTAGCTGTAAAT [7393]
BART1_0-u50115.002      TTTGTTCAGCTCCATTCACATGGAAAGTTTTCGGTCCTCCTGCCGCCATAAAATGTCGCTAGCTGTAAAT [7393]
HORVU7Hr1G023760.1      ---------------------------------------------------------------------- [7393]
HORVU7Hr1G023760.3      ---------------------------------------------------------------------- [7393]
HORVU7Hr1G023760.4      ttgttcagctccattcacatggaaagttttcggtcctcctgccgccataaaatgtcgctagctgtaaat [7393]
```

```
AK376245.1          AATTGCCTCACCCGGAGAATCAAAAGGAGATGGCGTGTTAAGGGTAGATGACAAGAGCTGTGGTGGTCAA [7463]
BART1_0-u50115.001  AATTGCCTCACCCGGAGAATCAAAAGGAGATGGCGTGTTAAGGGTAGATGACAAGAGCTGTGGTGGTCAA [7463]
HORVU7Hr1G023760.2  AATTGCCTCACCCGGAGAATCAAAAGGAGATGGCGTGTTAAGGGTAGATGACAAGAGCTGTGGTGGTCAA [7463]
BART1_0-u50115.002  AATTGCCTCACCCGGAGAATCAAAAGGAGATGGCGTGTTAAGGGTAGATGACAAGAGCTGTGGTGGTCAA [7463]
HORVU7Hr1G023760.1  ---------------------------------------------------------------------- [7463]
HORVU7Hr1G023760.3  ---------------------------------------------------------------------- [7463]
HORVU7Hr1G023760.4  aattgcctcacccggagaatcaaaaggagatggcgtgttaagggtagatgacaagagctgtggtggtcaa [7463]

AK376245.1          ATTTCCGCGTAGCCTATGGATTCTTGTGCTTGTGTATGTTGTTTTATGTGGAATTTTTTTCCTTGTGCTT [7533]
BART1_0-u50115.001  ATTTCCGCGTAGCCTATGGATTCTTGTGCTTGTGTATGTTGTTTTATGTGGAATTTTTTTCCTTGTGCTT [7533]
HORVU7Hr1G023760.2  ATTTCCGCGTAGCCTATGGATTCTTGTGCTTGTGTATGTTGTTTTATGTGGAATTTTTTTCCTTGTGCTT [7533]
BART1_0-u50115.002  ATTTCCGCGTAGCCTATGGATTCTTGTGCTTGTGTATGTTGTTTTATGTGGAATTTTTTTCCTTGTGCTT [7533]
HORVU7Hr1G023760.1  ---------------------------------------------------------------------- [7533]
HORVU7Hr1G023760.3  ---------------------------------------------------------------------- [7533]
HORVU7Hr1G023760.4  atttccgcgtagcctatggattcttgtgcttgtgtatgttgttttatgtggaatttttttccttgtgctt [7533]


AK376245.1          AAAGATGTTTCAGCATTTTTCGTAAGATGCGATAGTCCGTGAACGATGGCTGCCTAATTTCTGTGGCCGT [7603]
BART1_0-u50115.001  AAAGATGTTTCAGCATTTTTCGTAAGATGCGATAGTCCGTGAACGATGGCTGCCTAATTTCTGTGGCCGT [7603]
HORVU7Hr1G023760.2  AAAGATGTTTCAGCATTTTTCGTAAGATGCGATAGTCCGTGAACGATGGCTGCCTAATTTCTGTGGCCGT [7603]
BART1_0-u50115.002  AAAGATGTTTCAGCATTTTTCGTAAGATGCGATAGTCCGTGAACGATGGCTGCCTAATTTCTGTGGCCGT [7603]
HORVU7Hr1G023760.1  ---------------------------------------------------------------------- [7603]
HORVU7Hr1G023760.3  ---------------------------------------------------------------------- [7603]
HORVU7Hr1G023760.4  aaagatgtttcagcatttttcgtaagatgcgatagtccgtgaacgatggctgcctaatttctgtggccgt [7603]

AK376245.1          CATGAGATTTTTTACATTGTGGTCAATTATGGATTATGTAATGTTGTCCTGGTTAATGAACTCTGGCTAA [7673]
BART1_0-u50115.001  CATGAGATTTTTTACATTGTGGTCAATTATGGATTATGTAATGTTGTCCTGGTTAATGAACTCTGGCTAA [7673]
HORVU7Hr1G023760.2  CATGAGATTTTTTACATTGTGGTCAATTATGGATTATGTAATGTTGTCCTGGTTAATGAACTCTGGC--- [7673]
BART1_0-u50115.002  CATGAGATTTTTTACATTGTGGTCAATTATGGATTATGTAATGTTGTCCTGGTTAATGAACTCTGGCTAA [7673]
HORVU7Hr1G023760.1  ---------------------------------------------------------------------- [7673]
HORVU7Hr1G023760.3  ---------------------------------------------------------------------- [7673]
HORVU7Hr1G023760.4  catgagatttttttacattgtggtcaattatggattatgtaatgttgtcctggttaatgaactctggc--- [7673]

AK376245.1          CTGT [7677]
BART1_0-u50115.001  CTGT [7677]
HORVU7Hr1G023760.2  ---- [7677]
BART1_0-u50115.002  CTGT [7677]
HORVU7Hr1G023760.1  ---- [7677]
HORVU7Hr1G023760.3  ---- [7677]
HORVU7Hr1G023760.4  ---- [7677]
```

Supplementary Figure S2. Comparison of the transcripts associated with the *HvMPK6* gene. Splicing variants of the *HvMPK6 HORVU7Hr1G023760* gene (EnsemblPlants Hordeum vulgare IBSC_v2 genome assembly; Mascher et al., 2017; https://plants.ensembl.org/index.html), *HvMPK6* BART1_0-u50115.001 and BART1_0-u50115.00 transcripts (Barley Reference Transcript Dataset BaRTv1.0; Rapazote-Flores et al., 2019; https://ics.hutton.ac.uk/barleyrtd/blast_page.html) and AK376245.1 transcript (https://www.ncbi.nlm.nih.gov/nuccore/AK376245.1?report=fasta) are presented in the transcript comparison view. Exons are shown in the upper case and introns are shown in the lower case. The grey box indicates 5′ and 3′ untranslated region, the turquoise box indicates coding exon or coding portion of the exon and the yellow box indicates introns. Where applicable, introns were shortened (indicated with four dots) to maintain clarity of the alignment. Transcript designation is shown on the left and the position of the last nucleotide on the right of each row of a nucleotide sequence. The position of the last nucleotide is of AK376245.1 transcript and considers unshortened introns. Start and stop codon are typed in red and underlined. The position of the gRNA on-target site and the PAM site in the exon 3 (Exon 3) is shown in bolt typing and the PAM site is also underlined. 20bp gRNA sequence is shown in red typing above the on-target site. All transcript variants, except for *HORVU7Hr1G023760.4*, are targeted by the designed gRNA.

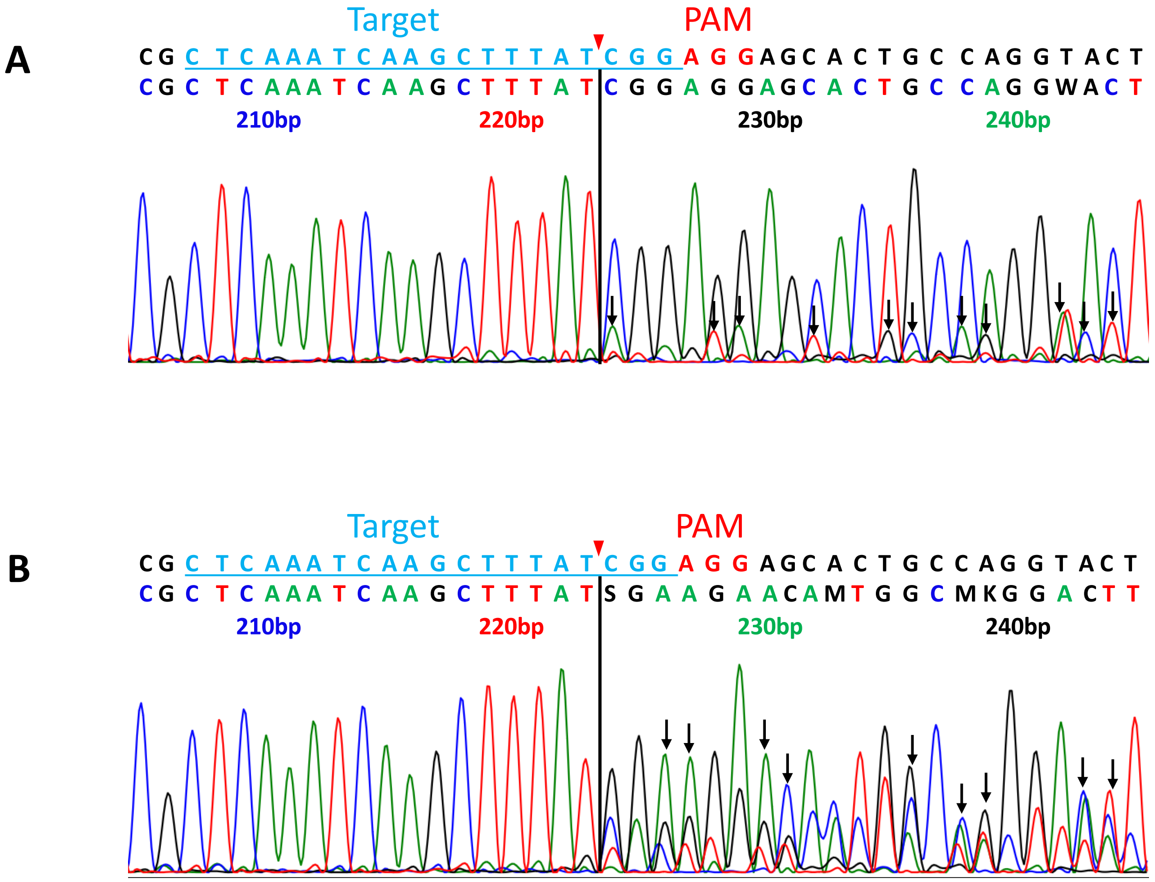

Supplementary Figure S3. Superimposed sequencing chromatograms associated with plants chimeric in the induced mutations. Above the chromatograms, deduced colour-coded nucleotide sequence and reference WT sequence including the position of the on-target site (underlined lightly blue typing) and the PAM site (red typing) are shown. The nucleotide numbering is presented as the distance in base pairs (bp) from the first nucleotide of the K6E3_F1 primer site. Peaks start to overlap at the third nucleotide position upstream of the PAM site (indicated by vertical solid line). Cas9 endonuclease from *S. pyogenes* cleaves target DNA sequences most frequently after the third nucleotide upstream of a PAM site (Jinek et al., 2012; Ran et al., 2015) (red arrowhead). The vertical solid line and red arrowhead point to the same position suggesting that the on-target was cleaved at the expected site and that the mutation/ mutations occurred next to the cleavage site in the downstream direction relative to the PAM site. In **(A)**, two chromatograms with different signal intensity overlap. Major chromatogram corresponds to the WT *HvMPK6* allele because the nearly complete deduced sequence above the superimposed region is of a WT type. Minor chromatogram (peaks indicated with an arrow) corresponds to the mutated *Hvmpk6* allele. The individual is a chimaera comprised more of WT cells than of mutated cells. In **(B)**, three chromatograms overlap. Each overlap of three peaks is indicated with an arrow. The individual is a multiple genotype chimera of a WT *HvMPK6* allele and two different mutated *Hvmpk6* alleles.

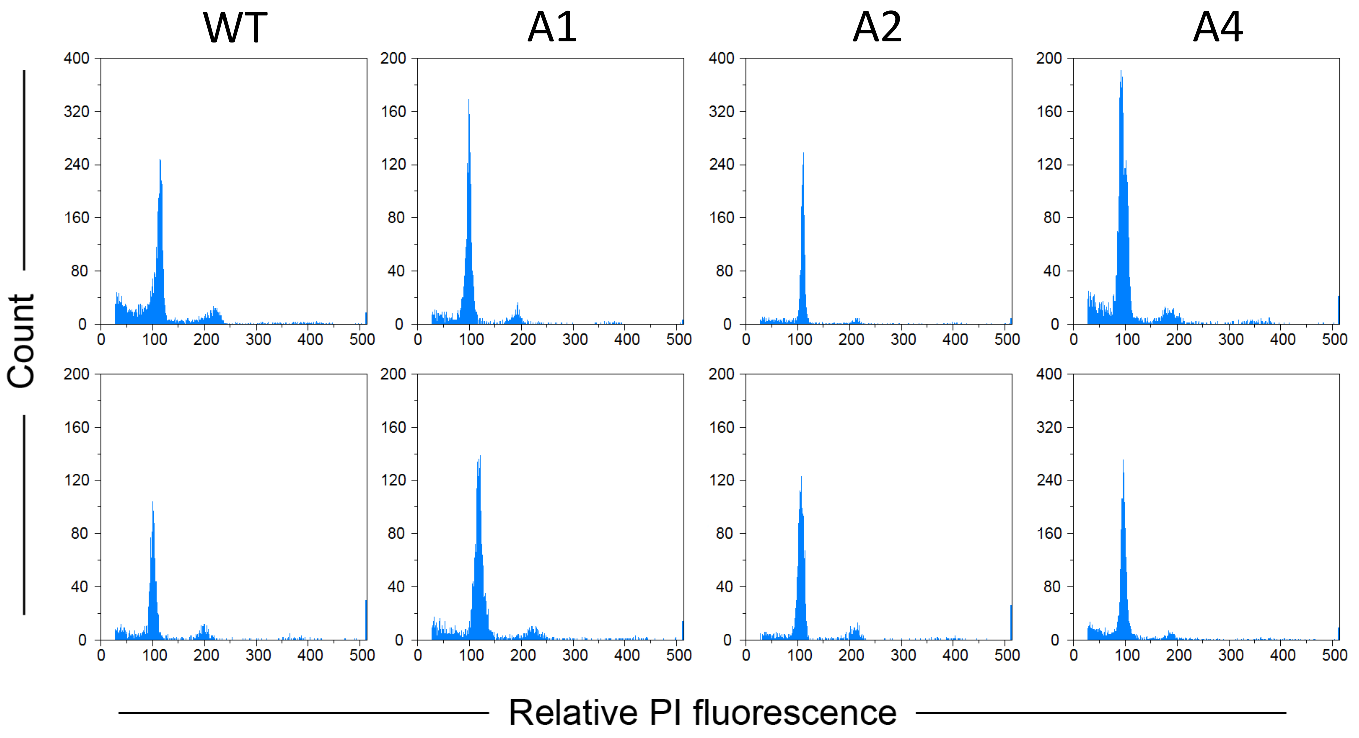

Supplementary Figure S4. Ploidy estimation of the transgenic line A. Flow cytometric analysis of propidium iodide-stained nuclei isolated from fresh leaves. Wild-type Golden Promise barley was used as an external standard, its G1 peak was positioned on channel 100. All three investigated T2 generation transgenic plants (A1, A2 and A4) were of the same ploidy level as standard, i.e. diploid.

```
HvMPK6             MDAGGAQPPDAEMAEAGGAAAAAAAAGAPGGAMENIQATLSHGGRFIQYNIFGNVFEVTAKYKPPILPIGKGAYGIVCSA  80
-3ATC              MDAGGAQPPDAEMAEAGGAAAAAAAAGAPGGAMENIQATLSHGGRFIQYNIFGNVFEVTAKYKPPILPIGKGAYGIVCSA  80
-1C                MDAGGAQPPDAEMAEAGGAAAAAAAAGAPGGAMENIQATLSHGGRFIQYNIFGNVFEVTAKYKPPILPIGKGAYGIVCSA  80
-25 bp             MDAGGAQPPDAEMAEAGGAAAAAAAAGAPGGAMENIQATLSHGGRFIQYNIFGNVFEVTAKYKPPILPIGKGAYGIVCSA  80
-18 bp + 8 bp      MDAGGAQPPDAEMAEAGGAAAAAAAAGAPGGAMENIQATLSHGGRFIQYNIFGNVFEVTAKYKPPILPIGKGAYGIVCSA  80
-19 bp             MDAGGAQPPDAEMAEAGGAAAAAAAAGAPGGAMENIQATLSHGGRFIQYNIFGNVFEVTAKYKPPILPIGKGAYGIVCSA  80
-20 bp             MDAGGAQPPDAEMAEAGGAAAAAAAAGAPGGAMENIQATLSHGGRFIQYNIFGNVFEVTAKYKPPILPIGKGAYGIVCSA  80
-23 bp             MDAGGAQPPDAEMAEAGGAAAAAAAAGAPGGAMENIQATLSHGGRFIQYNIFGNVFEVTAKYKPPILPIGKGAYGIVCSA  80
+4 bp +6 bp, C-T   MDAGGAQPPDAEMAEAGGAAAAAAAAGAPGGAMENIQATLSHGGRFIQYNIFGNVFEVTAKYKPPILPIGKGAYGIVCSA  80
-1 bp complex      MDAGGAQPPDAEMAEAGGAAAAAAAAGAPGGAMENIQATLSHGGRFIQYNIFGNVFEVTAKYKPPILPIGKGAYGIVCSA  80

HvMPK6             LNSETGEQVAIKKIANAFDNKIDAKRTLREIKLLRHMDHENIVAIRDIIPPAQRTAFNDVYIAYELMDTDLHQIIRSNQA  160
-3ATC              LNSETGEQVAIKKIANAFDNKIDAKRTLREIKLLRHMDHENIVAIRDIIPPAQRTAFNDVYIAYELMDTDLHQIIRSNQA  160
-1C                LNSETGEQVAIKKIANAFDNKIDAKRTLREIKLLRHMDHENIVAIRDIIPPAQRTAFNDVYIAYELMDTDLHQIIRSNQA  160
-25 bp             LNSETGEQVAIKKIANAFDNKIDAKRTLREIKLLRHMDHENIVAIRDIIPPAQRTAFNDVYIAYELMDTDLHQIIRSNQA  160
-18bp + 8bp        LNSETGEQVAIKKIANAFDNKIDAKRTLREIKLLRHMDHENIVAIRDIIPPAQRTAFNDVYIAYELMDTDLHQIIRSNQA  160
-19 bp             LNSETGEQVAIKKIANAFDNKIDAKRTLREIKLLRHMDHENIVAIRDIIPPAQRTAFNDVYIAYELMDTDLHQIIRRSTA  160
-20 bp             LNSETGEQVAIKKIANAFDNKIDAKRTLREIKLLRHMDHENIVAIRDIIPPAQRTAFNDVYIAYELMDTDLHQIIGGALP  160
-23 bp             LNSETGEQVAIKKIANAFDNKIDAKRTLREIKLLRHMDHENIVAIRDIIPPAQRTAFNDVYIAYELMDTDLHQIIRSNQG  160
+4 bp +6 bp, C-T   LNSETGEQVAIKKIANAFDNKIDAKRTLREIKLLRHMDHENIVAIRDIIPPAQRTAFNDVYIAYELMDTDLHQIIRSNQA  160
-1 bp complex      LNSETGEQVAIKKIANAFDNKIDAKRTLREIKLLRHMDHENIVAIRDIIPPAQRTAFNDVYIAYELMDTDLHQIIRSNQA  160

HvMPK6             LSEEHCQYFLYQILRGLKYIHSANVLHRDLKPSNLLLNANCDLKICDFGLARTTSETDFMTEYVVTRWYRAPELLLNSSE  240
-3ATC              L-EEHCQYFLYQILRGLKYIHSANVLHRDLKPSNLLLNANCDLKICDFGLARTTSETDFMTEYVVTRWYRAPELLLNSSE  239
-1C                LWRSTASISFIRSFVA*---------------------------------------------------------------  177
-25 bp             LLVTSI*-------------------------------------------------------------------------  167
-18bp + 8bp        LVLISYFDTPL*--------------------------------------------------------------------  172
-19 bp             SISFIRSFVA*---------------------------------------------------------------------  171
-20 bp             VFPLSDPSWLEVYTFSKCSPPRLEA*------------------------------------------------------  186
-23 bp             TY*-----------------------------------------------------------------------------  163
+4 bp +6 bp, C-T   SHTPFGGALPVFPLSDPSWLEVYTFSKCSPPRLEA*--------------------------------------------  196
-1 bp complex      LVTSI*--------------------------------------------------------------------------  166

HvMPK6             YTAAIDVWSVGCIFMELMDRKPLFPGRDHVHQLRLLMELIGTPNEADLDFVNENARRYIRQLPRHARQSLSEKFPHVHPS  320
-3ATC              YTAAIDVWSVGCIFMELMDRKPLFPGRDHVHQLRLLMELIGTPNEADLDFVNENARRYIRQLPRHARQSLSEKFPHVHPS  319
-1C                --------------------------------------------------------------------------------
-25 bp             --------------------------------------------------------------------------------
-18bp + 8bp        --------------------------------------------------------------------------------
-19 bp             --------------------------------------------------------------------------------
-20 bp             --------------------------------------------------------------------------------
-23 bp             --------------------------------------------------------------------------------
+4 bp +6 bp, C-T   --------------------------------------------------------------------------------
-1 bp complex      --------------------------------------------------------------------------------

HvMPK6             AIDLVEKMLTFDPRQRITVEGALAHPYLASLHDISDEPVCTMPFSFDFEQHALSEEQMKDLIHQEGIAFNPDY*  394
-3ATC              AIDLVEKMLTFDPRQRITVEGALAHPYLASLHDISDEPVCTMPFSFDFEQHALSEEQMKDLIHQEGIAFNPDY*  393
-1C                ---------------------------------------------------------------------------
-25 bp             ---------------------------------------------------------------------------
-18bp + 8bp        ---------------------------------------------------------------------------
-19 bp             ---------------------------------------------------------------------------
-20 bp             ---------------------------------------------------------------------------
-23 bp             ---------------------------------------------------------------------------
+4 bp +6 bp, C-T   ---------------------------------------------------------------------------
-1 bp complex      ---------------------------------------------------------------------------
```

Supplementary Figure S5. Alignment of the HvMPK6 amino acid sequence with the amino acid sequences of putative proteins resulting from the CRISPR/Cas9-edited *HvMPK6*. The mutations of T2 generation A1 and A2 plants (Figure 4) were individually introduced into the reference AK376245.1.mrna1_gpv1_chr7H_26521825_26529501_0 *HvMPK6* mRNA nucleotide sequence *in silico* using ApE plasmid editor (https://jorgensen.biology.utah.edu/wayned/ape/). The reference and aberrant amino acid sequences were translated from the reference and edited nucleotide sequences, respectively, in ApE and aligned using COBALT (https://www.ncbi.nlm.nih.gov/tools/cobalt/cobalt.cgi). The turquoise box indicates the native amino acid sequence and the red box indicates the aberrant amino acid sequence resulting from frameshift translation. The asterisk in black bold typing and the asterisk indicate premature and native stop of translation, respectively. The yellow box indicates a dual TEY phosphorylation motif and the dash indicates an absence of amino acid. Designation of the reference or respective mutation is shown on the left and the position of the last amino acid on the right of each row. Designation of the prevalent -1C mutation is shown in the green box and designation of the mutations, which eliminated the splicing site between the exon 3 and the intron 4 are shown in the grey box. In the case of the last mutations, the amino acid sequences were translated from mutated nucleotide sequences including the respective portion of the intron 4.

```
Reference                GATCTGCATCAAATTATTCGCTCAAATCAAGCTTTATCGGAGGAGCACTGCCAG/GTACTTATTAG
WT allele                GATCTGCATCAAATTATTCGCTCAAATCAAGCTTTATCGGAGGAGCACTGCCAG/GTACTTATTAG  16X
-1T allele               GATCTGCATCAAATTATTCGCTCAAATCAAGCTTTA-CGGAGGAGCACTGCCAG/GTACTTATTAG   1X
-2AT allele              GATCTGCATCAAATTATTCGCTCAAATCAAGCTTT--CGGAGGAGCACTGCCAG/GTACTTATTAG   2X
-3TAT allele             GATCTGCATCAAATTATTCGCTCAAATCAAGCTT---CGGAGGAGCACTGCCAG/GTACTTATTAG   1X
-4 bp, C-T allele        GATCTGCATCAAATTATTCGCTCAAATCAA----TATTGGAGGAGCACTGCCAG/GTACTTATTAG   1X
-9 bp allele             GATCTGCATCAAATTATTCGCTCAAATCAAGCTT---------AGCACTGCCAG/GTACTTATTAG   1X
-20 bp allele            GATCTGCATCAAATTAT--------------------CGGAGGAGCACTGCCAG/GTACTTATTAG   2X
-25 bp allele            GATCTGCATCAAATTATTCGCTCAAATCAAGCTTTAT-----------------/--------TAG   2X
C-T allele*              GATCTGCATCAAATTATTCGCTCAAATCAAGCTTTATTGGAGGAGCACTGCCAG/GTACTTATTAG   1X
15 bp subst. allele      GATCTGCATCAAATTATTCGCTCAAATCAAGCTTTGATATAGAAGTAAAACCAG/GTACTTATTAG   1X
23 bp subst. allele      GATCTGCATCAAATTATTCGCTCAAATCAAGCTTTATAGAAGTAGACCCTTCTC/ATACCCATTAG   1X

Reference                CATCAAATTATTCGCTCAAATCAAGCTTTAT------CGGAGGAGCACTGCCAG/GTACTTATTAG
+6 bp, C-T allele        CATCAAATTATTCGCTCAAATCAAGCTTTATAGTTATTGGAGGAGCACTGCCAG/GTACTTATTAG   1X

Reference                CGCTCAAATCAAGCTTTAT----------------C-----GGAGGAGCACTGCCAG/GTACTTAT
+16 bp, C, +5 bp allele  CGCTCAAATCAAGCTTTATTAAGACCTTATATGAACTGTATGGAGGAGCACTGCCAG/GTACTTAT   1X

Reference                GATCTGCATCAAATTATTCGCTCAAATCAAGCTTTATCGGAGGAGCACTG----CCAG-/GTACTT
+4 bp, complex allele    GATCTGCATCAAATTATTCGCTCAAATCAAGCTTTATTAG-TTACTTCTGATTCCCCGA/GTACTT   1X
```

Supplementary Figure S6. *HvMPK6* alleles of the T2 progeny plants of a chimeric A4 plant. Genomics DNA for the mutation analysis was extracted from the leaves of emerged transgenic plants. Superimposed sequencing chromatograms were decoded into allelic sequences using the DSDecodeM tool, cloning of the 316 bp PCR amplicons and occasionally manual decoding. The selected region of the WT *HvMPK6* allele is shown as a reference on the top of each multiple sequence alignment with the position of the gRNA on-target site and the PAM site highlighted in yellow and green, respectively. Slash indicates exon/intron boundary and dash indicates single nucleotide deletion. Inserted or changed nucleotides are typed in red. Allele designation is shown on the left of each sequence. Minus indicates deletion, plus indicates insertion and bp indicates base pair. The frequency of the *HvMPK6* alleles is shown on the right of each sequence. In total, 17 mutated A4 plants were identified, 15 of them were heterozygous mutants and two were chimaeras comprised more of WT cells than of mutated cells. All mutated plants contained the WT *HvMPK6* allele. The allele of one chimeric plant is indicated with asterisks. WT and mutant allele of the second chimeric plant are not presented, because the sequence of the mutated allele was not decoded.

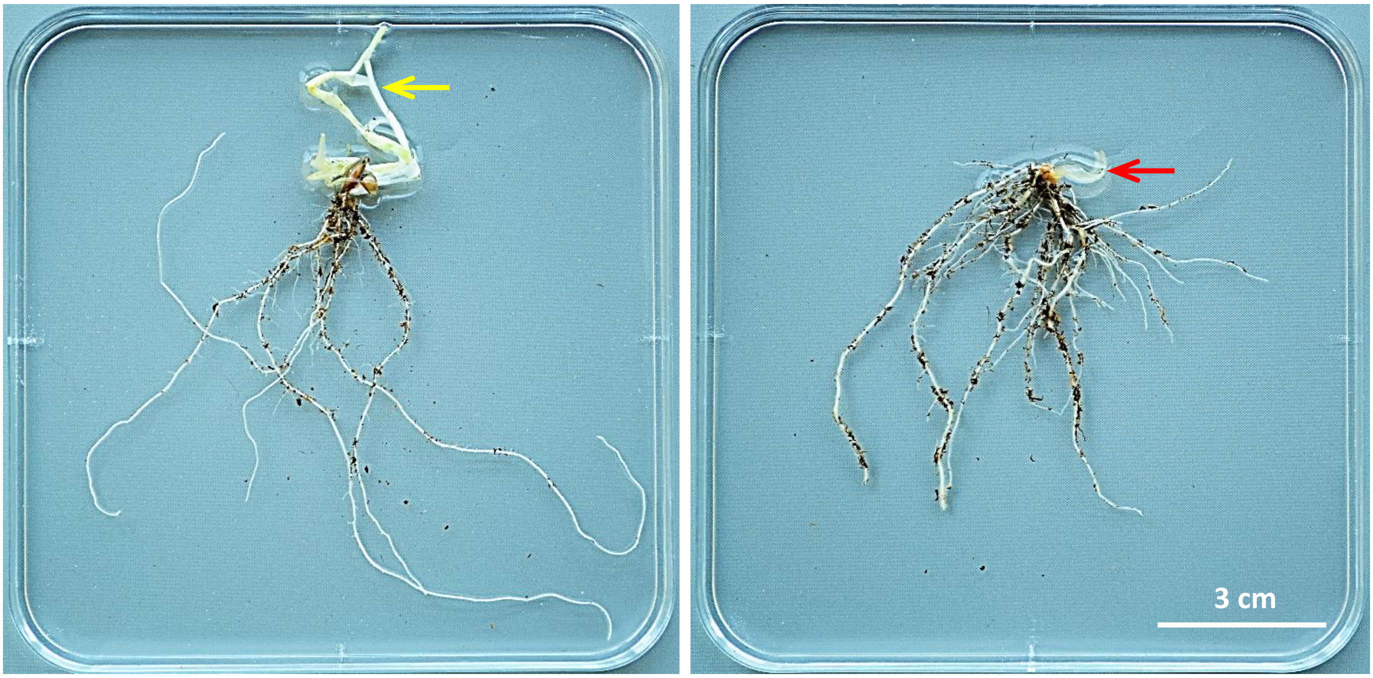

transgenic WT *Hvmpk6* mutant (-1C/-1C)

Supplementary Figure S7. Phenotypic comparison of the abnormal transgenic WT and *Hvmpk6* mutant (-1C/-1C) seedlings. Seedlings were removed from the soil of pots with no plant emergence three weeks after sowing the T3 generation grains of heterozygous mutant (WT/-1C) plants. Abnormal WT seedling developed aberrant but obvious shoot, which failed to emerge (indicated with a yellow arrow) whereas *Hvmpk6* mutant (-1C/-1C) seedling is shootless and developed only a small chlorotic leaf blade-like structure (a red arrow).

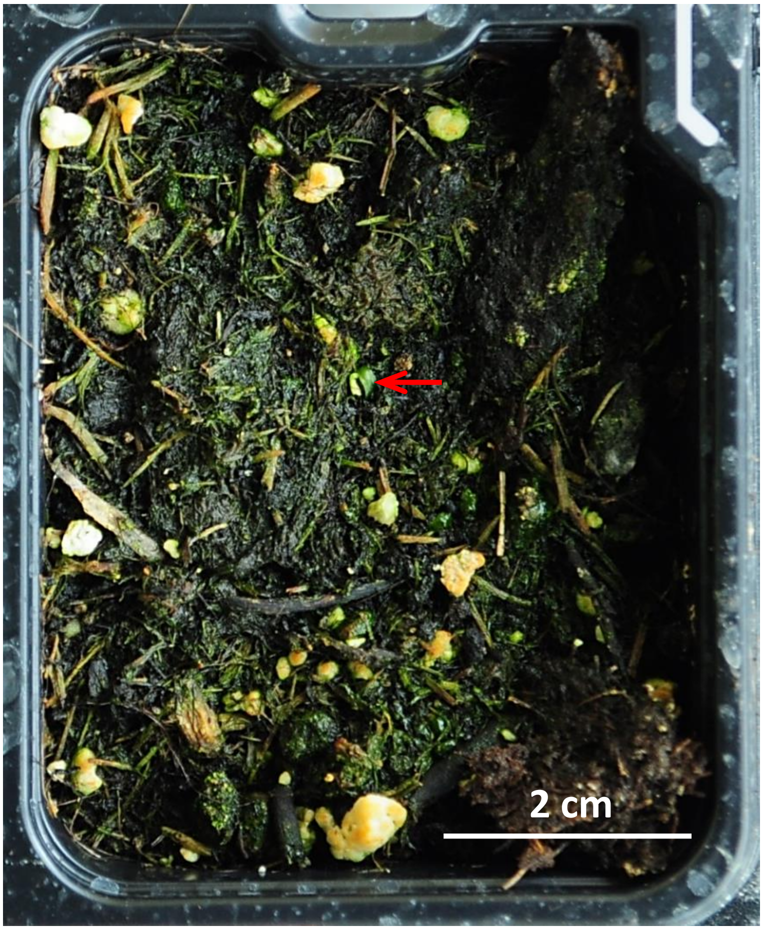

Supplementary Figure S8. A rare example of the abnormal seedling heterozygous in the *HvMPK6* gene (WT/-1C), which, early after sowing, shoved some signs of shoot emergence. Photography was taken three weeks after the sowing of T3 generation grains of heterozygous mutant (WT/-1C) plants. The emerging shoot part is indicated with the red arrow. Following 31 days after sowing, the seedling did not show any additional signs of emergence. The remaining roots were removed from the soil 59 days after sowing and used for PCR-RE and PCR-Seq genotyping of the plant.

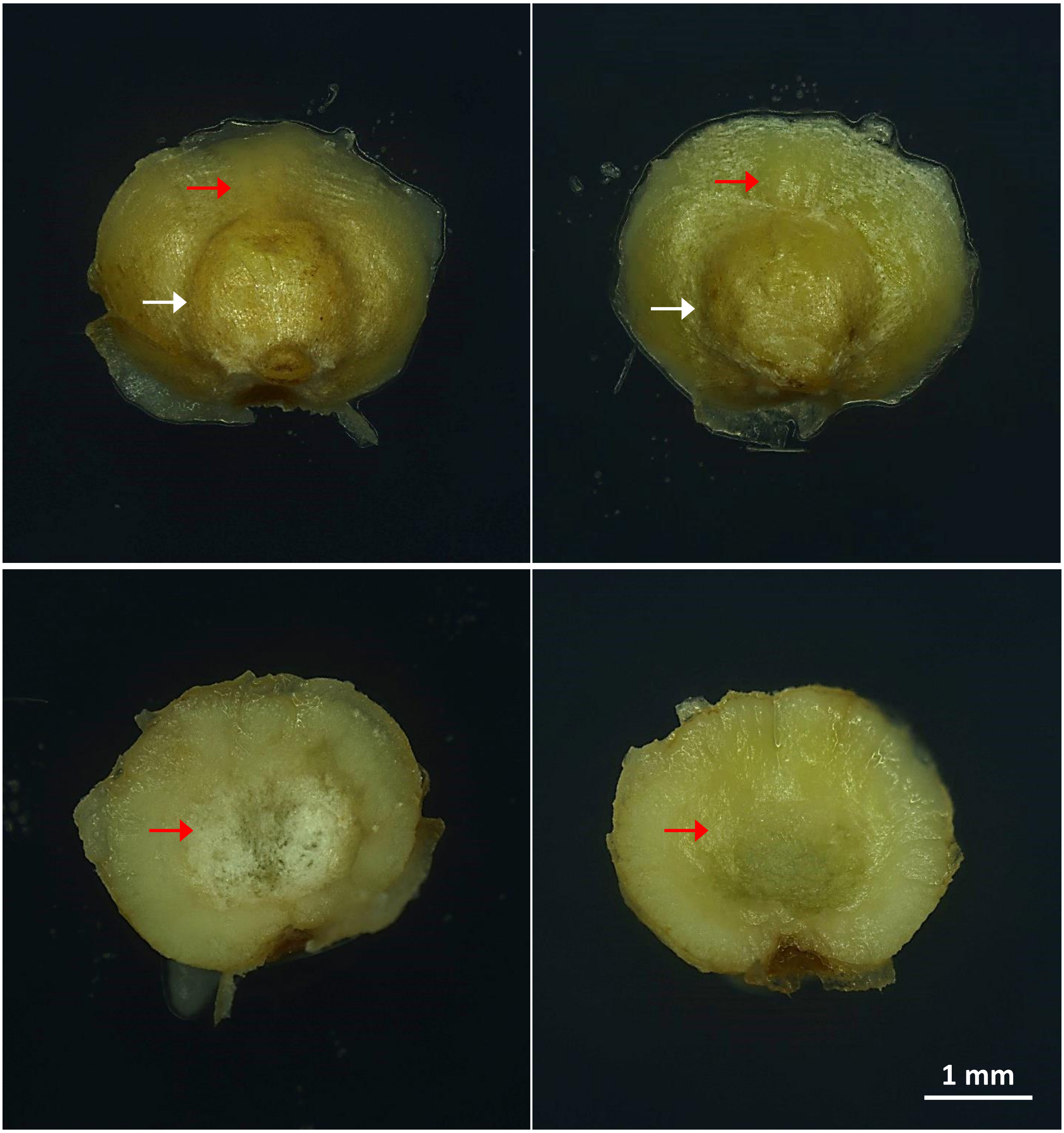

Supplementary Figure S9. Examples of the *Hvmpk6* mutant (-1C/-1C) embryos with hints of the shoot part of the embryonic axis. Mature T3 generation grains of heterozygous mutant (WT/-1C) plants were imbibed in Milli-Q water at 21°C in darkness for 18 hours before embryo extirpation and imaging. The upper line of the images – an adaxial site of the extirpated embryos. The white arrow indicates the root part of the embryonic axis and the red arrow indicates hints of the shoot part of the axis. The lower line of the images – an abaxial site of the extirpated embryos. The red arrow indicates the hollow shape of the abaxial embryo site.

```
Score                   Identities              Positives               Gaps
682 bits(1760)          337/396(85%)            362/396(91%)            4/396(1%)

HvMPK6  1    MDAGGAQPP-DAEMAEA-GGAAAAAAAAGAPGGAMENIQATLSHGGRFIQYNIFGNVFEV  58
             MD G  QP  D EM EA GG  AAA +    PG  +ENI ATLSHGGRFIQYNIFGN+FEV
AtMPK6  1    MDGGSGQPAADTEMTEAPGGFPAAAPSPQMPG--IENIPATLSHGGRFIQYNIFGNIFEV  58

HvMPK6  59   TAKYKPPILPIGKGAYGIVCSALNSETGEQVAIKKIANAFDNKIDAKRTLREIKLLRHMD  118
             TAKYKPPI+PIGKGAYGIVCSA+NSET E VAIKKIANAFDNKIDAKRTLREIKLLRHMD
AtMPK6  59   TAKYKPPIMPIGKGAYGIVCSAMNSETNESVAIKKIANAFDNKIDAKRTLREIKLLRHMD  118

HvMPK6  119  HENIVAIRDIIPPAQRTAFNDVYIAYELMDTDLHQIIRSNQALSEEHCQYFLYQILRGLK  178
             HENIVAIRDIIPP  R AFNDVYIAYELMDTDLHQIIRSNQALSEEHCQYFLYQILRGLK
AtMPK6  119  HENIVAIRDIIPPPLRNAFNDVYIAYELMDTDLHQIIRSNQALSEEHCQYFLYQILRGLK  178

HvMPK6  179  YIHSANVLHRDLKPSNLLLNANCDLKICDFGLARTTSETDFMTEYVVTRWYRAPELLLNS  238
             YIHSANVLHRDLKPSNLLLNANCDLKICDFGLAR TSE+DFMTEYVVTRWYRAPELLLNS
AtMPK6  179  YIHSANVLHRDLKPSNLLLNANCDLKICDFGLARVTSESDFMTEYVVTRWYRAPELLLNS  238

HvMPK6  239  SEYTAAIDVWSVGCIFMELMDRKPLFPGRDHVHQLRLLMELIGTPNEADLDFVNENARRY  298
             S+YTAAIDVWSVGCIFMELMDRKPLFPGRDHVHQLRLLMELIGTP+E +L+F+NENA+RY
AtMPK6  239  SDYTAAIDVWSVGCIFMELMDRKPLFPGRDHVHQLRLLMELIGTPSEEELEFLNENAKRY  298

HvMPK6  299  IRQLPRHARQSLSEKFPHVHPSAIDLVEKMLTFDPRQRITVEGALAHPYLASLHDISDEP  358
             IRQLP + RQS+++KFP VHP AIDL+EKMLTFDPR+RITV  ALAHPYL SLHDISDEP
AtMPK6  299  IRQLPPYPRQSITDKFPTVHPLAIDLIEKMLTFDPRRRITVLDALAHPYLNSLHDISDEP  358

HvMPK6  359  VCTMPFSFDFEQHALSEEQMKDLIHQEGIAFNPDYQ  394
              CT+PF+FDFE HALSEEQMK+LI++E +AFNP+YQ
AtMPK6  359  ECTIPFNFDFENHALSEEQMKELIYREALAFNPEYQQ 395
```

Supplementary Figure S10. Alignment of the barley MPK6 and Arabidopsis MPK6 amino acid sequences. Predicted AK376245.1.mrna1_gpv1_chr7H_26521825_26529501_0 HvMPK6 amino acid sequence and AtMPK6 amino acid sequence (https://www.ncbi.nlm.nih.gov/protein/OAP09870.1) were compared using protein-protein BLAST utility of NCBI (https://blast.ncbi.nlm.nih.gov/Blast.cgi). Parameters of the amino acid sequence comparison are shown above the alignment. HvMPK6 is a 394 amino acid long protein and AtMPK6 is a 395 amino acid long protein. The C-terminal epitope of AtMPK6, against which anti-AtMPK6 antibody was commercially raised and corresponding HvMPK6 epitope are shown with yellow boxes. Identical and similar (+) amino acids shared by these two epitopes are shown with turquoise boxes.

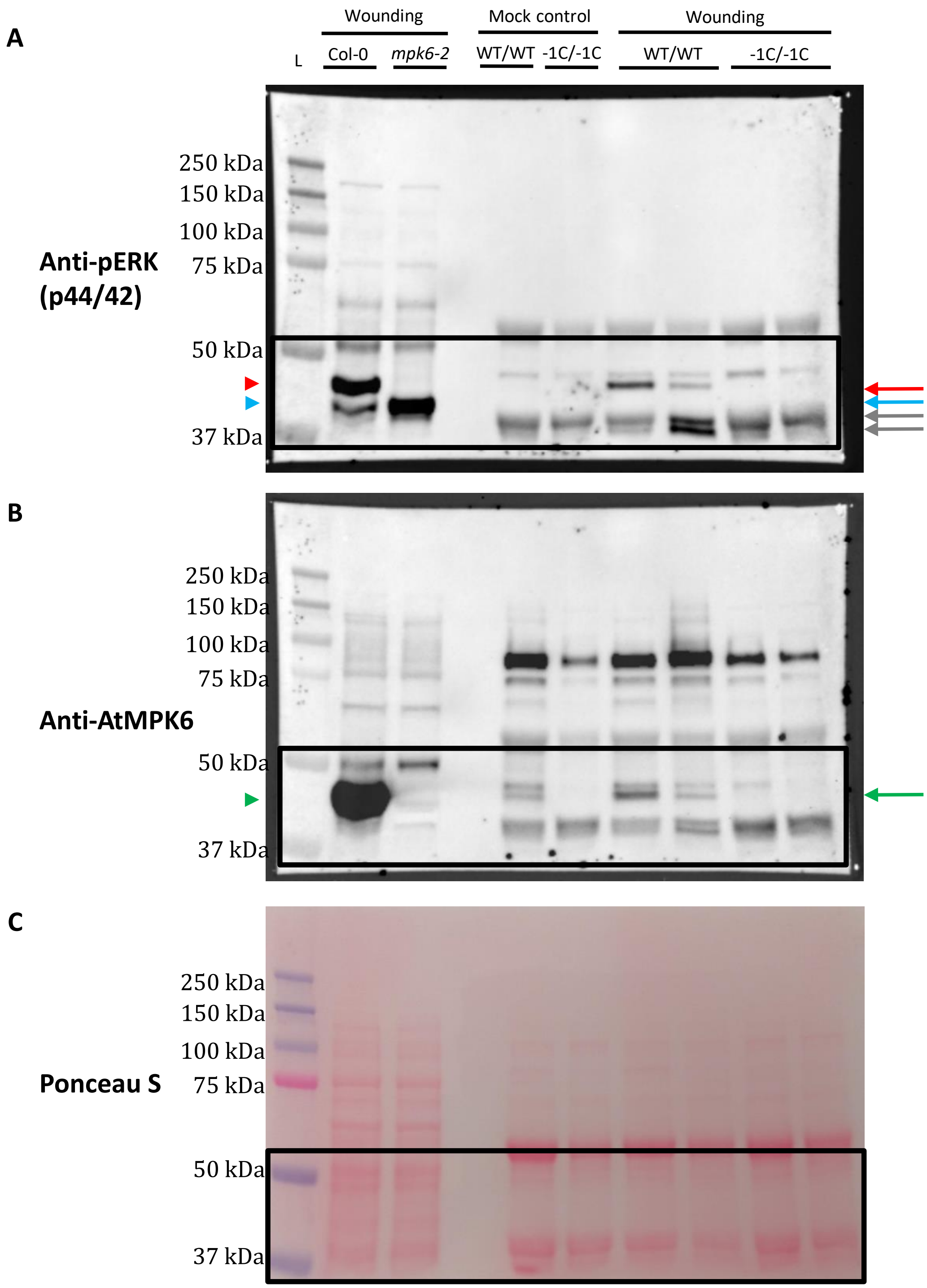

Supplementary Figure S11. Western blot analysis of the *Hvmpk6* mutant (-1C/-1C) and WT (WT/WT) endosperms with bran and husk – 1st experiment. The full scan of the whole immunoblotting membrane probed with **(A)** anti-pERK 1/2 and restriped with **(B)** anti-AtMPK6 antibody is presented. **(C)** For loading control, the whole immunoblotting membrane was stained with Ponceau S. The regions showed in open rectangles correspond to the membrane sections presented in Figure 10.

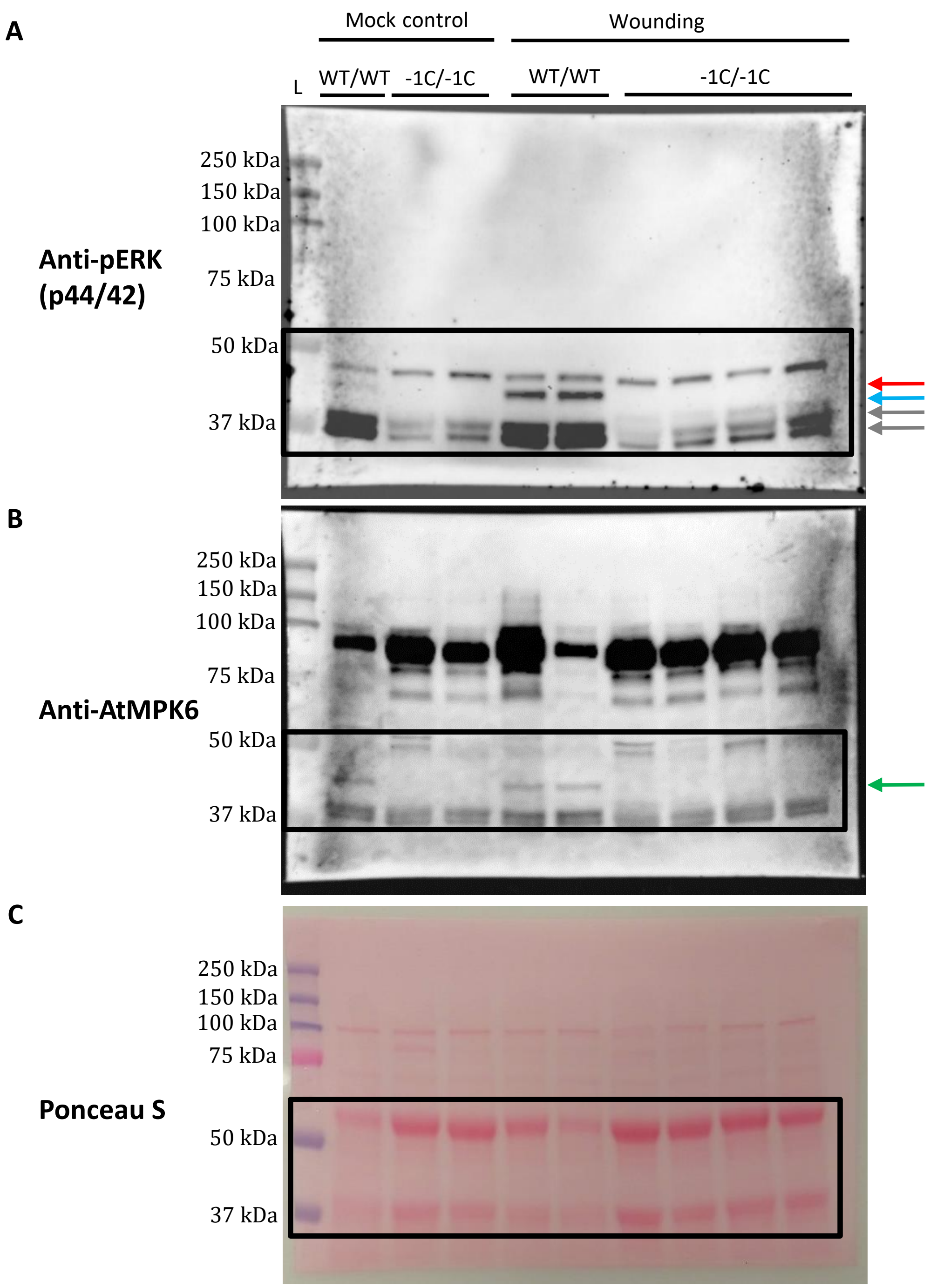

Supplementary Figure S12. Western blot analysis of the *Hvmpk6* mutant (-1C/-1C) and WT (WT/WT) endosperms with bran and husk – 2nd experiment. The full scan of the whole immunoblotting membrane probed with **(A)** anti-pERK 1/2 and restriped with **(B)** anti-AtMPK6 antibody is presented. **(C)** For loading control, the whole immunoblotting membrane was stained with Ponceau S. The regions showed in open rectangles correspond to the membrane sections presented in Figure 10.

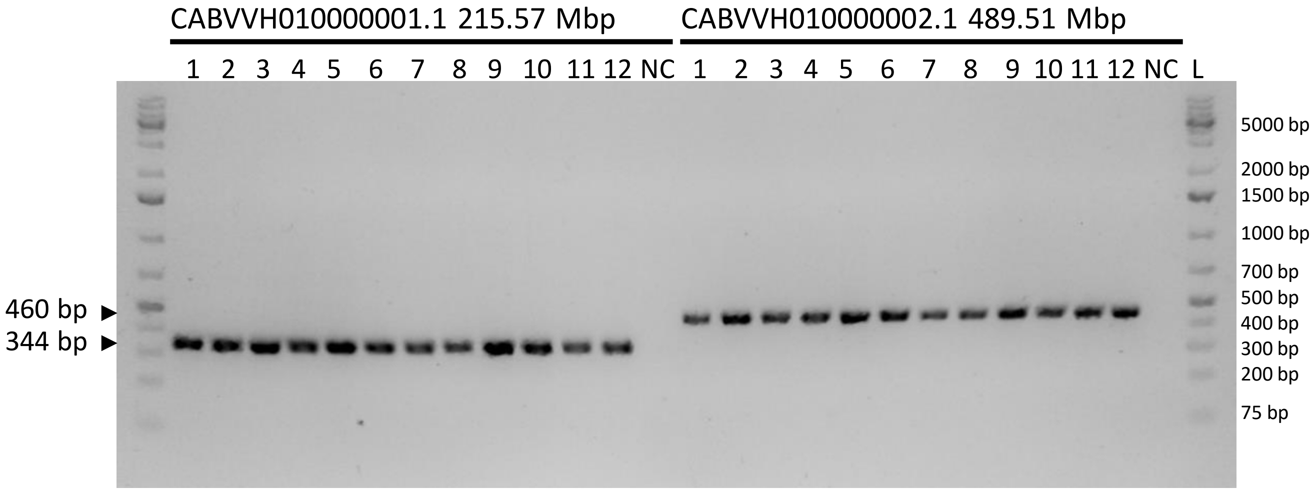

Supplementary Figure S13. PCR sequencing (PCR-Seq) genotyping of the T3 generation plants for the presence of the mutations in the predicted off-target sites. DNA fragments covering most probable off-target sites CABVVH010000001.1 215.57 Mbp and CABVVH010000002.1 489.51 Mbp (Supplementary Table S3) were amplified from genomic DNA of 12 emerged T3 generation A1 plants (numbered 1-12) using PCR with primer pairs Off215_F2/Off215_R2 and Off489_F2/Off489_R2, respectively (Supplementary Table S1). L - O'GeneRuler 1 kb Plus DNA Ladder (Thermo Fisher Scientific). The expected size of the PCR product is shown on the left and the sizes of the selected ladder fragments on the right. The single PCR amplicons of the expected size 344 bp and 460 bp were associated with all the analysed plants but not with negative water-template control (NC). All the PCR amplicons were assayed for the presence of the undesirable CC9-K6E3-induced mutations by Sanger sequencing (SEQme) with negative results (Supplementary Data Sheet 3 and 4).
