# Supplementary material for: CRISPR/Cas9-Induced Loss-of-Function Mutation in the Barley Mitogen-Activated Protein Kinase 6 Gene Causes Abnormal Embryo Development Leading to Severely Reduced Grain Germination and Seedling Shootless Phenotype
Source: Front Plant Sci. 2021 Jul 30;12:670302. doi: 10.3389/fpls.2021.670302 (PMC8361755; doi:10.3389/fpls.2021.670302)
Supplement: Data Sheet 1 — BLASTP: specificity of the “REALAFNPEYQQ” epitope toward HvMPK6. [file Data_Sheet_2.PDF]

```
BLASTP 2.9.0+


Reference: Stephen F. Altschul, Thomas L. Madden, Alejandro A.
Schaffer, Jinghui Zhang, Zheng Zhang, Webb Miller, and David J.
Lipman (1997), "Gapped BLAST and PSI-BLAST: a new generation of
protein database search programs", Nucleic Acids Res. 25:3389-3402.


Reference for composition-based statistics: Alejandro A. Schaffer,
L. Aravind, Thomas L. Madden, Sergei Shavirin, John L. Spouge, Yuri
I. Wolf, Eugene V. Koonin, and Stephen F. Altschul (2001),
"Improving the accuracy of PSI-BLAST protein database searches with
composition-based statistics and other refinements", Nucleic Acids
Res. 29:2994-3005.



Database: Hordeum_vulgare.IBSC_v2.pep.all
           236,301 sequences; 85,297,361 total letters



Query= EMBOSS_001

Length=12
                                                                Score
E
Sequences producing significant alignments:                    (Bits)
Value

EG:HORVU7Hr1G023760.4 pep chromosome:IBSC_v2:chr7H:37306939:37307...  25.8
8.8
EG:HORVU7Hr1G023760.2 pep chromosome:IBSC_v2:chr7H:37300013:37307...  25.8
8.8
EG:HORVU7Hr1G070970.9 pep chromosome:IBSC_v2:chr7H:386645188:3866...  24.9       17

EG:HORVU7Hr1G070970.3 pep chromosome:IBSC_v2:chr7H:386642830:3866...  24.9       17

EG:HORVU7Hr1G070970.1 pep chromosome:IBSC_v2:chr7H:386642801:3866...  24.9       17

EG:HORVU7Hr1G070970.6 pep chromosome:IBSC_v2:chr7H:386644102:3866...  24.9       17

EG:HORVU7Hr1G070970.2 pep chromosome:IBSC_v2:chr7H:386642827:3866...  24.9       17

EG:HORVU7Hr1G070970.4 pep chromosome:IBSC_v2:chr7H:386642961:3866...  24.9       17

EG:HORVU1Hr1G049500.1 pep chromosome:IBSC_v2:chr1H:366830180:3668...  23.0       59

EG:HORVU1Hr1G049500.3 pep chromosome:IBSC_v2:chr1H:366830181:3668...  23.0       59

EG:HORVU1Hr1G049500.2 pep chromosome:IBSC_v2:chr1H:366830180:3668...  23.0       59

EG:HORVU1Hr1G062160.6 pep chromosome:IBSC_v2:chr1H:448240858:4482...  22.6       81

EG:HORVU1Hr1G062160.1 pep chromosome:IBSC_v2:chr1H:448240781:4482...  22.6       81

EG:HORVU1Hr1G062160.5 pep chromosome:IBSC_v2:chr1H:448240858:4482...  22.6       81
```

EG:HORVU1Hr1G062160.2 pep chromosome:IBSC_v2:chr1H:448240855:4482... 22.6   81

EG:HORVU1Hr1G062160.11 pep chromosome:IBSC_v2:chr1H:448240919:448... 22.6   81

EG:HORVU1Hr1G062160.7 pep chromosome:IBSC_v2:chr1H:448240865:4482... 22.6   81

EG:HORVU5Hr1G103890.10 pep chromosome:IBSC_v2:chr5H:617535983:617... 22.1
111
EG:HORVU5Hr1G103890.7 pep chromosome:IBSC_v2:chr5H:617535946:6175... 22.1
111
EG:HORVU5Hr1G103890.17 pep chromosome:IBSC_v2:chr5H:617545668:617... 22.1
111
EG:HORVU5Hr1G103890.9 pep chromosome:IBSC_v2:chr5H:617535961:6175... 22.1
111
EG:HORVU5Hr1G103890.1 pep chromosome:IBSC_v2:chr5H:617535630:6175... 22.1
111
EG:HORVU5Hr1G103890.15 pep chromosome:IBSC_v2:chr5H:617536113:617... 22.1
111
EG:HORVU5Hr1G103890.12 pep chromosome:IBSC_v2:chr5H:617536005:617... 22.1
111
EG:HORVU5Hr1G103890.3 pep chromosome:IBSC_v2:chr5H:617535804:6176... 22.1
111
EG:HORVU5Hr1G103890.13 pep chromosome:IBSC_v2:chr5H:617536006:617... 22.1
111
EG:HORVU5Hr1G103890.6 pep chromosome:IBSC_v2:chr5H:617535942:6175... 22.1
111
EG:HORVU5Hr1G103890.8 pep chromosome:IBSC_v2:chr5H:617535961:6175... 22.1
111
EG:HORVU5Hr1G103890.18 pep chromosome:IBSC_v2:chr5H:617545750:617... 22.1
111
EG:HORVU5Hr1G103890.2 pep chromosome:IBSC_v2:chr5H:617535767:6176... 22.1
111
EG:HORVU5Hr1G103890.4 pep chromosome:IBSC_v2:chr5H:617535819:6175... 22.1
111
EG:HORVU5Hr1G103890.11 pep chromosome:IBSC_v2:chr5H:617535983:617... 22.1
111
EG:HORVU5Hr1G103890.14 pep chromosome:IBSC_v2:chr5H:617536055:617... 22.1
111
EG:HORVU3Hr1G005810.2 pep chromosome:IBSC_v2:chr3H:14179537:14182... 21.2
208
EG:HORVU3Hr1G005810.1 pep chromosome:IBSC_v2:chr3H:14179537:14182... 21.2
208
EG:HORVU6Hr1G081500.2 pep chromosome:IBSC_v2:chr6H:545914419:5459... 21.2
208
EG:HORVU6Hr1G081500.1 pep chromosome:IBSC_v2:chr6H:545914356:5459... 21.2
208
EG:HORVU6Hr1G030150.2 pep chromosome:IBSC_v2:chr6H:124984184:1249... 20.7
286
EG:HORVU6Hr1G030150.7 pep chromosome:IBSC_v2:chr6H:124984363:1249... 20.7
286
EG:HORVU6Hr1G030150.8 pep chromosome:IBSC_v2:chr6H:124984411:1249... 20.7
286
EG:HORVU3Hr1G094130.5 pep chromosome:IBSC_v2:chr3H:644974338:6449... 20.7
286
EG:HORVU3Hr1G094130.7 pep chromosome:IBSC_v2:chr3H:644974838:6449... 20.7
286
EG:HORVU3Hr1G094130.2 pep chromosome:IBSC_v2:chr3H:644974192:6449... 20.7
286
EG:HORVU3Hr1G094130.4 pep chromosome:IBSC_v2:chr3H:644974328:6449... 20.7

286
EG:HORVU3Hr1G094130.6 pep chromosome:IBSC_v2:chr3H:644974340:6449... 20.7
286
EG:HORVU3Hr1G094130.3 pep chromosome:IBSC_v2:chr3H:644974298:6449... 20.7
286
EG:HORVU3Hr1G094130.1 pep chromosome:IBSC_v2:chr3H:644974192:6449... 20.7
286
EG:HORVU5Hr1G079190.4 pep chromosome:IBSC_v2:chr5H:556484771:5564... 20.7
286
EG:HORVU3Hr1G005820.2 pep chromosome:IBSC_v2:chr3H:14192104:14193... 20.3
392
EG:HORVU3Hr1G005820.1 pep chromosome:IBSC_v2:chr3H:14192104:14193... 20.3
392
EG:HORVU3Hr1G005820.3 pep chromosome:IBSC_v2:chr3H:14192124:14193... 20.3
392
EG:HORVU3Hr1G113740.7 pep chromosome:IBSC_v2:chr3H:688984378:6889... 20.3
392
EG:HORVU3Hr1G113740.27 pep chromosome:IBSC_v2:chr3H:688989371:688... 20.3
392
EG:HORVU3Hr1G113740.23 pep chromosome:IBSC_v2:chr3H:688987797:688... 20.3
392
EG:HORVU3Hr1G113740.14 pep chromosome:IBSC_v2:chr3H:688984477:688... 20.3
392
EG:HORVU3Hr1G113740.9 pep chromosome:IBSC_v2:chr3H:688984432:6889... 20.3
392
EG:HORVU3Hr1G113740.2 pep chromosome:IBSC_v2:chr3H:688984300:6889... 20.3
392
EG:HORVU3Hr1G113740.28 pep chromosome:IBSC_v2:chr3H:688991150:688... 20.3
392
EG:HORVU3Hr1G113740.22 pep chromosome:IBSC_v2:chr3H:688987578:688... 20.3
392
EG:HORVU3Hr1G113740.18 pep chromosome:IBSC_v2:chr3H:688984499:688... 20.3
392
EG:HORVU3Hr1G113740.4 pep chromosome:IBSC_v2:chr3H:688984375:6889... 20.3
392
EG:HORVU3Hr1G113740.24 pep chromosome:IBSC_v2:chr3H:688987864:688... 20.3
392
EG:HORVU3Hr1G113740.1 pep chromosome:IBSC_v2:chr3H:688984300:6889... 20.3
392
EG:HORVU3Hr1G113740.3 pep chromosome:IBSC_v2:chr3H:688984329:6889... 20.3
392
EG:HORVU3Hr1G113740.8 pep chromosome:IBSC_v2:chr3H:688984419:6889... 20.3
392
EG:HORVU3Hr1G113740.13 pep chromosome:IBSC_v2:chr3H:688984457:688... 20.3
392
EG:HORVU3Hr1G113740.26 pep chromosome:IBSC_v2:chr3H:688988568:688... 20.3
392
EG:HORVU3Hr1G113740.15 pep chromosome:IBSC_v2:chr3H:688984479:688... 20.3
392
EG:HORVU3Hr1G113740.16 pep chromosome:IBSC_v2:chr3H:688984479:688... 20.3
392
EG:HORVU3Hr1G113740.5 pep chromosome:IBSC_v2:chr3H:688984375:6889... 20.3
392
EG:HORVU3Hr1G113740.25 pep chromosome:IBSC_v2:chr3H:688988343:688... 20.3
392
EG:HORVU3Hr1G113740.21 pep chromosome:IBSC_v2:chr3H:688986131:688... 20.3
392
EG:HORVU6Hr1G077520.1 pep chromosome:IBSC_v2:chr6H:530750660:5307... 20.3
392

EG:HORVU7Hr1G035550.3 pep chromosome:IBSC_v2:chr7H:79247352:79248... 20.3
392
EG:HORVU7Hr1G035550.1 pep chromosome:IBSC_v2:chr7H:79246546:79248... 20.3
392
EG:HORVU7Hr1G035550.2 pep chromosome:IBSC_v2:chr7H:79246549:79248... 20.3
392
EG:HORVU4Hr1G040280.4 pep chromosome:IBSC_v2:chr4H:309109269:3091... 19.8
538
EG:HORVU4Hr1G040280.3 pep chromosome:IBSC_v2:chr4H:309109196:3091... 19.8
538
EG:HORVU4Hr1G040280.2 pep chromosome:IBSC_v2:chr4H:309109182:3091... 19.8
538
EG:HORVU4Hr1G055900.2 pep chromosome:IBSC_v2:chr4H:469810729:4698... 19.8
538
EG:HORVU4Hr1G055900.6 pep chromosome:IBSC_v2:chr4H:469811093:4698... 19.8
538
EG:HORVU4Hr1G055900.8 pep chromosome:IBSC_v2:chr4H:469811093:4698... 19.8
538
EG:HORVU4Hr1G055900.1 pep chromosome:IBSC_v2:chr4H:469810551:4698... 19.8
538
EG:HORVU4Hr1G055900.7 pep chromosome:IBSC_v2:chr4H:469811093:4698... 19.8
538
EG:HORVU4Hr1G055900.5 pep chromosome:IBSC_v2:chr4H:469811093:4698... 19.8
538
EG:HORVU3Hr1G099420.2 pep chromosome:IBSC_v2:chr3H:661498454:6614... 19.8
538
EG:HORVU3Hr1G099420.1 pep chromosome:IBSC_v2:chr3H:661498454:6614... 19.8
538
EG:HORVU3Hr1G099420.3 pep chromosome:IBSC_v2:chr3H:661498454:6614... 19.8
538
EG:HORVU7Hr1G035590.4 pep chromosome:IBSC_v2:chr7H:79324864:79326... 19.8
538
EG:HORVU7Hr1G035590.2 pep chromosome:IBSC_v2:chr7H:79324751:79326... 19.8
538
EG:HORVU7Hr1G035590.3 pep chromosome:IBSC_v2:chr7H:79324845:79326... 19.8
538
EG:HORVU7Hr1G035590.1 pep chromosome:IBSC_v2:chr7H:79324382:79326... 19.8
538
EG:HORVU1Hr1G074650.14 pep chromosome:IBSC_v2:chr1H:508772354:508... 19.8
538
EG:HORVU1Hr1G074650.15 pep chromosome:IBSC_v2:chr1H:508772354:508... 19.8
538
EG:HORVU1Hr1G074650.21 pep chromosome:IBSC_v2:chr1H:508772366:508... 19.8
538
EG:HORVU1Hr1G074650.3 pep chromosome:IBSC_v2:chr1H:508771004:5087... 19.8
538
EG:HORVU1Hr1G074650.28 pep chromosome:IBSC_v2:chr1H:508772496:508... 19.8
538
EG:HORVU1Hr1G074650.13 pep chromosome:IBSC_v2:chr1H:508772348:508... 19.8
538
EG:HORVU1Hr1G074650.8 pep chromosome:IBSC_v2:chr1H:508772331:5087... 19.8
538
EG:HORVU1Hr1G074650.34 pep chromosome:IBSC_v2:chr1H:508773588:508... 19.8
538

>EG:HORVU7Hr1G023760.4 pep chromosome:IBSC_v2:chr7H:37306939:37307143:1
gene:HORVU7Hr1G023760 transcript:HORVU7Hr1G023760.4
gene_biotype:protein_coding transcript_biotype:protein_coding

```
description:Mitogen-activated protein kinase
[Source:UniProtKB/TrEMBL;Acc:A0A287VQ31]
Length=33

 Score = 25.8 bits (51),  Expect = 8.8
 Identities = 7/11 (64%), Positives = 10/11 (91%), Gaps = 0/11 (0%)

Query  1   REALAFNPEYQ  11
           +E +AFNP+YQ
Sbjct  23  QEGIAFNPDYQ  33


>EG:HORVU7Hr1G023760.2 pep chromosome:IBSC_v2:chr7H:37300013:37307500:1
gene:HORVU7Hr1G023760 transcript:HORVU7Hr1G023760.2
gene_biotype:protein_coding transcript_biotype:protein_coding
description:Mitogen-activated protein kinase
[Source:UniProtKB/TrEMBL;Acc:A0A287VQ31]
Length=367

 Score = 25.8 bits (51),  Expect = 8.8
 Identities = 7/11 (64%), Positives = 10/11 (91%), Gaps = 0/11 (0%)

Query  1    REALAFNPEYQ  11
            +E +AFNP+YQ
Sbjct  357  QEGIAFNPDYQ  367


>EG:HORVU7Hr1G070970.9 pep chromosome:IBSC_v2:chr7H:386645188:386646158:1
gene:HORVU7Hr1G070970 transcript:HORVU7Hr1G070970.9
gene_biotype:protein_coding transcript_biotype:protein_coding
Length=124

 Score = 24.9 bits (49),  Expect = 17
 Identities = 8/9 (89%), Positives = 8/9 (89%), Gaps = 0/9 (0%)

Query  1    REALAFNPE  9
            REAL FNPE
Sbjct  113  REALKFNPE  121


>EG:HORVU7Hr1G070970.3 pep chromosome:IBSC_v2:chr7H:386642830:386646131:1
gene:HORVU7Hr1G070970 transcript:HORVU7Hr1G070970.3
gene_biotype:protein_coding transcript_biotype:protein_coding
Length=280

 Score = 24.9 bits (49),  Expect = 17
 Identities = 8/9 (89%), Positives = 8/9 (89%), Gaps = 0/9 (0%)

Query  1    REALAFNPE  9
            REAL FNPE
Sbjct  269  REALKFNPE  277


>EG:HORVU7Hr1G070970.1 pep chromosome:IBSC_v2:chr7H:386642801:386646151:1
gene:HORVU7Hr1G070970 transcript:HORVU7Hr1G070970.1
gene_biotype:protein_coding transcript_biotype:protein_coding
Length=390

 Score = 24.9 bits (49),  Expect = 17
```

```
 Identities = 8/9 (89%), Positives = 8/9 (89%), Gaps = 0/9 (0%)

Query  1    REALAFNPE  9
            REAL FNPE
Sbjct  379  REALKFNPE  387


>EG:HORVU7Hr1G070970.6 pep chromosome:IBSC_v2:chr7H:386644102:386646055:1
gene:HORVU7Hr1G070970 transcript:HORVU7Hr1G070970.6
gene_biotype:protein_coding transcript_biotype:protein_coding
Length=250

 Score = 24.9 bits (49),  Expect = 17
 Identities = 8/9 (89%), Positives = 8/9 (89%), Gaps = 0/9 (0%)

Query  1    REALAFNPE  9
            REAL FNPE
Sbjct  239  REALKFNPE  247


>EG:HORVU7Hr1G070970.2 pep chromosome:IBSC_v2:chr7H:386642827:386646046:1
gene:HORVU7Hr1G070970 transcript:HORVU7Hr1G070970.2
gene_biotype:protein_coding transcript_biotype:protein_coding
Length=422

 Score = 24.9 bits (49),  Expect = 17
 Identities = 8/9 (89%), Positives = 8/9 (89%), Gaps = 0/9 (0%)

Query  1    REALAFNPE  9
            REAL FNPE
Sbjct  411  REALKFNPE  419


>EG:HORVU7Hr1G070970.4 pep chromosome:IBSC_v2:chr7H:386642961:386646110:1
gene:HORVU7Hr1G070970 transcript:HORVU7Hr1G070970.4
gene_biotype:protein_coding transcript_biotype:protein_coding
Length=250

 Score = 24.9 bits (49),  Expect = 17
 Identities = 8/9 (89%), Positives = 8/9 (89%), Gaps = 0/9 (0%)

Query  1    REALAFNPE  9
            REAL FNPE
Sbjct  239  REALKFNPE  247


>EG:HORVU1Hr1G049500.1 pep chromosome:IBSC_v2:chr1H:366830180:366834352:-1
gene:HORVU1Hr1G049500 transcript:HORVU1Hr1G049500.1
gene_biotype:protein_coding transcript_biotype:protein_coding
description:Mitogen-activated protein kinase [Source:UniProtKB/TrEMBL;Acc:F2DS97]
Length=377

 Score = 23.0 bits (45),  Expect = 59
 Identities = 7/9 (78%), Positives = 8/9 (89%), Gaps = 0/9 (0%)

Query  1    REALAFNPE  9
            RE LAFNP+
Sbjct  366  RETLAFNPD  374
```

```
>EG:HORVU1Hr1G049500.3 pep chromosome:IBSC_v2:chr1H:366830181:366833841:-1
gene:HORVU1Hr1G049500 transcript:HORVU1Hr1G049500.3
gene_biotype:protein_coding transcript_biotype:protein_coding
description:Mitogen-activated protein kinase [Source:UniProtKB/TrEMBL;Acc:F2DS97]
Length=370

 Score = 23.0 bits (45),  Expect = 59
 Identities = 7/9 (78%), Positives = 8/9 (89%), Gaps = 0/9 (0%)

Query  1    REALAFNPE  9
            RE LAFNP+
Sbjct  359  RETLAFNPD  367


>EG:HORVU1Hr1G049500.2 pep chromosome:IBSC_v2:chr1H:366830180:366834352:-1
gene:HORVU1Hr1G049500 transcript:HORVU1Hr1G049500.2
gene_biotype:protein_coding transcript_biotype:protein_coding
description:Mitogen-activated protein kinase [Source:UniProtKB/TrEMBL;Acc:F2DS97]
Length=377

 Score = 23.0 bits (45),  Expect = 59
 Identities = 7/9 (78%), Positives = 8/9 (89%), Gaps = 0/9 (0%)

Query  1    REALAFNPE  9
            RE LAFNP+
Sbjct  366  RETLAFNPD  374


>EG:HORVU1Hr1G062160.6 pep chromosome:IBSC_v2:chr1H:448240858:448257233:-1
gene:HORVU1Hr1G062160 transcript:HORVU1Hr1G062160.6
gene_biotype:protein_coding transcript_biotype:protein_coding
Length=539

 Score = 22.6 bits (44),  Expect = 81
 Identities = 7/9 (78%), Positives = 7/9 (78%), Gaps = 0/9 (0%)

Query  4    LAFNPEYQQ  12
            LA  PEYQQ
Sbjct  186  LAVKPEYQQ  194


>EG:HORVU1Hr1G062160.1 pep chromosome:IBSC_v2:chr1H:448240781:448257308:-1
gene:HORVU1Hr1G062160 transcript:HORVU1Hr1G062160.1
gene_biotype:protein_coding transcript_biotype:protein_coding
Length=720

 Score = 22.6 bits (44),  Expect = 81
 Identities = 7/9 (78%), Positives = 7/9 (78%), Gaps = 0/9 (0%)

Query  4    LAFNPEYQQ  12
            LA  PEYQQ
Sbjct  147  LAVKPEYQQ  155


>EG:HORVU1Hr1G062160.5 pep chromosome:IBSC_v2:chr1H:448240858:448257233:-1
gene:HORVU1Hr1G062160 transcript:HORVU1Hr1G062160.5
gene_biotype:protein_coding transcript_biotype:protein_coding
Length=476
```

```
 Score = 22.6 bits (44),  Expect = 81
 Identities = 7/9 (78%), Positives = 7/9 (78%), Gaps = 0/9 (0%)

Query  4    LAFNPEYQQ  12
            LA  PEYQQ
Sbjct  186  LAVKPEYQQ  194


>EG:HORVU1Hr1G062160.2 pep chromosome:IBSC_v2:chr1H:448240855:448257158:-1
gene:HORVU1Hr1G062160 transcript:HORVU1Hr1G062160.2
gene_biotype:protein_coding transcript_biotype:protein_coding
Length=613

 Score = 22.6 bits (44),  Expect = 81
 Identities = 7/9 (78%), Positives = 7/9 (78%), Gaps = 0/9 (0%)

Query  4   LAFNPEYQQ  12
           LA  PEYQQ
Sbjct  40  LAVKPEYQQ  48


>EG:HORVU1Hr1G062160.11 pep chromosome:IBSC_v2:chr1H:448240919:448257233:-1
gene:HORVU1Hr1G062160 transcript:HORVU1Hr1G062160.11
gene_biotype:protein_coding transcript_biotype:protein_coding
Length=276

 Score = 22.6 bits (44),  Expect = 81
 Identities = 7/9 (78%), Positives = 7/9 (78%), Gaps = 0/9 (0%)

Query  4    LAFNPEYQQ  12
            LA  PEYQQ
Sbjct  186  LAVKPEYQQ  194


>EG:HORVU1Hr1G062160.7 pep chromosome:IBSC_v2:chr1H:448240865:448266713:-1
gene:HORVU1Hr1G062160 transcript:HORVU1Hr1G062160.7
gene_biotype:protein_coding transcript_biotype:protein_coding
Length=709

 Score = 22.6 bits (44),  Expect = 81
 Identities = 7/9 (78%), Positives = 7/9 (78%), Gaps = 0/9 (0%)

Query  4    LAFNPEYQQ  12
            LA  PEYQQ
Sbjct  136  LAVKPEYQQ  144


>EG:HORVU5Hr1G103890.10 pep chromosome:IBSC_v2:chr5H:617535983:617554060:1
gene:HORVU5Hr1G103890 transcript:HORVU5Hr1G103890.10
gene_biotype:protein_coding transcript_biotype:protein_coding
Length=714

 Score = 22.1 bits (43),  Expect = 111
 Identities = 7/11 (64%), Positives = 8/11 (73%), Gaps = 0/11 (0%)

Query  1    REALAFNPEYQ  11
            R+ L FN EYQ
Sbjct  529  RQELVFNDEYQ  539
```

```
>EG:HORVU5Hr1G103890.7 pep chromosome:IBSC_v2:chr5H:617535946:617550855:1
gene:HORVU5Hr1G103890 transcript:HORVU5Hr1G103890.7
gene_biotype:protein_coding transcript_biotype:protein_coding
Length=1147

 Score = 22.1 bits (43),  Expect = 111
 Identities = 7/11 (64%), Positives = 8/11 (73%), Gaps = 0/11 (0%)

Query  1    REALAFNPEYQ  11
            R+ L FN EYQ
Sbjct  541  RQELVFNDEYQ  551


>EG:HORVU5Hr1G103890.17 pep chromosome:IBSC_v2:chr5H:617545668:617550744:1
gene:HORVU5Hr1G103890 transcript:HORVU5Hr1G103890.17
gene_biotype:protein_coding transcript_biotype:protein_coding
Length=1122

 Score = 22.1 bits (43),  Expect = 111
 Identities = 7/11 (64%), Positives = 8/11 (73%), Gaps = 0/11 (0%)

Query  1    REALAFNPEYQ  11
            R+ L FN EYQ
Sbjct  516  RQELVFNDEYQ  526


>EG:HORVU5Hr1G103890.9 pep chromosome:IBSC_v2:chr5H:617535961:617550855:1
gene:HORVU5Hr1G103890 transcript:HORVU5Hr1G103890.9
gene_biotype:protein_coding transcript_biotype:protein_coding
Length=1136

 Score = 22.1 bits (43),  Expect = 111
 Identities = 7/11 (64%), Positives = 8/11 (73%), Gaps = 0/11 (0%)

Query  1    REALAFNPEYQ  11
            R+ L FN EYQ
Sbjct  530  RQELVFNDEYQ  540


>EG:HORVU5Hr1G103890.1 pep chromosome:IBSC_v2:chr5H:617535630:617554068:1
gene:HORVU5Hr1G103890 transcript:HORVU5Hr1G103890.1
gene_biotype:protein_coding transcript_biotype:protein_coding
Length=1019

 Score = 22.1 bits (43),  Expect = 111
 Identities = 7/11 (64%), Positives = 8/11 (73%), Gaps = 0/11 (0%)

Query  1    REALAFNPEYQ  11
            R+ L FN EYQ
Sbjct  568  RQELVFNDEYQ  578


>EG:HORVU5Hr1G103890.15 pep chromosome:IBSC_v2:chr5H:617536113:617554064:1
gene:HORVU5Hr1G103890 transcript:HORVU5Hr1G103890.15
gene_biotype:protein_coding transcript_biotype:protein_coding
Length=735
```

```
 Score = 22.1 bits (43),  Expect = 111
 Identities = 7/11 (64%), Positives = 8/11 (73%), Gaps = 0/11 (0%)

Query  1    REALAFNPEYQ  11
            R+ L FN EYQ
Sbjct  530  RQELVFNDEYQ  540


>EG:HORVU5Hr1G103890.12 pep chromosome:IBSC_v2:chr5H:617536005:617554064:1
gene:HORVU5Hr1G103890 transcript:HORVU5Hr1G103890.12
gene_biotype:protein_coding transcript_biotype:protein_coding
Length=979

 Score = 22.1 bits (43),  Expect = 111
 Identities = 7/11 (64%), Positives = 8/11 (73%), Gaps = 0/11 (0%)

Query  1    REALAFNPEYQ  11
            R+ L FN EYQ
Sbjct  530  RQELVFNDEYQ  540


>EG:HORVU5Hr1G103890.3 pep chromosome:IBSC_v2:chr5H:617535804:617617167:1
gene:HORVU5Hr1G103890 transcript:HORVU5Hr1G103890.3
gene_biotype:protein_coding transcript_biotype:protein_coding
Length=1212

 Score = 22.1 bits (43),  Expect = 111
 Identities = 7/11 (64%), Positives = 8/11 (73%), Gaps = 0/11 (0%)

Query  1    REALAFNPEYQ  11
            R+ L FN EYQ
Sbjct  568  RQELVFNDEYQ  578


>EG:HORVU5Hr1G103890.13 pep chromosome:IBSC_v2:chr5H:617536006:617548392:1
gene:HORVU5Hr1G103890 transcript:HORVU5Hr1G103890.13
gene_biotype:protein_coding transcript_biotype:protein_coding
Length=659

 Score = 22.1 bits (43),  Expect = 111
 Identities = 7/11 (64%), Positives = 8/11 (73%), Gaps = 0/11 (0%)

Query  1    REALAFNPEYQ  11
            R+ L FN EYQ
Sbjct  521  RQELVFNDEYQ  531


>EG:HORVU5Hr1G103890.6 pep chromosome:IBSC_v2:chr5H:617535942:617549063:1
gene:HORVU5Hr1G103890 transcript:HORVU5Hr1G103890.6
gene_biotype:protein_coding transcript_biotype:protein_coding
Length=720

 Score = 22.1 bits (43),  Expect = 111
 Identities = 7/11 (64%), Positives = 8/11 (73%), Gaps = 0/11 (0%)

Query  1    REALAFNPEYQ  11
            R+ L FN EYQ
Sbjct  440  RQELVFNDEYQ  450
```

```
>EG:HORVU5Hr1G103890.8 pep chromosome:IBSC_v2:chr5H:617535961:617548303:1
gene:HORVU5Hr1G103890 transcript:HORVU5Hr1G103890.8
gene_biotype:protein_coding transcript_biotype:protein_coding
Length=655

 Score = 22.1 bits (43),  Expect = 111
 Identities = 7/11 (64%), Positives = 8/11 (73%), Gaps = 0/11 (0%)

Query  1    REALAFNPEYQ  11
            R+ L FN EYQ
Sbjct  530  RQELVFNDEYQ  540


>EG:HORVU5Hr1G103890.18 pep chromosome:IBSC_v2:chr5H:617545750:617547578:1
gene:HORVU5Hr1G103890 transcript:HORVU5Hr1G103890.18
gene_biotype:protein_coding transcript_biotype:protein_coding
Length=609

 Score = 22.1 bits (43),  Expect = 111
 Identities = 7/11 (64%), Positives = 8/11 (73%), Gaps = 0/11 (0%)

Query  1    REALAFNPEYQ  11
            R+ L FN EYQ
Sbjct  586  RQELVFNDEYQ  596


>EG:HORVU5Hr1G103890.2 pep chromosome:IBSC_v2:chr5H:617535767:617617167:1
gene:HORVU5Hr1G103890 transcript:HORVU5Hr1G103890.2
gene_biotype:protein_coding transcript_biotype:protein_coding
Length=1174

 Score = 22.1 bits (43),  Expect = 111
 Identities = 7/11 (64%), Positives = 8/11 (73%), Gaps = 0/11 (0%)

Query  1    REALAFNPEYQ  11
            R+ L FN EYQ
Sbjct  530  RQELVFNDEYQ  540


>EG:HORVU5Hr1G103890.4 pep chromosome:IBSC_v2:chr5H:617535819:617549092:1
gene:HORVU5Hr1G103890 transcript:HORVU5Hr1G103890.4
gene_biotype:protein_coding transcript_biotype:protein_coding
Length=789

 Score = 22.1 bits (43),  Expect = 111
 Identities = 7/11 (64%), Positives = 8/11 (73%), Gaps = 0/11 (0%)

Query  1    REALAFNPEYQ  11
            R+ L FN EYQ
Sbjct  584  RQELVFNDEYQ  594


>EG:HORVU5Hr1G103890.11 pep chromosome:IBSC_v2:chr5H:617535983:617554060:1
gene:HORVU5Hr1G103890 transcript:HORVU5Hr1G103890.11
gene_biotype:protein_coding transcript_biotype:protein_coding
Length=716

 Score = 22.1 bits (43),  Expect = 111
```

```
 Identities = 7/11 (64%), Positives = 8/11 (73%), Gaps = 0/11 (0%)

Query  1    REALAFNPEYQ  11
            R+ L FN EYQ
Sbjct  529  RQELVFNDEYQ  539


>EG:HORVU5Hr1G103890.14 pep chromosome:IBSC_v2:chr5H:617536055:617554064:1
gene:HORVU5Hr1G103890 transcript:HORVU5Hr1G103890.14
gene_biotype:protein_coding transcript_biotype:protein_coding
Length=979

 Score = 22.1 bits (43),  Expect = 111
 Identities = 7/11 (64%), Positives = 8/11 (73%), Gaps = 0/11 (0%)

Query  1    REALAFNPEYQ  11
            R+ L FN EYQ
Sbjct  530  RQELVFNDEYQ  540


>EG:HORVU3Hr1G005810.2 pep chromosome:IBSC_v2:chr3H:14179537:14182200:-1
gene:HORVU3Hr1G005810 transcript:HORVU3Hr1G005810.2
gene_biotype:protein_coding transcript_biotype:protein_coding
Length=163

 Score = 21.2 bits (41),  Expect = 208
 Identities = 6/10 (60%), Positives = 8/10 (80%), Gaps = 0/10 (0%)

Query  1   REALAFNPEY  10
           R++L  NPEY
Sbjct  20  RQSLEINPEY  29


>EG:HORVU3Hr1G005810.1 pep chromosome:IBSC_v2:chr3H:14179537:14182200:-1
gene:HORVU3Hr1G005810 transcript:HORVU3Hr1G005810.1
gene_biotype:protein_coding transcript_biotype:protein_coding
Length=208

 Score = 21.2 bits (41),  Expect = 208
 Identities = 6/10 (60%), Positives = 8/10 (80%), Gaps = 0/10 (0%)

Query  1   REALAFNPEY  10
           R++L  NPEY
Sbjct  65  RQSLEINPEY  74


>EG:HORVU6Hr1G081500.2 pep chromosome:IBSC_v2:chr6H:545914419:545916385:1
gene:HORVU6Hr1G081500 transcript:HORVU6Hr1G081500.2
gene_biotype:protein_coding transcript_biotype:protein_coding
description:Endoglucanase [Source:UniProtKB/TrEMBL;Acc:A0A287UX16]
Length=502

 Score = 21.2 bits (41),  Expect = 208
 Identities = 7/9 (78%), Positives = 7/9 (78%), Gaps = 0/9 (0%)

Query  3    ALAFNPEYQ  11
            ALAF PE Q
Sbjct  115  ALAFKPELQ  123
```

```
>EG:HORVU6Hr1G081500.1 pep chromosome:IBSC_v2:chr6H:545914356:545916683:1
gene:HORVU6Hr1G081500 transcript:HORVU6Hr1G081500.1
gene_biotype:protein_coding transcript_biotype:protein_coding
description:Endoglucanase [Source:UniProtKB/TrEMBL;Acc:A0A287UX16]
Length=521

 Score = 21.2 bits (41),  Expect = 208
 Identities = 7/9 (78%), Positives = 7/9 (78%), Gaps = 0/9 (0%)

Query  3    ALAFNPEYQ  11
            ALAF PE Q
Sbjct  134  ALAFKPELQ  142


>EG:HORVU6Hr1G030150.2 pep chromosome:IBSC_v2:chr6H:124984184:124992048:1
gene:HORVU6Hr1G030150 transcript:HORVU6Hr1G030150.2
gene_biotype:protein_coding transcript_biotype:protein_coding
description:Non-specific serine/threonine protein kinase
[Source:UniProtKB/TrEMBL;Acc:A0A287TRZ3]
Length=442

 Score = 20.7 bits (40),  Expect = 286
 Identities = 8/12 (67%), Positives = 9/12 (75%), Gaps = 0/12 (0%)

Query  1    REALAFNPEYQQ  12
            REA A NPE +Q
Sbjct  300  REAGAANPEPEQ  311


>EG:HORVU6Hr1G030150.7 pep chromosome:IBSC_v2:chr6H:124984363:124987191:1
gene:HORVU6Hr1G030150 transcript:HORVU6Hr1G030150.7
gene_biotype:protein_coding transcript_biotype:protein_coding
description:Non-specific serine/threonine protein kinase
[Source:UniProtKB/TrEMBL;Acc:A0A287TRZ3]
Length=348

 Score = 20.7 bits (40),  Expect = 286
 Identities = 8/12 (67%), Positives = 9/12 (75%), Gaps = 0/12 (0%)

Query  1    REALAFNPEYQQ  12
            REA A NPE +Q
Sbjct  211  REAGAANPEPEQ  222


>EG:HORVU6Hr1G030150.8 pep chromosome:IBSC_v2:chr6H:124984411:124987209:1
gene:HORVU6Hr1G030150 transcript:HORVU6Hr1G030150.8
gene_biotype:protein_coding transcript_biotype:protein_coding
description:Non-specific serine/threonine protein kinase
[Source:UniProtKB/TrEMBL;Acc:A0A287TRZ3]
Length=354

 Score = 20.7 bits (40),  Expect = 286
 Identities = 8/12 (67%), Positives = 9/12 (75%), Gaps = 0/12 (0%)

Query  1    REALAFNPEYQQ  12
            REA A NPE +Q
Sbjct  212  REAGAANPEPEQ  223
```

```
>EG:HORVU3Hr1G094130.5 pep chromosome:IBSC_v2:chr3H:644974338:644977283:-1
gene:HORVU3Hr1G094130 transcript:HORVU3Hr1G094130.5
gene_biotype:protein_coding transcript_biotype:protein_coding
Length=348

 Score = 20.7 bits (40),  Expect = 286
 Identities = 6/8 (75%), Positives = 8/8 (100%), Gaps = 0/8 (0%)

Query  1   REALAFNP  8
           R+ALA+NP
Sbjct  77  RDALAYNP  84


>EG:HORVU3Hr1G094130.7 pep chromosome:IBSC_v2:chr3H:644974838:644977140:-1
gene:HORVU3Hr1G094130 transcript:HORVU3Hr1G094130.7
gene_biotype:protein_coding transcript_biotype:protein_coding
Length=300

 Score = 20.7 bits (40),  Expect = 286
 Identities = 6/8 (75%), Positives = 8/8 (100%), Gaps = 0/8 (0%)

Query  1   REALAFNP  8
           R+ALA+NP
Sbjct  29  RDALAYNP  36


>EG:HORVU3Hr1G094130.2 pep chromosome:IBSC_v2:chr3H:644974192:644977377:-1
gene:HORVU3Hr1G094130 transcript:HORVU3Hr1G094130.2
gene_biotype:protein_coding transcript_biotype:protein_coding
Length=379

 Score = 20.7 bits (40),  Expect = 286
 Identities = 6/8 (75%), Positives = 8/8 (100%), Gaps = 0/8 (0%)

Query  1    REALAFNP  8
            R+ALA+NP
Sbjct  108  RDALAYNP  115


>EG:HORVU3Hr1G094130.4 pep chromosome:IBSC_v2:chr3H:644974328:644977588:-1
gene:HORVU3Hr1G094130 transcript:HORVU3Hr1G094130.4
gene_biotype:protein_coding transcript_biotype:protein_coding
Length=450

 Score = 20.7 bits (40),  Expect = 286
 Identities = 6/8 (75%), Positives = 8/8 (100%), Gaps = 0/8 (0%)

Query  1    REALAFNP  8
            R+ALA+NP
Sbjct  179  RDALAYNP  186


>EG:HORVU3Hr1G094130.6 pep chromosome:IBSC_v2:chr3H:644974340:644977537:-1
gene:HORVU3Hr1G094130 transcript:HORVU3Hr1G094130.6
gene_biotype:protein_coding transcript_biotype:protein_coding
Length=433

 Score = 20.7 bits (40),  Expect = 286
```

```
 Identities = 6/8 (75%), Positives = 8/8 (100%), Gaps = 0/8 (0%)

Query  1    REALAFNP  8
            R+ALA+NP
Sbjct  162  RDALAYNP  169


>EG:HORVU3Hr1G094130.3 pep chromosome:IBSC_v2:chr3H:644974298:644977140:-1
gene:HORVU3Hr1G094130 transcript:HORVU3Hr1G094130.3
gene_biotype:protein_coding transcript_biotype:protein_coding
Length=300

 Score = 20.7 bits (40),  Expect = 286
 Identities = 6/8 (75%), Positives = 8/8 (100%), Gaps = 0/8 (0%)

Query  1   REALAFNP  8
           R+ALA+NP
Sbjct  29  RDALAYNP  36


>EG:HORVU3Hr1G094130.1 pep chromosome:IBSC_v2:chr3H:644974192:644977377:-1
gene:HORVU3Hr1G094130 transcript:HORVU3Hr1G094130.1
gene_biotype:protein_coding transcript_biotype:protein_coding
Length=379

 Score = 20.7 bits (40),  Expect = 286
 Identities = 6/8 (75%), Positives = 8/8 (100%), Gaps = 0/8 (0%)

Query  1    REALAFNP  8
            R+ALA+NP
Sbjct  108  RDALAYNP  115


>EG:HORVU5Hr1G079190.4 pep chromosome:IBSC_v2:chr5H:556484771:556490997:1
gene:HORVU5Hr1G079190 transcript:HORVU5Hr1G079190.4
gene_biotype:protein_coding transcript_biotype:protein_coding
description:Flowering time control protein FPA [Source:Projected
from Arabidopsis thaliana AT2G43410 UniProtKB/Swiss-Prot;Acc:Q8LPQ9]
Length=755

 Score = 20.7 bits (40),  Expect = 286
 Identities = 6/8 (75%), Positives = 7/8 (88%), Gaps = 0/8 (0%)

Query  5    AFNPEYQQ  12
            A+NPE QQ
Sbjct  705  AYNPEVQQ  712


>EG:HORVU3Hr1G005820.2 pep chromosome:IBSC_v2:chr3H:14192104:14193862:1
gene:HORVU3Hr1G005820 transcript:HORVU3Hr1G005820.2
gene_biotype:protein_coding transcript_biotype:protein_coding
Length=505

 Score = 20.3 bits (39),  Expect = 392
 Identities = 6/9 (67%), Positives = 8/9 (89%), Gaps = 0/9 (0%)

Query  2    EALAFNPEY  10
            ++LA NPEY
Sbjct  349  QSLATNPEY  357
```

```
>EG:HORVU3Hr1G005820.1 pep chromosome:IBSC_v2:chr3H:14192104:14193651:1
gene:HORVU3Hr1G005820 transcript:HORVU3Hr1G005820.1
gene_biotype:protein_coding transcript_biotype:protein_coding
Length=506

 Score = 20.3 bits (39),  Expect = 392
 Identities = 6/9 (67%), Positives = 8/9 (89%), Gaps = 0/9 (0%)

Query  2    EALAFNPEY  10
            ++LA NPEY
Sbjct  349  QSLATNPEY  357


>EG:HORVU3Hr1G005820.3 pep chromosome:IBSC_v2:chr3H:14192124:14193641:1
gene:HORVU3Hr1G005820 transcript:HORVU3Hr1G005820.3
gene_biotype:protein_coding transcript_biotype:protein_coding
Length=256

 Score = 20.3 bits (39),  Expect = 392
 Identities = 6/9 (67%), Positives = 8/9 (89%), Gaps = 0/9 (0%)

Query  2    EALAFNPEY  10
            ++LA NPEY
Sbjct  99   QSLATNPEY  107


>EG:HORVU3Hr1G113740.7 pep chromosome:IBSC_v2:chr3H:688984378:688997947:1
gene:HORVU3Hr1G113740 transcript:HORVU3Hr1G113740.7
gene_biotype:protein_coding transcript_biotype:protein_coding
Length=285

 Score = 20.3 bits (39),  Expect = 392
 Identities = 6/8 (75%), Positives = 7/8 (88%), Gaps = 0/8 (0%)

Query  2    EALAFNPE  9
            EAL FNP+
Sbjct  136  EALQFNPQ  143


>EG:HORVU3Hr1G113740.27 pep chromosome:IBSC_v2:chr3H:688989371:688999459:1
gene:HORVU3Hr1G113740 transcript:HORVU3Hr1G113740.27
gene_biotype:protein_coding transcript_biotype:protein_coding
Length=449

 Score = 20.3 bits (39),  Expect = 392
 Identities = 6/8 (75%), Positives = 7/8 (88%), Gaps = 0/8 (0%)

Query  2    EALAFNPE  9
            EAL FNP+
Sbjct  285  EALQFNPQ  292


>EG:HORVU3Hr1G113740.23 pep chromosome:IBSC_v2:chr3H:688987797:688999415:1
gene:HORVU3Hr1G113740 transcript:HORVU3Hr1G113740.23
gene_biotype:protein_coding transcript_biotype:protein_coding
Length=280
```

```
 Score = 20.3 bits (39),  Expect = 392
 Identities = 6/8 (75%), Positives = 7/8 (88%), Gaps = 0/8 (0%)

Query  2    EALAFNPE  9
            EAL FNP+
Sbjct  136  EALQFNPQ  143


>EG:HORVU3Hr1G113740.14 pep chromosome:IBSC_v2:chr3H:688984477:688999441:1
gene:HORVU3Hr1G113740 transcript:HORVU3Hr1G113740.14
gene_biotype:protein_coding transcript_biotype:protein_coding
Length=753

 Score = 20.3 bits (39),  Expect = 392
 Identities = 6/8 (75%), Positives = 7/8 (88%), Gaps = 0/8 (0%)

Query  2    EALAFNPE  9
            EAL FNP+
Sbjct  589  EALQFNPQ  596


>EG:HORVU3Hr1G113740.9 pep chromosome:IBSC_v2:chr3H:688984432:688998029:1
gene:HORVU3Hr1G113740 transcript:HORVU3Hr1G113740.9
gene_biotype:protein_coding transcript_biotype:protein_coding
Length=280

 Score = 20.3 bits (39),  Expect = 392
 Identities = 6/8 (75%), Positives = 7/8 (88%), Gaps = 0/8 (0%)

Query  2    EALAFNPE  9
            EAL FNP+
Sbjct  136  EALQFNPQ  143


>EG:HORVU3Hr1G113740.2 pep chromosome:IBSC_v2:chr3H:688984300:688999413:1
gene:HORVU3Hr1G113740 transcript:HORVU3Hr1G113740.2
gene_biotype:protein_coding transcript_biotype:protein_coding
Length=447

 Score = 20.3 bits (39),  Expect = 392
 Identities = 6/8 (75%), Positives = 7/8 (88%), Gaps = 0/8 (0%)

Query  2    EALAFNPE  9
            EAL FNP+
Sbjct  283  EALQFNPQ  290


>EG:HORVU3Hr1G113740.28 pep chromosome:IBSC_v2:chr3H:688991150:688997849:1
gene:HORVU3Hr1G113740 transcript:HORVU3Hr1G113740.28
gene_biotype:protein_coding transcript_biotype:protein_coding
Length=285

 Score = 20.3 bits (39),  Expect = 392
 Identities = 6/8 (75%), Positives = 7/8 (88%), Gaps = 0/8 (0%)

Query  2    EALAFNPE  9
            EAL FNP+
Sbjct  136  EALQFNPQ  143
```

```
>EG:HORVU3Hr1G113740.22 pep chromosome:IBSC_v2:chr3H:688987578:688999413:1
gene:HORVU3Hr1G113740 transcript:HORVU3Hr1G113740.22
gene_biotype:protein_coding transcript_biotype:protein_coding
Length=300

 Score = 20.3 bits (39),  Expect = 392
 Identities = 6/8 (75%), Positives = 7/8 (88%), Gaps = 0/8 (0%)

Query  2    EALAFNPE  9
            EAL FNP+
Sbjct  136  EALQFNPQ  143


>EG:HORVU3Hr1G113740.18 pep chromosome:IBSC_v2:chr3H:688984499:688999387:1
gene:HORVU3Hr1G113740 transcript:HORVU3Hr1G113740.18
gene_biotype:protein_coding transcript_biotype:protein_coding
Length=447

 Score = 20.3 bits (39),  Expect = 392
 Identities = 6/8 (75%), Positives = 7/8 (88%), Gaps = 0/8 (0%)

Query  2    EALAFNPE  9
            EAL FNP+
Sbjct  283  EALQFNPQ  290


>EG:HORVU3Hr1G113740.4 pep chromosome:IBSC_v2:chr3H:688984375:688997895:1
gene:HORVU3Hr1G113740 transcript:HORVU3Hr1G113740.4
gene_biotype:protein_coding transcript_biotype:protein_coding
Length=753

 Score = 20.3 bits (39),  Expect = 392
 Identities = 6/8 (75%), Positives = 7/8 (88%), Gaps = 0/8 (0%)

Query  2    EALAFNPE  9
            EAL FNP+
Sbjct  604  EALQFNPQ  611


>EG:HORVU3Hr1G113740.24 pep chromosome:IBSC_v2:chr3H:688987864:688999426:1
gene:HORVU3Hr1G113740 transcript:HORVU3Hr1G113740.24
gene_biotype:protein_coding transcript_biotype:protein_coding
Length=300

 Score = 20.3 bits (39),  Expect = 392
 Identities = 6/8 (75%), Positives = 7/8 (88%), Gaps = 0/8 (0%)

Query  2    EALAFNPE  9
            EAL FNP+
Sbjct  136  EALQFNPQ  143


>EG:HORVU3Hr1G113740.1 pep chromosome:IBSC_v2:chr3H:688984300:688999413:1
gene:HORVU3Hr1G113740 transcript:HORVU3Hr1G113740.1
gene_biotype:protein_coding transcript_biotype:protein_coding
Length=768

 Score = 20.3 bits (39),  Expect = 392
```

```
 Identities = 6/8 (75%), Positives = 7/8 (88%), Gaps = 0/8 (0%)

Query  2    EALAFNPE  9
            EAL FNP+
Sbjct  604  EALQFNPQ  611


>EG:HORVU3Hr1G113740.3 pep chromosome:IBSC_v2:chr3H:688984329:688999413:1
gene:HORVU3Hr1G113740 transcript:HORVU3Hr1G113740.3
gene_biotype:protein_coding transcript_biotype:protein_coding
Length=768

 Score = 20.3 bits (39),  Expect = 392
 Identities = 6/8 (75%), Positives = 7/8 (88%), Gaps = 0/8 (0%)

Query  2    EALAFNPE  9
            EAL FNP+
Sbjct  604  EALQFNPQ  611


>EG:HORVU3Hr1G113740.8 pep chromosome:IBSC_v2:chr3H:688984419:688999415:1
gene:HORVU3Hr1G113740 transcript:HORVU3Hr1G113740.8
gene_biotype:protein_coding transcript_biotype:protein_coding
Length=447

 Score = 20.3 bits (39),  Expect = 392
 Identities = 6/8 (75%), Positives = 7/8 (88%), Gaps = 0/8 (0%)

Query  2    EALAFNPE  9
            EAL FNP+
Sbjct  283  EALQFNPQ  290


>EG:HORVU3Hr1G113740.13 pep chromosome:IBSC_v2:chr3H:688984457:688999477:1
gene:HORVU3Hr1G113740 transcript:HORVU3Hr1G113740.13
gene_biotype:protein_coding transcript_biotype:protein_coding
Length=293

 Score = 20.3 bits (39),  Expect = 392
 Identities = 6/8 (75%), Positives = 7/8 (88%), Gaps = 0/8 (0%)

Query  2    EALAFNPE  9
            EAL FNP+
Sbjct  129  EALQFNPQ  136


>EG:HORVU3Hr1G113740.26 pep chromosome:IBSC_v2:chr3H:688988568:688999453:1
gene:HORVU3Hr1G113740 transcript:HORVU3Hr1G113740.26
gene_biotype:protein_coding transcript_biotype:protein_coding
Length=447

 Score = 20.3 bits (39),  Expect = 392
 Identities = 6/8 (75%), Positives = 7/8 (88%), Gaps = 0/8 (0%)

Query  2    EALAFNPE  9
            EAL FNP+
Sbjct  283  EALQFNPQ  290
```

```
>EG:HORVU3Hr1G113740.15 pep chromosome:IBSC_v2:chr3H:688984479:688999469:1
gene:HORVU3Hr1G113740 transcript:HORVU3Hr1G113740.15
gene_biotype:protein_coding transcript_biotype:protein_coding
Length=768

 Score = 20.3 bits (39),  Expect = 392
 Identities = 6/8 (75%), Positives = 7/8 (88%), Gaps = 0/8 (0%)

Query  2    EALAFNPE  9
            EAL FNP+
Sbjct  604  EALQFNPQ  611


>EG:HORVU3Hr1G113740.16 pep chromosome:IBSC_v2:chr3H:688984479:688999469:1
gene:HORVU3Hr1G113740 transcript:HORVU3Hr1G113740.16
gene_biotype:protein_coding transcript_biotype:protein_coding
Length=769

 Score = 20.3 bits (39),  Expect = 392
 Identities = 6/8 (75%), Positives = 7/8 (88%), Gaps = 0/8 (0%)

Query  2    EALAFNPE  9
            EAL FNP+
Sbjct  605  EALQFNPQ  612


>EG:HORVU3Hr1G113740.5 pep chromosome:IBSC_v2:chr3H:688984375:688999451:1
gene:HORVU3Hr1G113740 transcript:HORVU3Hr1G113740.5
gene_biotype:protein_coding transcript_biotype:protein_coding
Length=300

 Score = 20.3 bits (39),  Expect = 392
 Identities = 6/8 (75%), Positives = 7/8 (88%), Gaps = 0/8 (0%)

Query  2    EALAFNPE  9
            EAL FNP+
Sbjct  136  EALQFNPQ  143


>EG:HORVU3Hr1G113740.25 pep chromosome:IBSC_v2:chr3H:688988343:688999413:1
gene:HORVU3Hr1G113740 transcript:HORVU3Hr1G113740.25
gene_biotype:protein_coding transcript_biotype:protein_coding
Length=300

 Score = 20.3 bits (39),  Expect = 392
 Identities = 6/8 (75%), Positives = 7/8 (88%), Gaps = 0/8 (0%)

Query  2    EALAFNPE  9
            EAL FNP+
Sbjct  136  EALQFNPQ  143


>EG:HORVU3Hr1G113740.21 pep chromosome:IBSC_v2:chr3H:688986131:688999453:1
gene:HORVU3Hr1G113740 transcript:HORVU3Hr1G113740.21
gene_biotype:protein_coding transcript_biotype:protein_coding
Length=636

 Score = 20.3 bits (39),  Expect = 392
 Identities = 6/8 (75%), Positives = 7/8 (88%), Gaps = 0/8 (0%)
```

```
Query  2    EALAFNPE  9
            EAL FNP+
Sbjct  604  EALQFNPQ  611


>EG:HORVU6Hr1G077520.1 pep chromosome:IBSC_v2:chr6H:530750660:530751875:-1
gene:HORVU6Hr1G077520 transcript:HORVU6Hr1G077520.1
gene_biotype:protein_coding transcript_biotype:protein_coding
description:Predicted protein [Source:UniProtKB/TrEMBL;Acc:F2EI00]
Length=104

 Score = 20.3 bits (39),  Expect = 392
 Identities = 6/10 (60%), Positives = 7/10 (70%), Gaps = 0/10 (0%)

Query  1   REALAFNPEY  10
           R  LA+ PEY
Sbjct  57  RRVLAYKPEY  66


>EG:HORVU7Hr1G035550.3 pep chromosome:IBSC_v2:chr7H:79247352:79248434:1
gene:HORVU7Hr1G035550 transcript:HORVU7Hr1G035550.3
gene_biotype:protein_coding transcript_biotype:protein_coding
Length=318

 Score = 20.3 bits (39),  Expect = 392
 Identities = 6/10 (60%), Positives = 7/10 (70%), Gaps = 0/10 (0%)

Query  3   ALAFNPEYQQ  12
           +L  NP YQQ
Sbjct  47  SLFLNPAYQQ  56


>EG:HORVU7Hr1G035550.1 pep chromosome:IBSC_v2:chr7H:79246546:79248297:1
gene:HORVU7Hr1G035550 transcript:HORVU7Hr1G035550.1
gene_biotype:protein_coding transcript_biotype:protein_coding
Length=535

 Score = 20.3 bits (39),  Expect = 392
 Identities = 6/10 (60%), Positives = 7/10 (70%), Gaps = 0/10 (0%)

Query  3    ALAFNPEYQQ  12
            +L  NP YQQ
Sbjct  267  SLFLNPAYQQ  276


>EG:HORVU7Hr1G035550.2 pep chromosome:IBSC_v2:chr7H:79246549:79248309:1
gene:HORVU7Hr1G035550 transcript:HORVU7Hr1G035550.2
gene_biotype:protein_coding transcript_biotype:protein_coding
Length=535

 Score = 20.3 bits (39),  Expect = 392
 Identities = 6/10 (60%), Positives = 7/10 (70%), Gaps = 0/10 (0%)

Query  3    ALAFNPEYQQ  12
            +L  NP YQQ
Sbjct  264  SLFLNPAYQQ  273
```

```
>EG:HORVU4Hr1G040280.4 pep chromosome:IBSC_v2:chr4H:309109269:309113323:-1
gene:HORVU4Hr1G040280 transcript:HORVU4Hr1G040280.4
gene_biotype:protein_coding transcript_biotype:protein_coding
description:Eukaryotic translation initiation factor
3 subunit I [Source:UniProtKB/TrEMBL;Acc:A0A287NVB1]
Length=339

 Score = 19.8 bits (38),  Expect = 538
 Identities = 6/7 (86%), Positives = 7/7 (100%), Gaps = 0/7 (0%)

Query  3    ALAFNPE  9
            ALAFNP+
Sbjct  293  ALAFNPD  299


>EG:HORVU4Hr1G040280.3 pep chromosome:IBSC_v2:chr4H:309109196:309113599:-1
gene:HORVU4Hr1G040280 transcript:HORVU4Hr1G040280.3
gene_biotype:protein_coding transcript_biotype:protein_coding
description:Eukaryotic translation initiation factor
3 subunit I [Source:UniProtKB/TrEMBL;Acc:A0A287NVB1]
Length=326

 Score = 19.8 bits (38),  Expect = 538
 Identities = 6/7 (86%), Positives = 7/7 (100%), Gaps = 0/7 (0%)

Query  3    ALAFNPE  9
            ALAFNP+
Sbjct  293  ALAFNPD  299


>EG:HORVU4Hr1G040280.2 pep chromosome:IBSC_v2:chr4H:309109182:309113038:-1
gene:HORVU4Hr1G040280 transcript:HORVU4Hr1G040280.2
gene_biotype:protein_coding transcript_biotype:protein_coding
description:Eukaryotic translation initiation factor
3 subunit I [Source:UniProtKB/TrEMBL;Acc:A0A287NVB1]
Length=218

 Score = 19.8 bits (38),  Expect = 538
 Identities = 6/7 (86%), Positives = 7/7 (100%), Gaps = 0/7 (0%)

Query  3    ALAFNPE  9
            ALAFNP+
Sbjct  185  ALAFNPD  191


>EG:HORVU4Hr1G055900.2 pep chromosome:IBSC_v2:chr4H:469810729:469812353:-1
gene:HORVU4Hr1G055900 transcript:HORVU4Hr1G055900.2
gene_biotype:protein_coding transcript_biotype:protein_coding
Length=283

 Score = 19.8 bits (38),  Expect = 538
 Identities = 6/10 (60%), Positives = 8/10 (80%), Gaps = 0/10 (0%)

Query  2    EALAFNPEYQ  11
            +ALAF P+ Q
Sbjct  223  QALAFKPDLQ  232


>EG:HORVU4Hr1G055900.6 pep chromosome:IBSC_v2:chr4H:469811093:469815218:-1
```

```
gene:HORVU4Hr1G055900 transcript:HORVU4Hr1G055900.6
gene_biotype:protein_coding transcript_biotype:protein_coding
Length=963

 Score = 19.8 bits (38),  Expect = 538
 Identities = 6/10 (60%), Positives = 8/10 (80%), Gaps = 0/10 (0%)

Query  2    EALAFNPEYQ  11
            +ALAF P+ Q
Sbjct  903  QALAFKPDLQ  912


>EG:HORVU4Hr1G055900.8 pep chromosome:IBSC_v2:chr4H:469811093:469815218:-1
gene:HORVU4Hr1G055900 transcript:HORVU4Hr1G055900.8
gene_biotype:protein_coding transcript_biotype:protein_coding
Length=966

 Score = 19.8 bits (38),  Expect = 538
 Identities = 6/10 (60%), Positives = 8/10 (80%), Gaps = 0/10 (0%)

Query  2    EALAFNPEYQ  11
            +ALAF P+ Q
Sbjct  906  QALAFKPDLQ  915


>EG:HORVU4Hr1G055900.1 pep chromosome:IBSC_v2:chr4H:469810551:469813858:-1
gene:HORVU4Hr1G055900 transcript:HORVU4Hr1G055900.1
gene_biotype:protein_coding transcript_biotype:protein_coding
Length=639

 Score = 19.8 bits (38),  Expect = 538
 Identities = 6/10 (60%), Positives = 8/10 (80%), Gaps = 0/10 (0%)

Query  2    EALAFNPEYQ  11
            +ALAF P+ Q
Sbjct  579  QALAFKPDLQ  588


>EG:HORVU4Hr1G055900.7 pep chromosome:IBSC_v2:chr4H:469811093:469815218:-1
gene:HORVU4Hr1G055900 transcript:HORVU4Hr1G055900.7
gene_biotype:protein_coding transcript_biotype:protein_coding
Length=965

 Score = 19.8 bits (38),  Expect = 538
 Identities = 6/10 (60%), Positives = 8/10 (80%), Gaps = 0/10 (0%)

Query  2    EALAFNPEYQ  11
            +ALAF P+ Q
Sbjct  905  QALAFKPDLQ  914


>EG:HORVU4Hr1G055900.5 pep chromosome:IBSC_v2:chr4H:469811093:469815218:-1
gene:HORVU4Hr1G055900 transcript:HORVU4Hr1G055900.5
gene_biotype:protein_coding transcript_biotype:protein_coding
Length=967

 Score = 19.8 bits (38),  Expect = 538
 Identities = 6/10 (60%), Positives = 8/10 (80%), Gaps = 0/10 (0%)
```

```
Query  2    EALAFNPEYQ  11
            +ALAF P+ Q
Sbjct  907  QALAFKPDLQ  916


>EG:HORVU3Hr1G099420.2 pep chromosome:IBSC_v2:chr3H:661498454:661499433:-1
gene:HORVU3Hr1G099420 transcript:HORVU3Hr1G099420.2
gene_biotype:protein_coding transcript_biotype:protein_coding
Length=179

 Score = 19.8 bits (38),  Expect = 538
 Identities = 6/9 (67%), Positives = 6/9 (67%), Gaps = 0/9 (0%)

Query  2   EALAFNPEY  10
           EAL  NP Y
Sbjct  21  EALGLNPSY  29


>EG:HORVU3Hr1G099420.1 pep chromosome:IBSC_v2:chr3H:661498454:661499397:-1
gene:HORVU3Hr1G099420 transcript:HORVU3Hr1G099420.1
gene_biotype:protein_coding transcript_biotype:protein_coding
Length=179

 Score = 19.8 bits (38),  Expect = 538
 Identities = 6/9 (67%), Positives = 6/9 (67%), Gaps = 0/9 (0%)

Query  2   EALAFNPEY  10
           EAL  NP Y
Sbjct  21  EALGLNPSY  29


>EG:HORVU3Hr1G099420.3 pep chromosome:IBSC_v2:chr3H:661498454:661499436:-1
gene:HORVU3Hr1G099420 transcript:HORVU3Hr1G099420.3
gene_biotype:protein_coding transcript_biotype:protein_coding
Length=191

 Score = 19.8 bits (38),  Expect = 538
 Identities = 6/9 (67%), Positives = 6/9 (67%), Gaps = 0/9 (0%)

Query  2   EALAFNPEY  10
           EAL  NP Y
Sbjct  33  EALGLNPSY  41


>EG:HORVU7Hr1G035590.4 pep chromosome:IBSC_v2:chr7H:79324864:79326481:1
gene:HORVU7Hr1G035590 transcript:HORVU7Hr1G035590.4
gene_biotype:protein_coding transcript_biotype:protein_coding
Length=501

 Score = 19.8 bits (38),  Expect = 538
 Identities = 6/10 (60%), Positives = 7/10 (70%), Gaps = 0/10 (0%)

Query  3    ALAFNPEYQQ  12
            AL  NP YQ+
Sbjct  245  ALFLNPAYQE  254


>EG:HORVU7Hr1G035590.2 pep chromosome:IBSC_v2:chr7H:79324751:79326481:1
gene:HORVU7Hr1G035590 transcript:HORVU7Hr1G035590.2
```

```
gene_biotype:protein_coding transcript_biotype:protein_coding
Length=501

 Score = 19.8 bits (38),  Expect = 538
 Identities = 6/10 (60%), Positives = 7/10 (70%), Gaps = 0/10 (0%)

Query  3    ALAFNPEYQQ  12
            AL  NP YQ+
Sbjct  245  ALFLNPAYQE  254


>EG:HORVU7Hr1G035590.3 pep chromosome:IBSC_v2:chr7H:79324845:79326487:1
gene:HORVU7Hr1G035590 transcript:HORVU7Hr1G035590.3
gene_biotype:protein_coding transcript_biotype:protein_coding
Length=509

 Score = 19.8 bits (38),  Expect = 538
 Identities = 6/10 (60%), Positives = 7/10 (70%), Gaps = 0/10 (0%)

Query  3    ALAFNPEYQQ  12
            AL  NP YQ+
Sbjct  252  ALFLNPAYQE  261


>EG:HORVU7Hr1G035590.1 pep chromosome:IBSC_v2:chr7H:79324382:79326849:1
gene:HORVU7Hr1G035590 transcript:HORVU7Hr1G035590.1
gene_biotype:protein_coding transcript_biotype:protein_coding
Length=576

 Score = 19.8 bits (38),  Expect = 538
 Identities = 6/10 (60%), Positives = 7/10 (70%), Gaps = 0/10 (0%)

Query  3    ALAFNPEYQQ  12
            AL  NP YQ+
Sbjct  319  ALFLNPAYQE  328


>EG:HORVU1Hr1G074650.14 pep chromosome:IBSC_v2:chr1H:508772354:508778739:-1
gene:HORVU1Hr1G074650 transcript:HORVU1Hr1G074650.14
gene_biotype:protein_coding transcript_biotype:protein_coding
description:Predicted protein [Source:UniProtKB/TrEMBL;Acc:F2E3N3]
Length=318

 Score = 19.8 bits (38),  Expect = 538
 Identities = 7/11 (64%), Positives = 8/11 (73%), Gaps = 0/11 (0%)

Query  1   REALAFNPEYQ  11
           R ALAF P+ Q
Sbjct  61  RVALAFHPDSQ  71


>EG:HORVU1Hr1G074650.15 pep chromosome:IBSC_v2:chr1H:508772354:508778739:-1
gene:HORVU1Hr1G074650 transcript:HORVU1Hr1G074650.15
gene_biotype:protein_coding transcript_biotype:protein_coding
description:Predicted protein [Source:UniProtKB/TrEMBL;Acc:F2E3N3]
Length=324

 Score = 19.8 bits (38),  Expect = 538
 Identities = 7/11 (64%), Positives = 8/11 (73%), Gaps = 0/11 (0%)
```

```
Query  1   REALAFNPEYQ  11
           R ALAF P+ Q
Sbjct  61  RVALAFHPDSQ  71


>EG:HORVU1Hr1G074650.21 pep chromosome:IBSC_v2:chr1H:508772366:508778454:-1
gene:HORVU1Hr1G074650 transcript:HORVU1Hr1G074650.21
gene_biotype:protein_coding transcript_biotype:protein_coding
description:Predicted protein [Source:UniProtKB/TrEMBL;Acc:F2E3N3]
Length=554

 Score = 19.8 bits (38),  Expect = 538
 Identities = 7/11 (64%), Positives = 8/11 (73%), Gaps = 0/11 (0%)

Query  1   REALAFNPEYQ  11
           R ALAF P+ Q
Sbjct  23  RVALAFHPDSQ  33


>EG:HORVU1Hr1G074650.3 pep chromosome:IBSC_v2:chr1H:508771004:508778386:-1
gene:HORVU1Hr1G074650 transcript:HORVU1Hr1G074650.3
gene_biotype:protein_coding transcript_biotype:protein_coding
description:Predicted protein [Source:UniProtKB/TrEMBL;Acc:F2E3N3]
Length=381

 Score = 19.8 bits (38),  Expect = 538
 Identities = 7/11 (64%), Positives = 8/11 (73%), Gaps = 0/11 (0%)

Query  1   REALAFNPEYQ  11
           R ALAF P+ Q
Sbjct  1   RVALAFHPDSQ  11


>EG:HORVU1Hr1G074650.28 pep chromosome:IBSC_v2:chr1H:508772496:508778560:-1
gene:HORVU1Hr1G074650 transcript:HORVU1Hr1G074650.28
gene_biotype:protein_coding transcript_biotype:protein_coding
description:Predicted protein [Source:UniProtKB/TrEMBL;Acc:F2E3N3]
Length=580

 Score = 19.8 bits (38),  Expect = 538
 Identities = 7/11 (64%), Positives = 8/11 (73%), Gaps = 0/11 (0%)

Query  1   REALAFNPEYQ  11
           R ALAF P+ Q
Sbjct  59  RVALAFHPDSQ  69


>EG:HORVU1Hr1G074650.13 pep chromosome:IBSC_v2:chr1H:508772348:508778761:-1
gene:HORVU1Hr1G074650 transcript:HORVU1Hr1G074650.13
gene_biotype:protein_coding transcript_biotype:protein_coding
description:Predicted protein [Source:UniProtKB/TrEMBL;Acc:F2E3N3]
Length=318

 Score = 19.8 bits (38),  Expect = 538
 Identities = 7/11 (64%), Positives = 8/11 (73%), Gaps = 0/11 (0%)

Query  1   REALAFNPEYQ  11
           R ALAF P+ Q
```

```
Sbjct  61  RVALAFHPDSQ  71


>EG:HORVU1Hr1G074650.8 pep chromosome:IBSC_v2:chr1H:508772331:508778410:-1
gene:HORVU1Hr1G074650 transcript:HORVU1Hr1G074650.8
gene_biotype:protein_coding transcript_biotype:protein_coding
description:Predicted protein [Source:UniProtKB/TrEMBL;Acc:F2E3N3]
Length=387

 Score = 19.8 bits (38),  Expect = 538
 Identities = 7/11 (64%), Positives = 8/11 (73%), Gaps = 0/11 (0%)

Query  1   REALAFNPEYQ  11
           R ALAF P+ Q
Sbjct  9   RVALAFHPDSQ  19


>EG:HORVU1Hr1G074650.34 pep chromosome:IBSC_v2:chr1H:508773588:508778575:-1
gene:HORVU1Hr1G074650 transcript:HORVU1Hr1G074650.34
gene_biotype:protein_coding transcript_biotype:protein_coding
description:Predicted protein [Source:UniProtKB/TrEMBL;Acc:F2E3N3]
Length=412

 Score = 19.8 bits (38),  Expect = 538
 Identities = 7/11 (64%), Positives = 8/11 (73%), Gaps = 0/11 (0%)

Query  1   REALAFNPEYQ  11
           R ALAF P+ Q
Sbjct  64  RVALAFHPDSQ  74



Lambda      K        H
   0.316    0.177    0.673

Gapped
Lambda      K        H
   -1.00    -1.00    -1.00

Effective search space used: 503277330


  Database: Hordeum_vulgare.IBSC_v2.pep.all
    Posted date:  Jan 26, 2021  2:52 PM
  Number of letters in database: 85,297,361
  Number of sequences in database:  236,301



Matrix: BLOSUM90
Neighboring words threshold: 11
Window for multiple hits: 40
```
