# Supplementary material for: CRISPR/Cas9-Induced Loss-of-Function Mutation in the Barley Mitogen-Activated Protein Kinase 6 Gene Causes Abnormal Embryo Development Leading to Severely Reduced Grain Germination and Seedling Shootless Phenotype
Source: Front Plant Sci. 2021 Jul 30;12:670302. doi: 10.3389/fpls.2021.670302 (PMC8361755; doi:10.3389/fpls.2021.670302)
Supplement: Data Sheet 2 — Alignment of the 344-bp nucleotide sequence covering the CABVVH010000001.1 215.57 Mbp off-target site with the respective.ab1 files of 12 analyzed T3-generation plants. [file Data_Sheet_3.PDF]

10 20 30 40 50 60 70 80 90

ATAAATGGATTGCGGGAGCTTCAACACTTTCATGGDHHTCTaTGGATCGAC-aAACCTTCCAGTTGCATCATATAGAACTTTTCCTGAAG

Ref..seq(1>344) ATAAATGGATTGCGGGAGCTTCAACACTTTGGTAGCATTCTATGGATCGACAAAACCTTCCAGTTGCATCATATAGAACTTTTCCTGAAG

1.ab1(1>312)

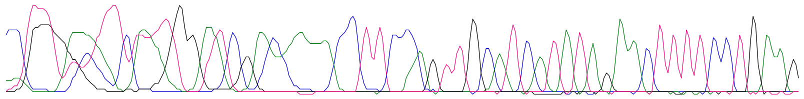

CATGGKM-TCW-TGGATCGAC-AAACCTTCCAGTTGCATCATATAGAACTTTTCCTGAAG

2.ab1(2>313)

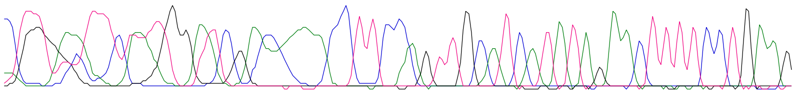

ATGGWMTTCW-TGGATCGAC--AACCTTCCAGTTGCATCATATAGAACTTTTCCTGAAG

3.ab1(1>314)

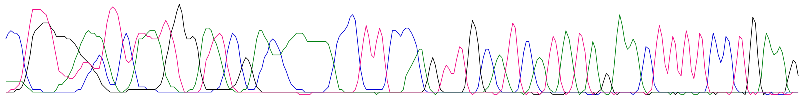

CATGGGWMTCTATGGATCGAC-AAACCTTCCAGTTGCATCATATAGAACTTTTCCTGAAG

4.ab1(2>313)

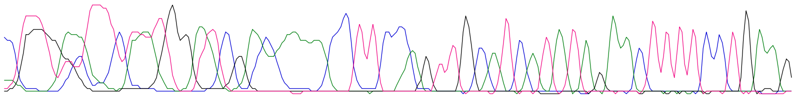

ATGGKMTTCTATGGATCGAC-AAACCTTCCAGTTGCATCATATAGAACTTTTCCTGAAG

5.ab1(1>313)

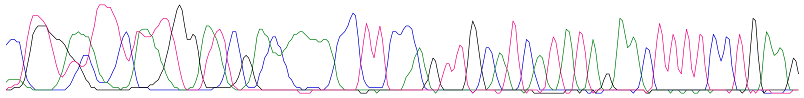

CATGGWMTTCW-TGGATCGAC-AAACCTTCCAGTTGCATCATATAGAACTTTTCCTGAAG

6.ab1(1>314)

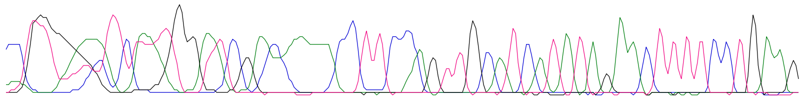

CATGG-WCTCW-TGGATCGAC-AAACCTTCCAGTTGCATCATATAGAACTTTTCCTGAAG

7.ab1(2>314)

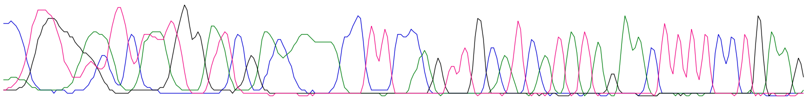

CATGGGWMTCTATGGATCGAC-AAACCTTCCAGTTGCATCATATAGAACTTTTCCTGAAG

8.ab1(1>313)

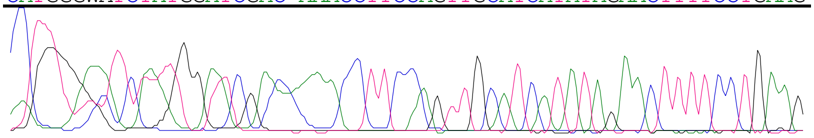

CATGG-WCTCW-TGGATCGAC--AACCTTCCAGTTGCATCATATAGAACTTTTCCTGAAG

9.ab1(2>299)

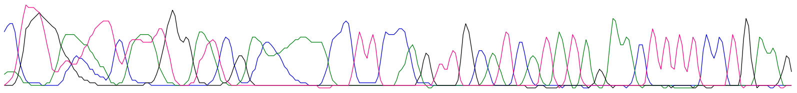

ATGGWM-TCTATGGATCGAC--AACCTTCCAGTTGCATCATATAGAACTTTTCCTGAAG

10.ab1(2>315)

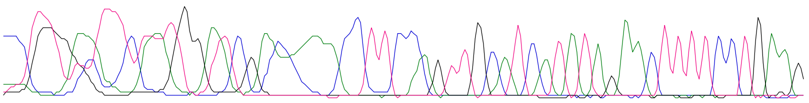

CATGGWMTTCTATGGATCGAC--AACCTTCCAGTTGCATCATATAGAACTTTTCCTGAAG

11.ab1(1>312)

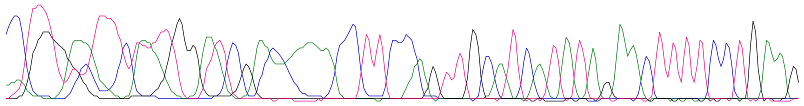

CATGGKCTTCW-TGGATCGAC--AACCTTCCAGTTGCATCATATAGAACTTTTCCTGAAG

12.ab1(1>312)

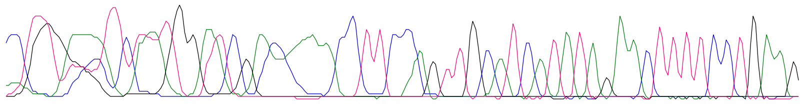

CATGGK-MTCTATGGATCGAC--AACCTTCCAGTTGCATCATATAGAACTTTTCCTGAAG

100 110 120 130 140 150 160 170 180

GAAACTATTAAGGAACGTTGTTTTGACATCCATCTGCCAAATTTCATAATCGAAAAATGCAGCTATTTCTAACATGATTCTGACAGACTT

Ref..seq(1>344) GAAACTATTAAGGAACGTTGTTTTGACATCCATCTGCCAAATTTCATAATCGAAAAATGCAGCTATTTCTAACATGATTCTGACAGACTT

1.ab1(1>312)

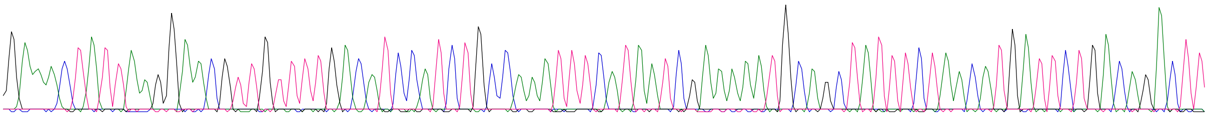

GAAACTATTAAGGAACGTTGTTTTGACATCCATCTGCCAAATTTCATAATCGAAAAATGCAGCTATTTCTAACATGATTCTGACAGACTT

2.ab1(2>313)

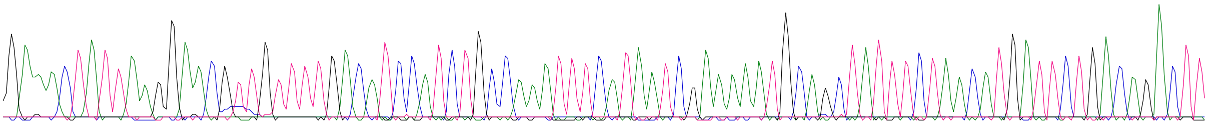

GAAACTATTAAGGAACGTTGTTTTGACATCCATCTGCCAAATTTCATAATCGAAAAATGCAGCTATTTCTAACATGATTCTGACAGACTT

3.ab1(1>314)

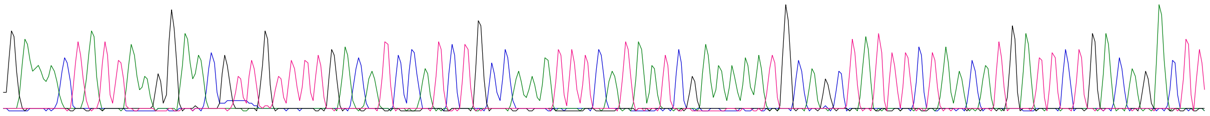

GAAACTATTAAGGAACGTTGTTTTGACATCCATCTGCCAAATTTCATAATCGAAAAATGCAGCTATTTCTAACATGATTCTGACAGACTT

4.ab1(2>313)

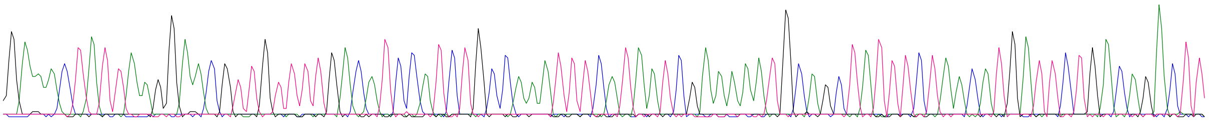

GAAACTATTAAGGAACGTTGTTTTGACATCCATCTGCCAAATTTCATAATCGAAAAATGCAGCTATTTCTAACATGATTCTGACAGACTT

5.ab1(1>313)

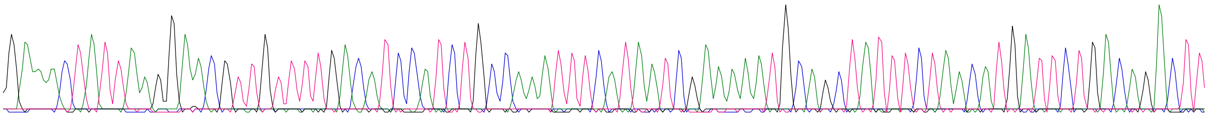

GAAACTATTAAGGAACGTTGTTTTGACATCCATCTGCCAAATTTCATAATCGAAAAATGCAGCTATTTCTAACATGATTCTGACAGACTT

6.ab1(1>314)

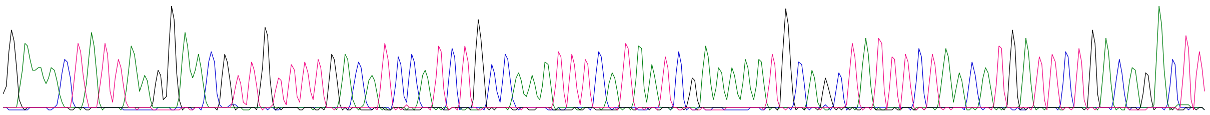

GAAACTATTAAGGAACGTTGTTTTGACATCCATCTGCCAAATTTCATAATCGAAAAATGCAGCTATTTCTAACATGATTCTGACAGACTT

7.ab1(2>314)

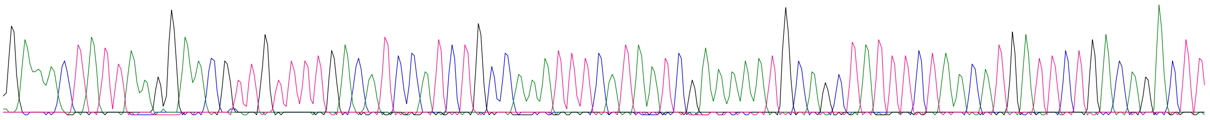

GAAACTATTAAGGAACGTTGTTTTGACATCCATCTGCCAAATTTCATAATCGAAAAATGCAGCTATTTCTAACATGATTCTGACAGACTT

8.ab1(1>313)

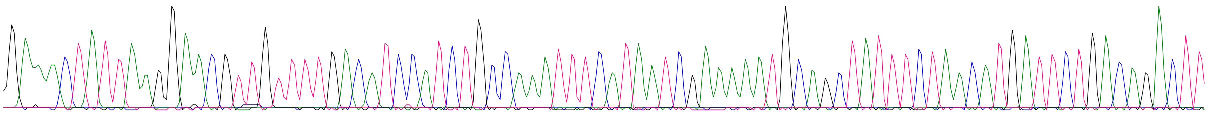

GAAACTATTAAGGAACGTTGTTTTGACATCCATCTGCCAAATTTCATAATCGAAAAATGCAGCTATTTCTAACATGATTCTGACAGACTT

9.ab1(2>299)

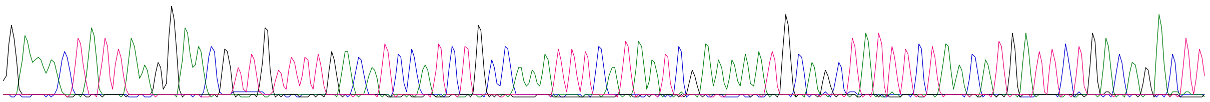

GAAACTATTAAGGAACGTTGTTTTGACATCCATCTGCCAAATTTCATAATCGAAAAATGCAGCTATTTCTAACATGATTCTGACAGACTT

10.ab1(2>315)

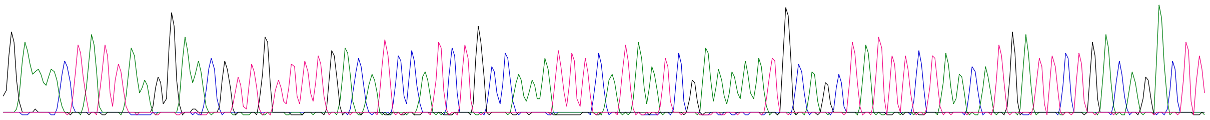

GAAACTATTAAGGAACGTTGTTTTGACATCCATCTGCCAAATTTCATAATCGAAAAATGCAGCTATTTCTAACATGATTCTGACAGACTT

11.ab1(1>312)

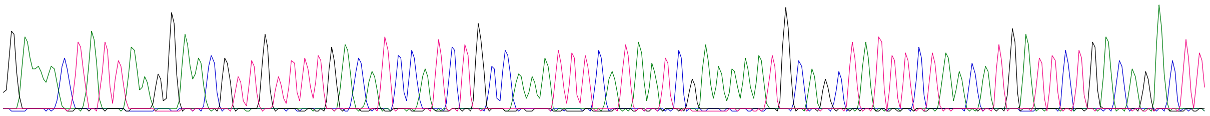

GAAACTATTAAGGAACGTTGTTTTGACATCCATCTGCCAAATTTCATAATCGAAAAATGCAGCTATTTCTAACATGATTCTGACAGACTT

12.ab1(1>312)

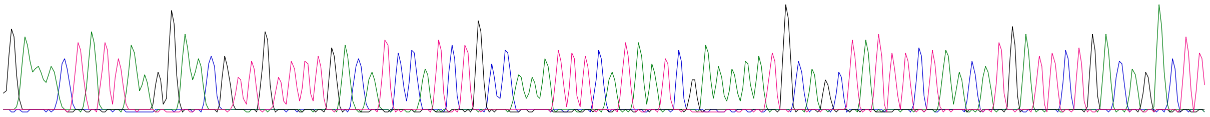

GAAACTATTAAGGAACGTTGTTTTGACATCCATCTGCCAAATTTCATAATCGAAAAATGCAGCTATTTCTAACATGATTCTGACAGACTT

190 200 210 220 230 240 250 260 270

AAGAATCGCTACGGGTGAGAAAGTCTCATCGTAGTCAAGTCCTTGAACTTGTGAAAAACTTTTTGCCACAAATCAAGCTTTATAGACGGT

Ref..seq(1>344) AAGAATCGCTACGGGTGAGAAAGTCTCATCGTAGTCAAGTCCTTGAACTTGTGAAAAACTTTTTGCCACAAATCAAGCTTTATAGACGGT

1.ab1(1>312)

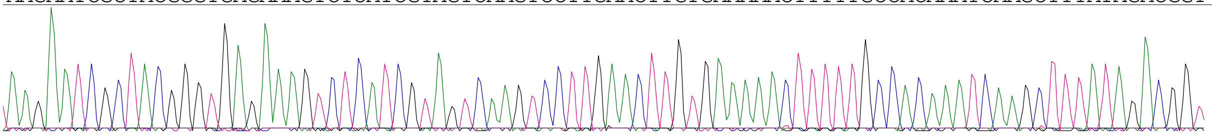

AAGAATCGCTACGGGTGAGAAAGTCTCATCGTAGTCAAGTCCTTGAACTTGTGAAAAACTTTTTGCCACAAATCAAGCTTTATAGACGGT

2.ab1(2>313)

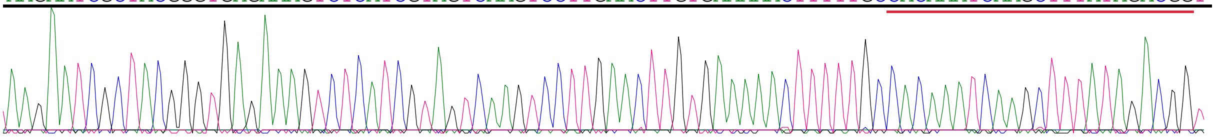

AAGAATCGCTACGGGTGAGAAAGTCTCATCGTAGTCAAGTCCTTGAACTTGTGAAAAACTTTTTGCCACAAATCAAGCTTTATAGACGGT

3.ab1(1>314)

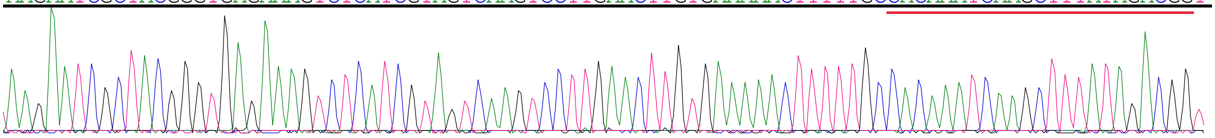

AAGAATCGCTACGGGTGAGAAAGTCTCATCGTAGTCAAGTCCTTGAACTTGTGAAAAACTTTTTGCCACAAATCAAGCTTTATAGACGGT

4.ab1(2>313)

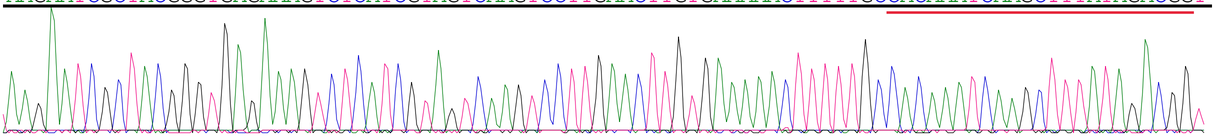

AAGAATCGCTACGGGTGAGAAAGTCTCATCGTAGTCAAGTCCTTGAACTTGTGAAAAACTTTTTGCCACAAATCAAGCTTTATAGACGGT

5.ab1(1>313)

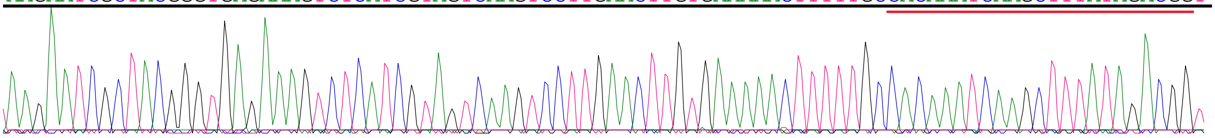

AAGAATCGCTACGGGTGAGAAAGTCTCATCGTAGTCAAGTCCTTGAACTTGTGAAAAACTTTTTGCCACAAATCAAGCTTTATAGACGGT

6.ab1(1>314)

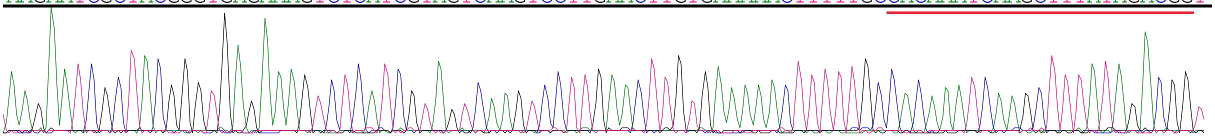

AAGAATCGCTACGGGTGAGAAAGTCTCATCGTAGTCAAGTCCTTGAACTTGTGAAAAACTTTTTGCCACAAATCAAGCTTTATAGACGGT

7.ab1(2>314)

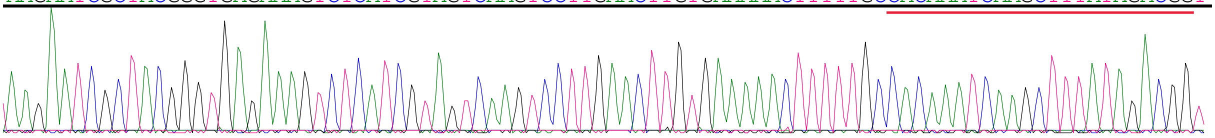

AAGAATCGCTACGGGTGAGAAAGTCTCATCGTAGTCAAGTCCTTGAACTTGTGAAAAACTTTTTGCCACAAATCAAGCTTTATAGACGGT

8.ab1(1>313)

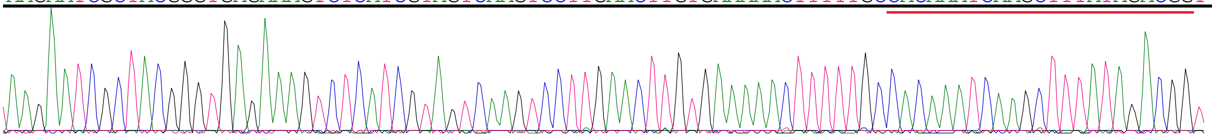

AAGAATCGCTACGGGTGAGAAAGTCTCATCGTAGTCAAGTCCTTGAACTTGTGAAAAACTTTTTGCCACAAATCAAGCTTTATAGACGGT

9.ab1(2>299)

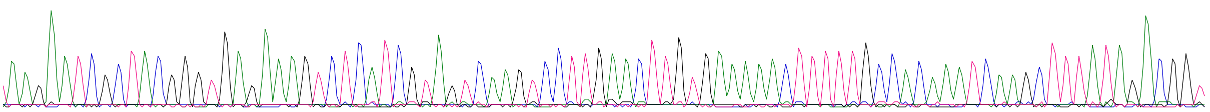

AAGAATCGCTACGGGTGAGAAAGTCTCATCGTAGTCAAGTCCTTGAACTTGTGAAAAACTTTTTGCCACAAATCAAGCTTTATAGACGGT

10.ab1(2>315)

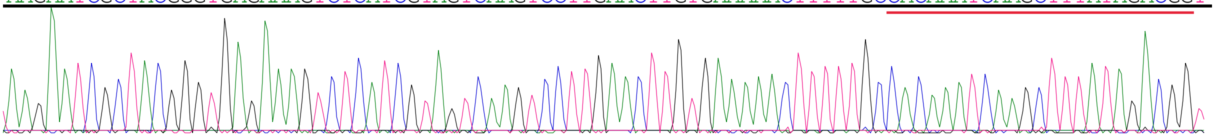

AAGAATCGCTACGGGTGAGAAAGTCTCATCGTAGTCAAGTCCTTGAACTTGTGAAAAACTTTTTGCCACAAATCAAGCTTTATAGACGGT

11.ab1(1>312)

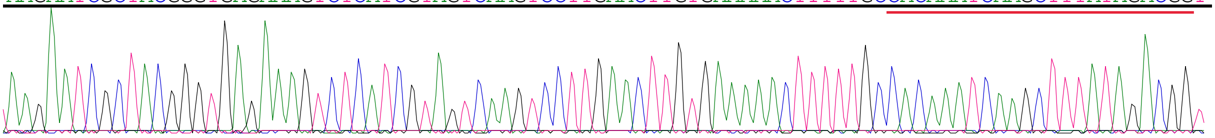

AAGAATCGCTACGGGTGAGAAAGTCTCATCGTAGTCAAGTCCTTGAACTTGTGAAAAACTTTTTGCCACAAATCAAGCTTTATAGACGGT

12.ab1(1>312)

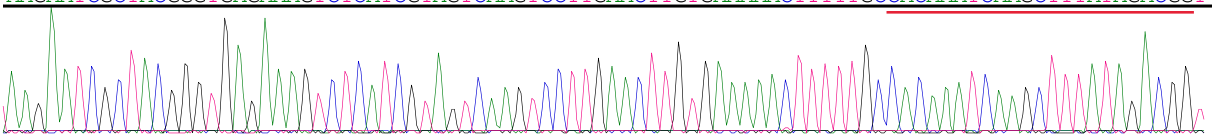

AAGAATCGCTACGGGTGAGAAAGTCTCATCGTAGTCAAGTCCTTGAACTTGTGAAAAACTTTTTGCCACAAATCAAGCTTTATAGACGGT

280 290 300 310 320 330 340

GACATTACCGTTGGCGTCTGTCTTTTCTTAATGATCCAATTATTCTGAATGTCCTTTCGCCCTtCAGG-TAGTACADA

Ref..seq(1>344) GACATTACCGTTGGCGTCTGTCTTTTCTTAATGATCCAATTATTCTGAATGTCCTTTCGCCCTTCAGG-TAGTAC

1.ab1(1>312)

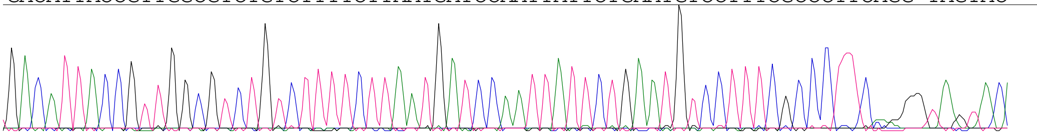

GACATTACCGTTGGCGTCTGTCTTTTCTTAATGATCCAATTATTCTGAATGTCCTTTCGCCCT-CAGGGTAGTACA

2.ab1(2>313)

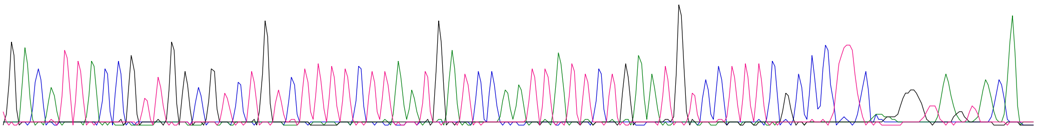

GACATTACCGTTGGCGTCTGTCTTTTCTTAATGATCCAATTATTCTGAATGTCCTTTCGCCCT-CAGG-TAGTACAAC

3.ab1(1>314)

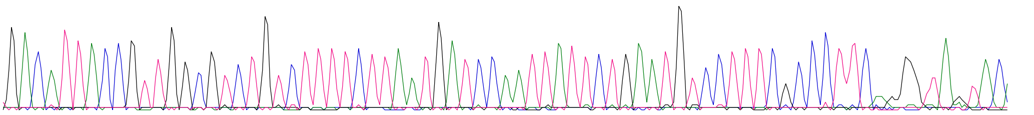

GACATTACCGTTGGCGTCTGTCTTTTCTTAATGATCCAATTATTCTGAATGTCCTTTCGCCCTTCAGG-TAGTACA

4.ab1(2>313)

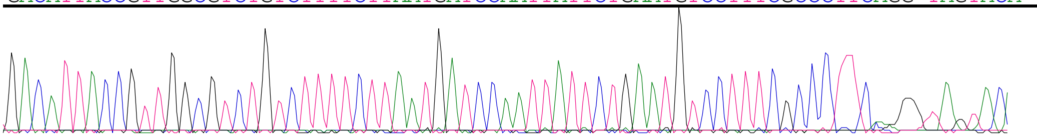

GACATTACCGTTGGCGTCTGTCTTTTCTTAATGATCCAATTATTCTGAATGTCCTTTCGCCCT-CAGG-TAGTACA

5.ab1(1>313)

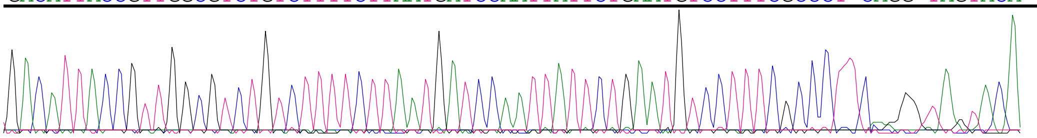

GACATTACCGTTGGCGTCTGTCTTTTCTTAATGATCCAATTATTCTGAATGTCCTTTCGCCCT-CAAG-TARTACAG

6.ab1(1>314)

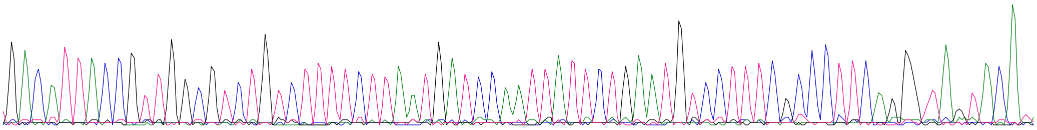

GACATTACCGTTGGCGTCTGTCTTTTCTTAATGATCCAATTATTCTGAATGTCCTTTCGCCCTTCAGG-TAGTACATA

7.ab1(2>314)

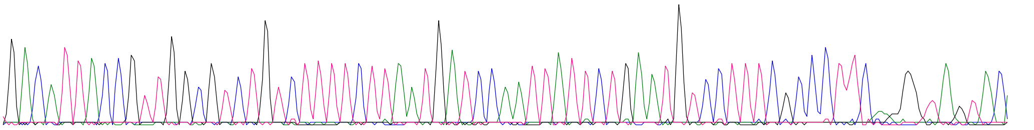

GACATTACCGTTGGCGTCTGTCTTTTCTTAATGATCCAATTATTCTGAATGTCCTTTCGCCCT-CAGG-TAGTACA

8.ab1(1>313)

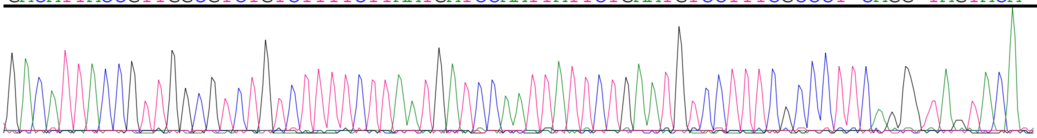

GACATTACCGTTGGCGTCTGTCTTTTCTTAATGATCCAATTATTCTGAATGTCCTTTCGCCCTTCAGG-TAGTACAAA

9.ab1(2>299)

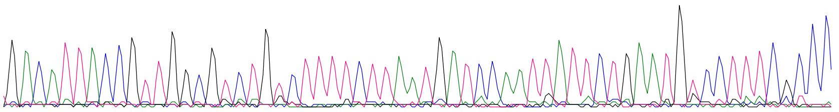

GACATTACCGTTGGCGTCTGKCTTTTCTTAATGATCCAATTATTCTGAATGTCCTTTCGCCC

10.ab1(2>315)

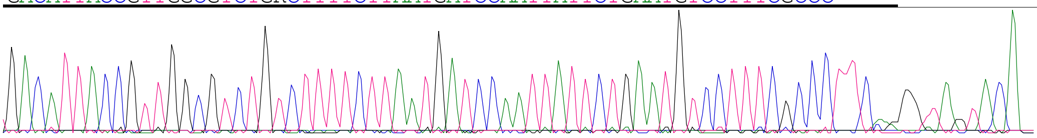

GACATTACCGTTGGCGTCTGTCTTTTCTTAATGATCCAATTATTCTGAATGTCCTTTCGCCCT-CARG-TAGTACAAA

11.ab1(1>312)

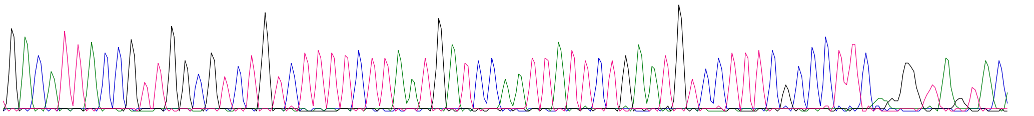

GACATTACCGTTGGCGTCTGTCTTTTCTTAATGATCCAATTATTCTGAATGTCCTTTCGCCCTTCAGG-TAGTACA

12.ab1(1>312)

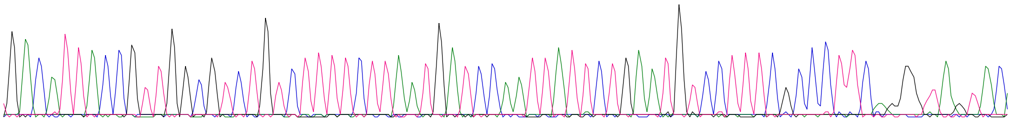

GACATTACCGTTGGCGTCTGTCTTTTCTTAATGATCCAATTATTCTGAATGTCCTTTCGCCCTTCAGG-TAGTACA
