# Supplementary figures and images for: CRISPR/Cas9-Induced Loss-of-Function Mutation in the Barley Mitogen-Activated Protein Kinase 6 Gene Causes Abnormal Embryo Development Leading to Severely Reduced Grain Germination and Seedling Shootless Phenotype
Source: Front Plant Sci. 2021 Jul 30;12:670302. doi: 10.3389/fpls.2021.670302 (PMC8361755; doi:10.3389/fpls.2021.670302)

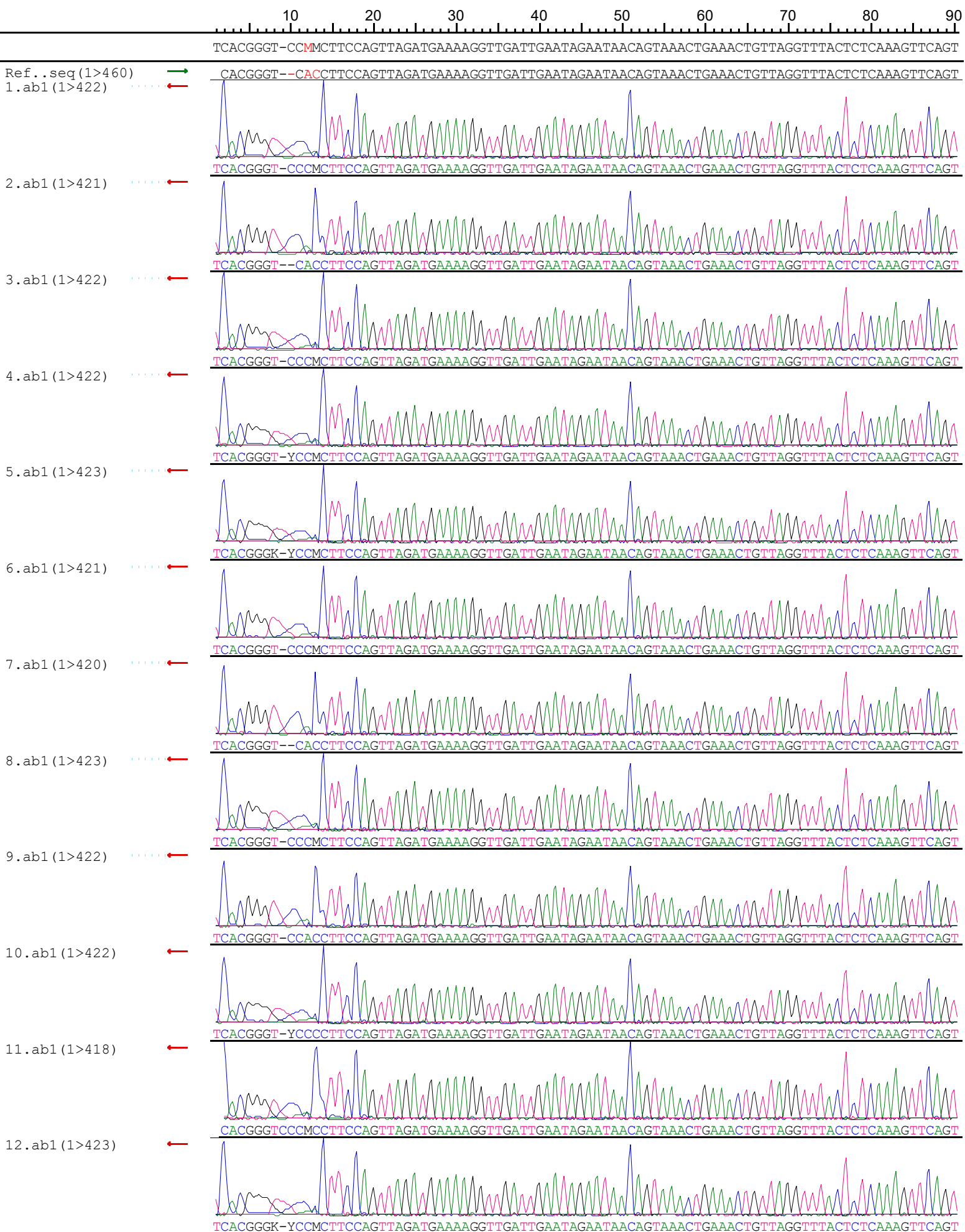

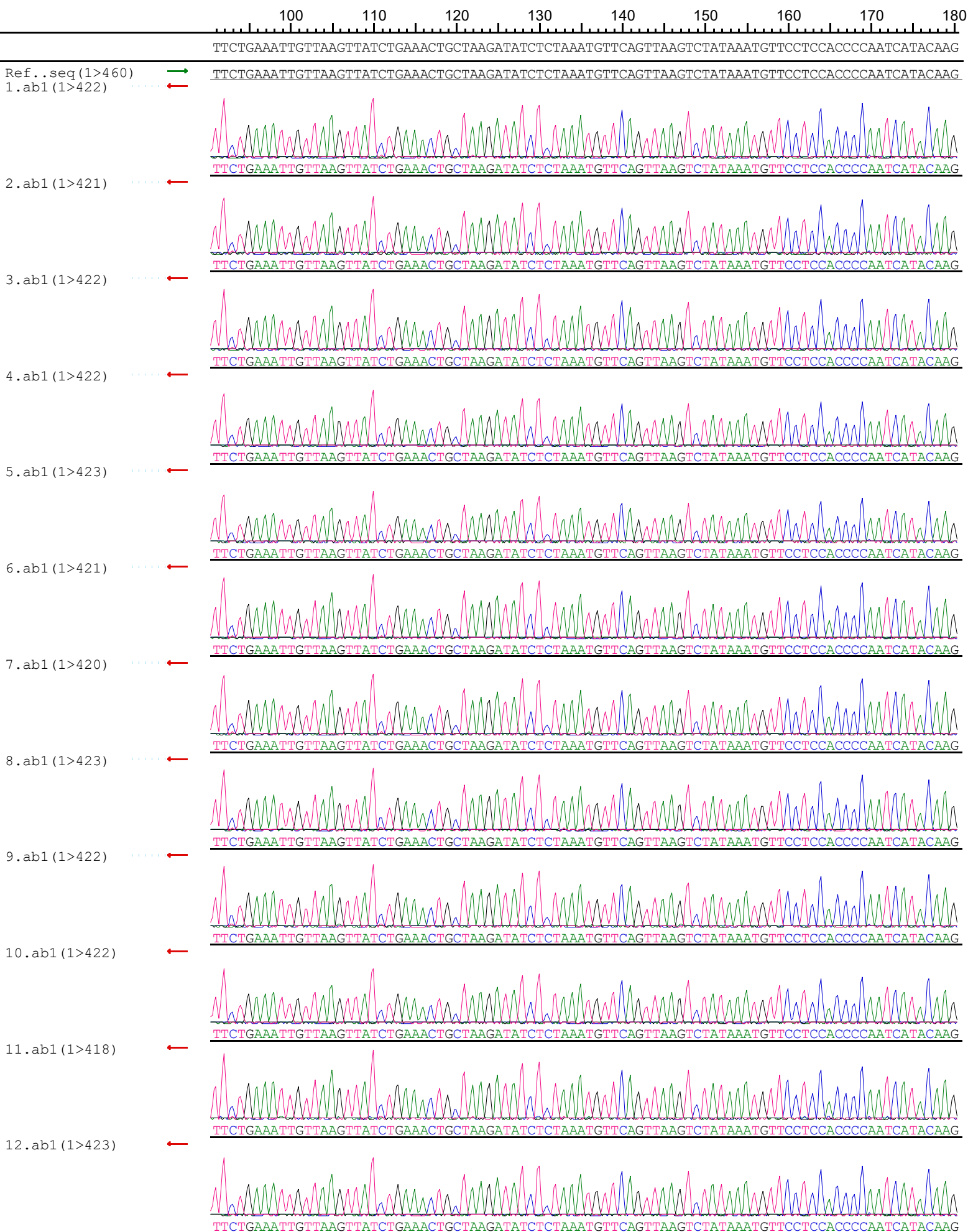

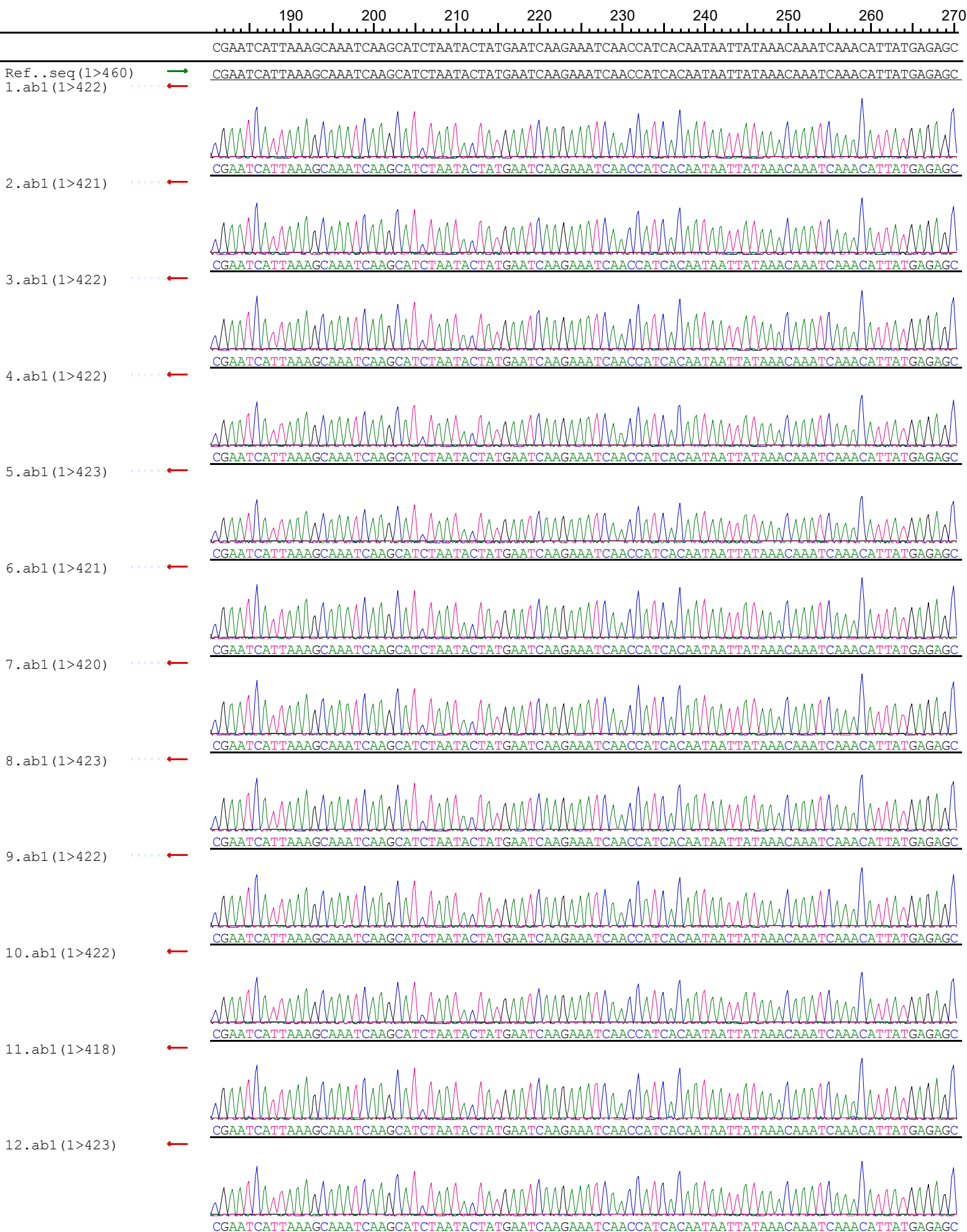

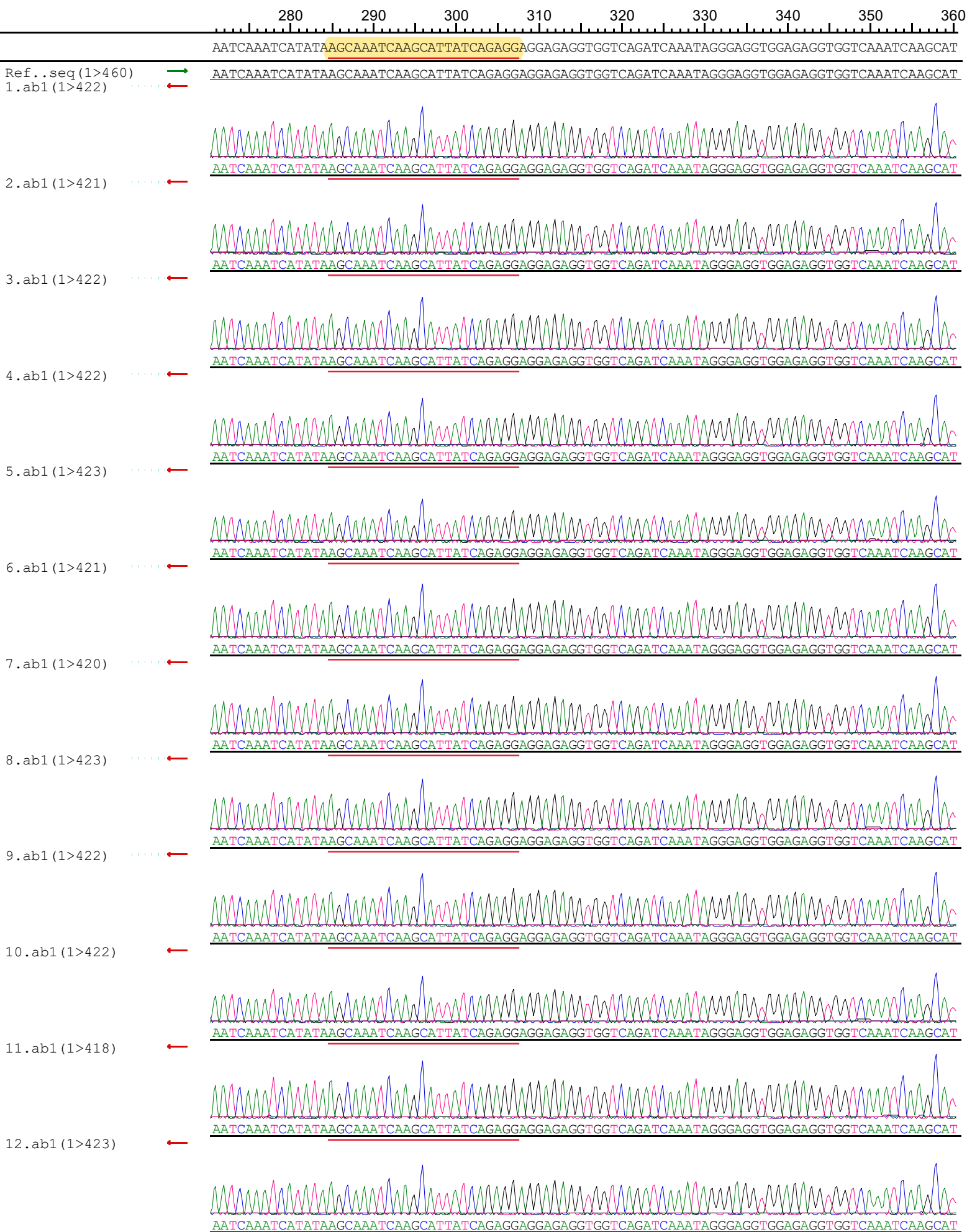

Project: CABVVH010000002.1 489.51 Mbp.ssqd Contig 1

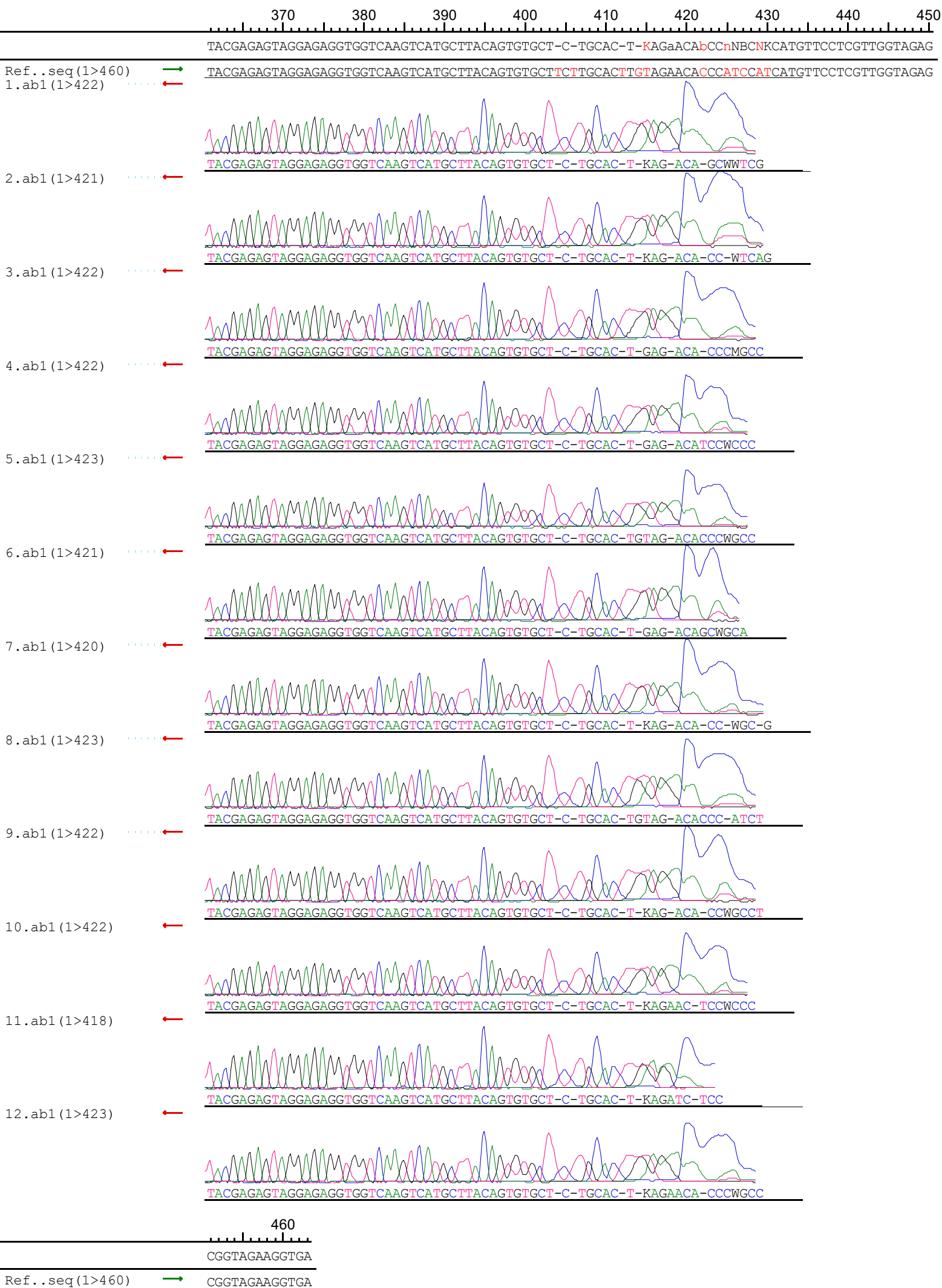

Supplement: Data Sheet 3 — Alignment of the 460-bp nucleotide sequence covering the CABVVH010000002.1 489.51 Mbp off-target site with the respective.ab1 files of 12 analyzed T3-generation plants. [file Data_Sheet_4.PDF]
